# A systematic review and synthesis of theories of change of school-based interventions integrating health and academic education as a novel means of preventing violence and substance use among students

**DOI:** 10.1186/s13643-018-0862-y

**Published:** 2018-11-13

**Authors:** Tara Tancred, Sara Paparini, G. J. Melendez-Torres, James Thomas, Adam Fletcher, Rona Campbell, Chris Bonell

**Affiliations:** 1 Department of Public Health, Environments and Society, London School of Hygiene and Tropical Medicine, 15–17 Tavistock Place, London, WC1H 9SH UK; 2 Division of Health Sciences, Warwick Medical School, University of Warwick, Coventry, CV4 7AL UK; 3 Evidence for Policy and Practice Information and Co-ordinating Centre (EPPI-Centre), Social Science Research Unit, University College London Institute of Education, WC1H ONR, London, UK; 4 Development and Evaluation of Complex Interventions for Public Health Improvement (DECIPHer), School of Social Sciences, Cardiff University, Cardiff, CF10 3WT UK; 5 Department of Population Health Sciences, University of Bristol, 39 Whatley Road, Bristol, BS8 2PS UK

**Keywords:** Adolescent health, Prevention, Education, Qualitative research, Theory, Systematic review

## Abstract

**Background:**

Schools can play an important role in promoting health. However, many education policies and institutions are increasingly emphasising academic attainment targets, which appear to be diminishing the time available for health education lessons. Interventions that integrate both health and academic learning may present an ideal solution, simultaneously addressing health education and academic development. The theories of change underlying these interventions are therefore of interest, but are poorly studied.

**Methods:**

A systematic review of evaluations of interventions that integrate academic and health education for reduced substance use and/or violence was carried out. As part of this, reports describing theory were assessed for quality and data extracted. Theoretical data were synthesised within and across individual interventions using reciprocal translation and meta-ethnographic line of argument synthesis to produce an overall theory of change for interventions that integrate health and academic education to prevent substance use and violence.

**Results:**

Forty-eight reports provided theoretical descriptions of 18 interventions. An overarching theory that emerged was that eroding ‘boundaries’ at multiple and mutually reinforcing levels—by integrating academic and health education, by transforming relationships between teachers and students, by generalising learning from classrooms to the wider school environment and by ensuring consistent messages from schools and families—is intended to lead to the development of a community of engaged students oriented towards pro-social behaviour and away from substance use, violence and other risk behaviours.

**Conclusions:**

Eroding ‘boundaries’ between health and academic education, teachers and students, classrooms and the wider school and schools and families were seen to be the most critical to establishing new frameworks of family, classroom or school organisation that are conducive to promoting both academic and social-emotional outcomes. Whether such interventions are feasible to implement and effective in reducing risk behaviours will be examined in other reports arising from the review.

**Electronic supplementary material:**

The online version of this article (10.1186/s13643-018-0862-y) contains supplementary material, which is available to authorized users.

## Background

Schools can play a critical role in improving young people’s health [1–5]. Given the emphasis in many education policies and institutions on academic attainment targets and diminished time for health education [6–10], interventions which combine health and academic education have sought a platform within academic curricula [11, 12]. Such programmes tend to focus on social and emotional learning, recognising the mutually reinforcing benefits between the development of social and emotional skills and academic outcomes through an interplay of cognitive and non-cognitive skill development [13, 14].

Young people’s use of substances (alcohol, tobacco and other drugs) is an important public health issue [15]. Alcohol use is associated with unsafe sexual behaviour, unintended pregnancies, accidents/injuries and violence [16]. Of smokers, 40% start in secondary school [17] and early initiation is associated with heavier and more enduring smoking and greater mortality. Young people’s drug use is associated with accidental injury, self-harm, mental ill health and suicide [18–20], and other ‘problem’ behaviours [15, 21–24].

The prevalence, harms and costs of violence among young people mean that addressing this is a public health priority [25, 26]. By age 15–16, 24% of students report that they have carried a weapon and 19% reported attacking someone with the intention to hurt them seriously [27]. There are links between aggression and anti-social behaviours in youth and violent crime in adulthood [28, 29].

Many of the programmes integrating health and academic education aim to reduce violence or substance use, with potentially synergistic effects due to shared risk factors [30–32]. These programmes may allow for a larger intervention ‘dose’ and may provide opportunities for reinforcement of learning across subjects [33–35]. While delivered primarily in the classroom, many such programmes also seek to improve the overall school culture and some extend their reach to parents and carers, engaging them in reinforcing and modelling behaviour and skills taught in the classroom.

Several authors have previously drawn together descriptions of the theoretical aspects of social and emotional skills programmes, some of which aim to be integrated with academic teaching. For example, Flay et al. [36] suggest key common aspects across such programmes’ conceptual frameworks, including the need for change in the school-wide climate, training of staff and students in behavioural and environmental considerations, providing opportunities for social modelling, provision of direct instruction and parent and community involvement. Haegerich and Metz [37] drew out a common conceptual framework that outlines how developing social and emotional competencies might improve behaviours, which then positively influence academic outcomes. This framework also emphasises the importance of the interventions affecting school climate, suggesting they can impact students’ feelings of connectedness to their school settings, safety when at school and parental involvement, also contributing to improved behaviours and academic outcomes. Of note, however, is that these reviews were not systematic and do not clearly distinguish between interventions that do or do not aim for the integration of social and emotional learning with academic education.

To our knowledge, there have been no systematic reviews of programmes that integrate academic and health education. Therefore, there is limited available information on the effectiveness of these programmes, the theories of change that drive them or the factors that may affect their implementation. Understanding theory is critical for effective programme development [38, 39]. Interventions that integrate academic and health education for reduced substance use and/or violence have not previously been identified in the literature as a discrete category. Therefore, before synthesising evidence on process and outcomes, a distinctive theory of change must be established. Assuming the existence of a distinctive theory of change, it is then possible to define the intended mechanisms of the intervention and hypothesise how these might interact with context to generate outcomes. We will thus start the process of developing a realist theory of change, refining this via the synthesis of process evaluations and data allowing it to be tested via the synthesis of outcome evaluations.

As a first step, and within the context of a broader systematic review also synthesising evidence from process and outcome evaluations of school-based interventions that integrate health and academic education to reduce violence or substance use among children and young people, we used meta-ethnographic methods to synthesise theories of change for such interventions. We present the results of that synthesis here.

## Methods

### Synthesising theory: some key considerations

Methodological challenges and opportunities posed by syntheses of theories of change have previously been noted [40–43]. Methods for theory synthesis tend to draw on methodological approaches to synthesising empirical qualitative data (or ‘qualitative synthesis’) [44]. Qualitative synthesis, in turn, draws mainly from qualitative data analysis [45]. Broadly, common principles and practices apply to different approaches to qualitative analysis and synthesis, including data immersion, an emphasis on depth, iterative coding of recurrent themes or concepts, consensus-reaching among multiple researchers and the purposive inclusion of ‘deviant’ cases in the analysis [46].

Although there have been some attempts at developing and using checklists to appraise the quality of theories of change [40], there is no single well-established tool [47]. There are also challenges when applying methods used to synthesise empirical qualitative data (generally context-specific) to the synthesis of theory (often abstracted from context) [48]. However, we considered that synthesis can be achieved, for example, by treating theory data as *primary* data in itself [40]. Previous studies have used meta-ethnography to synthesise non-ethnographic data [40, 48, 49]. This approach was the one we took to synthesise theories of change here. Further, we considered that meta-ethnography would be appropriate for the literature on interventions that integrate health and academic curricula, as this is a fairly cohesive field of study [41]. Finally, it is a method that enables conceptual synthesis, making it particularly suitable for theory synthesis.

These challenges and considerations informed our use of a two-stage synthesis process—coding and synthesising theories of change *within* intervention studies, then across them—prior to the development of a ‘line of argument’ synthesis that could offer a representation of theoretical constructs in ‘different stages along the same causal pathway’ ([47], p. 8). This process is described in detail in the methods of theory synthesis section below.

### Methods of systematic review

A full protocol outlining the methods of this systematic literature review, which followed the Preferred Reporting Items for Systematic Reviews and Meta-Analyses (PRISMA) guidelines [50], is available: https://www.journalslibrary.nihr.ac.uk/programmes/phr/145215/#/ The study was registered with the PROSPERO registry of vsystematic reviews (reference 2015:CRD42015026464) available from: http://www.crd.york.ac.uk/PROSPEROFILES/26464_PROTOCOL_20160011.pdf. Briefly, a systematic review was undertaken of evaluations of school-based interventions delivered: within classroom settings; in regular school hours; and through (at least in part) the integration of academic and health education aiming to prevent substance use or violence among children and young people age 5–18 years. Included studies were process evaluations or outcome evaluations, the latter employing random allocation to intervention and control groups (implementing usual or no treatment), with no restrictions by country. Descriptions of theory of change within, or otherwise linked to, empirical evaluations were synthesised. From November to December 2015, various health, social science and education databases were searched (please see protocol for full listing). Searching of webpages and reference lists of relevant studies for further references and consultation with experts in this field followed. Searches were updated using PsycINFO and the Cochrane Central Register of Controlled Trials. Searches relating to outcome evaluations of interventions targeting violence were updated on 28 February 2018 and those relating to outcome evaluations of interventions addressing substance use were updated on 14 May 2018. Searches for process evaluations were updated on 8 September 2018.

These are listed in Table 1.

**Table 1:** Summary of included reports and interventions of the theory synthesis

Intervention name	Description of intervention	Country	Targeted grade and age of participant	Integration	Report
4Rs (Reading Writing, Respect and Resolution)	A literacy-based social-emotional learning curriculum for elementary school students. There are two components: (1) a seven-unit, 21–35 lesson literacy-based curriculum in conflict resolution and social-emotional learning for children in primary school (to grade 5); and (2) intensive professional development for teachers.	USA	Kindergarten to grade 5 (ages 5–11)	Each unit uses children’s literature is used to help students understand anger and develop skills in listening, cooperation, assertiveness, and negotiation. Additionally, 4Rs aims to develop literacy skills in children to capitalise on the mutual positive effect that social-emotional and academic learning have on each other.	Aber 2011 [61]
					Brown 2010 [51]
					Flay 2009 [36]
					Jones 2008 [52]
					Jones 2010 [75]
					Jones 2011 [59]
					Sung 2015 [76]
Bullying Literature Project	This programme aims to reduce bullying by introducing themes related to bullying through children’s literature. It also provides an opportunity for children to role-model practical skills to address or avoid bullying.	USA	Grade 4 (ages 9–10)	The Bullying Literature Project integrates themes related to bullying into the children’s literature used within a standard English curriculum.	Couch 2015 [77]
					Wang 2015 [78]
					Wang 2017 [79]
DRACON	This programme uses drama to develop cognitive understanding of conflict and bullying and to empower students to manage their own conflict, both personally and within the broader school community.	Australia	Primary and secondary school students (ages 5–18)	Conflict literacy is taught through ‘enhanced forum theatre’ and other drama techniques.	Malm 2007 [60]
English classes (no name)	Teachers were trained, and working in pairs in the summer, they developed integrated health/English material, with a specific emphasis on the prevention of drug and alcohol use.	USA	Grades 8 and 9 (ages 13–15)	Health topics were infused into English classes. English was chosen as it was felt to be the subject into which non-traditional concepts could be discussed and is taken by all students.	Holcomb 1993 [66]
Hashish and Marijuana	The goal of the curriculum is to develop scientific knowledge of hashish and marijuana and to strengthen students’ problem-solving and decision-making skills through both didactic and participatory learning approaches.	Israel	Upper secondary school (ages 15–18)	The programme was entirely integrated into chemistry classes, where lessons around hash and marijuana teach the chemical aspects of the drugs. Behaviour change was addressed through more participatory teaching methods.	Zoller 1981 [80]
Infused-Life Skills Training (I-LST)	This intervention integrates the messaging of standard life skills training (self-image and self-improvement, decision-making, smoking, marijuana, alcohol, etc.) into core academic classes like English, science, maths, and so forth for healthier behaviours, including reduced substance use.	USA	Middle/junior high school (ages 12–15)	The entire interventions hinges on the effective infusion of health messaging into the core academic curriculum.	Bechtel 2006 [67]
Kids, Adults Together (KAT)	This programme has a classroom component in which children learn about alcohol, a parent evening in which students prepare presentations for parents and a take-home DVD about alcohol that is to be watched with parents for normalisation of reduced alcohol use.	UK	Grades 5 and 6 (ages 10–12)	The classroom curriculum is delivered across many subjects and students develop academic skills alongside understanding of alcohol at every opportunity. For example, in arts classes, students will learn design skills by making anti-alcohol posters.	Segrott 2015 [71]
Learning to Read in a Healing Classroom	The intervention has two components delivered specifically to active treatment schools: teacher learning circles, which are opportunities for teachers to learn from master educators about classroom practices that enhance growth and development, and teacher resource materials, which included literacy components and social-emotional learning components. These resource materials are intended to integrate social-emotional learning alongside learning to read. Materials were provided in French.	Democratic Republic of Congo	Years 3–5 (ages 8–11)	The classroom component includes materials to support teachers in creating safe and supportive learning environments, as well as using literature to teach students emotional regulation, self-expression and how to interact with peers.	Torrente 2015 [81]
					Aber 2017 [82]
Linking the Interests of Families and Teachers (LIFT)	The programme aims to reduce future delinquency by positively rewarding and reinforcing good behaviours. The intervention includes classroom, playground and family components.	USA	Grades 1 and 5 (ages 6–7 and 10–11)	The fifth grade classroom component also develops study skills that align with the fifth grade curricula.	DeGarmo 2009 [83]
					Eddy 2000 [84]
					Eddy 2015 [85]
					Reid 1999 [86]
					Reid 2002 [87]
Peaceful Panels	Throughout art classes, students participated in anti-bullying lessons (from the Second Step program for eighth grade students on empathy and communication in handling a grievance) and comic-making lessons. They then prepared artwork to demonstrate their understanding of how to resolve conflict.	USA	Grades 8 and 9 (ages 13–15)	Conflict resolution lessons were integrated fully within art classes and creation of art was used as a medium to reflect on learning.	Wales 2013 [88]
Positive Action	Positive Action is a social-emotional and character development programme aimed at encouraging positive behaviours through positive thoughts and actions. Lessons cover six units: self-concept; positive actions for mind and body; positive social-emotional actions; managing oneself; being honest with oneself; and continually improving oneself.	USA	Kindergarten to grade 12 (ages 5–18)	The methods of education used promote active learning and learning skills development alongside behaviour change. Teachers are also encouraged to tie lessons into academic content.	Beets 2008 [89]
					Beets 2009 [90]
					Flay 2009 [36]
					Flay 2010 [58]
					Lewis 2012 [91]
					Malloy 2015 [92]
Promoting Alternative Thinking Strategies (PATHS)	PATHS aims to develop social and emotional competencies in children for reduced aggression and behaviour problems. Throughout the elementary school years, students receive lessons grouped under three units: readiness and self-control; feelings and relationships; and inter-personal problem solving.	USA	Kindergarten to grade 5 (ages 5–11)	Both reading and writing are bridged to PATHS in most lessons by including supplementary suggestions for teachers to utilise such things as quality children’s literature to reinforce lesson concepts. Further, teachers can directly tie PATHS concepts to English, social studies/history and other subject areas.	Crean 2013 [93]
					Flay 2009 [36]
					Greenberg 2006 [62]
					Kusché 2012 [94]
Raising Healthy Children	A four-component (school, student, peer and family) intervention that promotes positive youth development by targeting risk and protective factors at different stages of development. The focus is around prosocial bonding, social-emotional learning, and the development of problem-solving skills.	USA	Grades 1–8 (ages 6–14)	Staff were trained to promote reading as part of school intervention strategies. Also, in grades 4–6, there were after-school study groups and group-based workshops in high school, all of which aimed to increase academic achievement	Brown 2005 [69]
					Catalano 2003 [95]
Roots of Empathy	A programme that brings a visiting baby and their parent into a classroom as a springboard for learning empathy. Students learn messages of social inclusion, respect, how to build consensus, how to contribute to a safe and caring classroom, and develop emotional literacy.	Australia, Canada, UK	Grades 1–9 (ages 6–15)	Connections with literacy, writing, art, music, mathematics and science are made. Lessons may involve literature to encourage children to explore their own emotions and experiences. Children may also make artwork to reflect these feelings, which is often compiled and used in follow-up activities.	Cain 2008 [64]
					Gordon 2003 [96]
					Hanson 2008 [97]
Second Step	The intervention was delivered by teachers after implementation training. The dose includes 15 weeks of classroom lessons taught weekly or every 2 weeks throughout the school year for 3 years. The intervention consists of manualised content including didactic lessons, group activities, and multimedia content including modelling of skills.	USA	Grade 6 (ages 11–12)	Students receive academic homework integration assignments, and teachers are encouraged to connect lessons to current events.	Espelage 2013 [98]
					Espelage 2015a [99]
					Espelage 2015b [100]
					Farrell 2015 [101]
					Low 2016 [102]
Steps to Respect	A bullying prevention programme that has both a classroom curriculum aimed at teaching emotional intelligence, bullying prevention and bystander skills to children and a schoolwide component that trains teachers and administrators to change policies around disciplining bullying and providing mentoring to prevent future occurrences	USA	Grades 3–6 (ages 8–12)	In the classroom curriculum, children’s literature is used to frame lessons and develop social-emotional and anti-bullying skills	Brown 2011 [70]
					Frey 2009 [103]
The Gatehouse Project	Through teaching a curriculum and establishing a school-wide adolescent health team, Gatehouse aims to: build a sense of security and trust in students; enhance skills and opportunities for good communication; and build a sense of positive regard through participation in school life.	Australia	Grade 8 (ages 13–14)	The program adopts a critical literacy approach. Concepts are taught using literature, poetry, song, film and visual materials. There is a deliberate link made between the programme’s goals and academic goals.	Bond 2008 [104]
					Patton 2000 [65]
					Patton 2003 [105]
Youth Matters	Youth Matters promotes the development of healthy relationships between students, staff and schools. It also promoted skills around social resistance and social competency through a curriculum. The curricula also emphasised both the bully and the victim perspective.	USA	Grades 4 and 5 (ages 9–11)	Each module uses a 30–40-page story that is intended to help schools meet academic standards in both health education and English.	Jenson 2007 [106]

### Data extraction

Data were extracted from all included reports featuring theories of change. Reports that included very minimal information on theory or merely repeated information on theory present in previously published reports already included in our review were excluded and only the earliest report presenting this information was included. All reports that added new understanding of the theoretical basis for an intervention were included. Data extraction included basic study information (e.g. location, duration, participant characteristics, intervention description) as well as theory (e.g. a description of integration of health and academic education, existing theories referred to and description of any theories of change, logic models, conceptual frameworks, or underlying theoretical assumptions pertaining to how the interventions were expected to function).

### Quality appraisal

All reports were assessed for quality using a tool adapted from a previous review [41]. This tool assessed the clarity of constructs and of the relationships between these constructs, whether the theory was logical, based on any prior evidence and potentially testable empirically, whether the theory was developed in concert with practitioner or community stakeholders and whether the theory appeared to be potentially applicable to different contexts. Overall, there were ten indicators of quality, and two reviewers independently judged whether those were present or absent based on the study’s description of theory. See Table 2 for a summary of our quality appraisal of included studies. This process aimed to enable us to give greater weight to those reports with a more robust description of theory.

**Table 2:** Summary of the quality appraisal of included studies by reviewer

Intervention name	Clarity				Plausibility and feasibility				Testability	Ownership					Generalisability						Overall score (%)
	Constructs defined		Clear pathways from inputs to outcomes		Theory logical (pathways logical)	Empirical evidence in support of theory			Evidence of empirical testing of the theory	Theory developed by practitioners			Theory developed by community members		Theory presented as general		Theory describes its application to different contexts		Authors prevent evidence of a theory’s generalisability		
	CB	TT	CB	TT	CB	TT	CB	TT	CB	TT	CB	TT	CB	TT	CB	TT	CB	TT	CB	TT	
4Rs Aber 2011	N	N	N	Y	Y	Y	Y	Y	Y	Y	Y	Y	N	N	Y	N	Y	N	N	N	55
Brown 2010	Y	N	Y	N	Y	Y	Y	Y	Y	Y	Y	N	N	N	Y	N	Y	N	N	N	55
Flay 2009	Y	Y	Y	Y	Y	Y	N	N	Y	Y	Y	N	N	N	Y	Y	N	N	N	N	55
Jones 2008	Y	Y	N	Y	Y	Y	Y	Y	Y	Y	Y	N	N	N	Y	Y	N	Y	N	Y	70
Jones 2010	N	Y	Y	Y	Y	Y	Y	Y	Y	Y	Y	N	N	N	Y	Y	Y	N	Y	N	70
Jones 2011	N	Y	N	Y	Y	Y	Y	Y	Y	Y	Y	N	N	N	Y	Y	Y	N	N	N	60
Sung 2015	N	Y	N	N	N	Y	Y	N	Y	N	Y	N	N	N	Y	N	Y	N	N	N	35
Bullying Literature Project Couch 2015	N	N	N	N	N	N	Y	Y	Y	Y	N	N	N	N	Y	Y	N	N	N	N	30
Wang 2015	Y	Y	Y	Y	Y	Y	Y	N	Y	Y	N	N	N	N	Y	N	N	N	N	N	50
Wang 2017	Y	Y	Y	Y	Y	Y	Y	N	N	Y	N	N	N	N	N	Y	N	N	N	N	50
DRACON Malm 2007	N	Y	N	Y	Y	Y	N	N	N	Y	Y	Y	Y	N	Y	N	Y	N	Y	N	55
English classes (no name) Holcomb 1993	N	Y	N	Y	N	Y	N	N	N	Y	Y	N	N	N	N	Y	Y	N	N	N	35
Hashish and Marijuana Zoller, 1981	N	Y	N	Y	N	Y	N	N	Y	N	N	N	N	N	N	Y	N	N	N	N	25
I-LST Bechtel 2006	N	N	N	N	Y	N	Y	N	Y	N	N	N	N	N	Y	Y	N	N	N	N	25
KAT Segrott 2015	N	N	N	Y	N	Y	N	Y	Y	Y	N	N	N	N	Y	Y	N	N	N	N	35
Learning to Read in a Healing Classroom Torrente 2015	Y	Y	Y	Y	Y	Y	Y	Y	Y	Y	Y	Y	N	N	N	N	Y	Y	Y	Y	90
Aber 2017	Y	Y	Y	Y	Y	Y	Y	Y	Y	Y	Y	Y	N	N	N	N	Y	Y	Y	Y	90
LIFT DeGarmo 2009	Y	N	Y	Y	Y	Y	Y	Y	Y	Y	N	N	N	N	Y	Y	N	N	Y	N	60
Eddy 2000	N	N	N	Y	Y	Y	Y	N	Y	N	N	N	N	N	N	Y	N	Y	N	N	35
Eddy 2015	N	Y	Y	N	Y	Y	Y	Y	Y	Y	N	N	N	N	Y	Y	Y	N	N	N	55
Reid 1999	Y	Y	Y	Y	Y	Y	Y	Y	Y	Y	Y	N	N	N	Y	Y	N	N	N	N	65
Reid 2002	N	N	N	Y	N	Y	Y	N	Y	Y	Y	N	N	N	Y	Y	Y	N	N	N	45
Peaceful Panels Wales 2013	N	N	N	Y	N	N	N	N	N	N	Y	N	N	N	Y	N	N	N	N	N	15
Positive Action Beets 2008	N	Y	N	Y	Y	Y	N	N	N	Y	N	N	N	N	Y	N	N	N	N	N	30
Beets 2009	N	N	N	N	N	N	Y	N	Y	N	N	N	N	N	Y	Y	N	N	N	N	20
Flay 2009	N	Y	N	Y	Y	Y	Y	Y	Y	Y	N	N	N	N	Y	Y	N	Y	N	Y	60
Flay 2010	N	Y	Y	Y	Y	Y	Y	N	Y	Y	Y	N	N	N	Y	Y	Y	N	N	N	60
Lewis 2012	N	Y	N	Y	N	Y	N	N	Y	N	N	N	N	N	N	Y	N	N	N	N	25
Malloy 2014	N	N	N	N	N	Y	N	N	Y	Y	N	N	N	N	Y	Y	N	N	N	N	25
Malloy 2015	N	N	N	N	N	Y	N	N	Y	Y	N	N	N	N	Y	Y	N	N	N	N	25
PATHS Crean 2013	N	Y	N	Y	Y	Y	Y	N	Y	Y	N	N	N	N	Y	Y	N	N	N	N	45
Flay 2009	Y	Y	N	N	N	N	Y	Y	Y	N	N	N	N	N	Y	Y	N	N	N	N	35
Greenberg 2006	Y	Y	Y	Y	Y	Y	Y	Y	Y	N	N	N	N	N	Y	Y	N	N	Y	N	60
Kusché 2012	N	Y	Y	N	Y	Y	Y	N	Y	N	N	N	N	N	Y	Y	N	N	Y	N	45
Raising Healthy Children Brown 2005	N	Y	Y	Y	Y	Y	Y	Y	Y	Y	N	N	N	N	Y	Y	Y	N	N	N	60
Catalano 2003	Y	N	Y	N	Y	N	Y	N	Y	Y	N	N	N	N	Y	Y	N	N	N	N	40
Roots of Empathy Cain 2008	N	Y	N	Y	Y	Y	Y	N	N	N	Y	N	N	N	Y	Y	N	N	N	N	40
Gordon 2003	N	Y	N	N	N	Y	Y	N	Y	Y	Y	N	N	N	Y	Y	N	N	N	Y	45
Hanson 2008	N	N	Y	N	Y	Y	Y	N	Y	Y	Y	N	N	N	Y	Y	N	N	Y	N	50
Second Step Espelage 2013	Y	Y	Y	Y	Y	Y	Y	Y	Y	Y	Y	Y	N	N	N	N	N	N	N	N	60
Espelage 2015a	Y	Y	Y	Y	Y	Y	Y	Y	Y	Y	Y	Y	N	N	N	N	N	N	N	N	60
Espelage 2015b	Y	Y	Y	Y	Y	Y	Y	Y	Y	Y	Y	Y	N	N	N	N	N	N	N	N	60
Farrell 2015	Y	Y	Y	Y	Y	Y	Y	Y	Y	Y	Y	Y	N	N	N	N	N	N	N	N	60
Low 2016	Y	Y	Y	Y	Y	Y	Y	Y	Y	Y	Y	Y	N	N	N	N	N	N	N	N	60
Steps to Respect Brown 2011	Y	Y	Y	N	Y	N	Y	Y	Y	Y	N	N	N	N	Y	Y	N	N	Y	N	55
Frey 2009	N	Y	Y	Y	Y	Y	Y	Y	Y	Y	N	N	N	N	Y	Y	N	N	N	N	55
The Gatehouse Project Bond 2008	N	N	N	Y	Y	Y	N	N	Y	Y	N	N	N	N	Y	Y	N	N	N	N	35
Patton 2000	N	Y	N	Y	Y	Y	Y	N	Y	N	N	Y	N	Y	Y	Y	Y	N	Y	N	60
Patton 2003	N	N	N	N	N	N	Y	N	Y	N	N	N	N	N	Y	Y	N	N	N	N	20
Youth Matters Jenson 2007	N	Y	N	Y	Y	Y	N	N	Y	Y	N	N	N	N	Y	Y	N	N	N	N	40

### Methods of theory synthesis

As a preliminary step, each individual intervention theory was subjected to within-intervention coding and synthesis. These theories were then synthesised across all interventions to explore points of reciprocal resonance, refutation and/or complementarity among them. This approach led us to generate a line of argument within the ‘across intervention’ theory synthesis.

#### Within-intervention coding

To pilot our procedures, two higher quality (i.e. having quality scores above 50%, please see Table 2) ‘pilot’ studies by different authors but focused on the same intervention were read and re-read by two reviewers [51, 52]. Line-by-line codes were applied and memos written up to capture and explain the content of the descriptions of theory. Codes were then grouped, organised in frameworks and exchanged and compared among reviewers to develop an overall set of codes. This set of codes was then applied to the remaining study reports of the intervention in question including description of theory of change. Reviewers kept track of and compared any modifications to the coding framework made from the coding of subsequent studies. Having judged that this piloted process was appropriate, this process was then applied to each intervention in the review.

#### Within-intervention synthesis

The next step in the analysis was to synthesise programme theories for each individual intervention. This later allowed us to build on these to develop an overall synthesis, which is the main focus of this paper [48]. For the within-intervention synthesis, reciprocal translation, a dynamic, iterative process where concepts from each report were ‘translated’ into one another, was used [48, 53, 54]. We identified commonalities, differences of emphasis and contradictions between multiple papers reporting on each intervention’s theory, collated sets of codes from the two reviewers and resolved any apparent inconsistencies by discussion or refinement of the emerging synthesis. A third reviewer helped achieve reconciliation, where necessary, and discussed and triangulated the analysis. Where only one report described theory for an intervention, it was taken to represent the theory for that intervention.

#### Across-intervention synthesis

We employed a meta-ethnographic approach in the third phase of analysis to synthesise individual programme theories’ mechanisms of change to develop a line of argument, as far as possible, for the overall category of intervention that integrates health and academic education to prevent substance use and violence. In this approach, the key concepts extracted during the first (within-intervention) coding exercise were treated as first-order constructs [55] because they reflected closely the authors’ opinions and views of the world, rather than being their interpretation of empirical data, which could have been considered as second-order constructs, as in most qualitative syntheses [40, 56]. Our first-order constructs appear in the first column of Table 3. Second-order constructs were then generated, which were our interpretation of authors’ views. Finally, third-order constructs were developed to produce a line of argument that aimed to describe the overall mechanisms of change taking place within these interventions.

**Table 3:** First-, second- and third-order constructs

First-order constructs	Second-order constructs	Third-order constructs	Overall social/psychological process (line of argument)
Academic platform used to simultaneously build academic and health skills	Degree of integration of academic and health curricula (full or partial)	Eroding boundaries between health and academic education	Eroding boundaries at multiple and mutually reinforcing levels—by integrating academic and health education, by promoting stronger, more affective relationships between teachers and students, by generalising learning from classrooms to the wider school environment, and by ensuring consistent messaging between schools and families—will lead to the development of a community of engaged students oriented towards pro-social behaviour and away from substance use, violence and other risk behaviours.
Health curricula bridged into academic curricula, with academic curricula sometimes intended to reinforce health messages and vice versa			
Decreasing time during the school day for health education due to an emphasis on academic achievement (measured through standardised test scores)	Pragmatic rationale for integration		
Mutually reinforcing effect of improved academic and health outcomes	Scientific rationale for integration		
Students being less resistant to health messaging in integrated curricula	Additional rationale for integration		
Integrated curriculum providing opportunities for repetition			
Opportunities for experiential learning provided			
Teachers’ internalisation of curriculum’s messages	Normalisation of prosocial behaviours through teacher internalisation of curricula	Eroding boundaries between teachers and students	
Teachers’ role-modelling of curriculum’s promoted behaviours			
Forming bonds between prosocial peers and adults and acceptance of behaviours demonstrated within these relationships			
Establishment of good relationships between students and teachers	Establishing student connectedness to the classroom through positive teacher-student relationships		
Rewards for prosocial behaviours being given to students	Positive reinforcement of prosocial behaviours	Eroding boundaries between classroom and the wider school	
Students internalising prosocial beliefs and feeling positive about themselves following the demonstration of prosocial behaviours, leading to more prosocial behaviours			
Students having the opportunity to practice valued skills at multiple levels			
A sense of connectedness and bonding with the school is linked to overall emotional wellbeing and security experienced by students in school	Establishing student connectedness to the school		
Connectedness to school fosters better academic learning as the school becomes a more positive environment that students are invested in			
Role modelling of prosocial behaviours at home	Normalisation of prosocial behaviours	Eroding boundaries between schools and families	
Parents aware of curricula and expect prosocial behaviours (e.g. conflict resolution skills) to be practiced at home	Provision of opportunities for practical skill development		

Developing a line of argument was challenging because programme theories described similar notions, but at times offered limited explanation for key concepts and assumptions, so our second-order constructs already entailed a significant degree of abstraction. In working towards our third-order constructs, it was useful to draw from existing theoretical frameworks to develop a coherent overall view of what initially appeared to be disparate concepts. In particular, the notion of ‘boundary erosion’ (put forward by Markham and Aveyard [57] as a key process to promoting health in schools) was applied, which sensitised us to ways in which concepts from the individual syntheses could be synthesised into a set of third-order constructs to develop an overall line of argument. These are detailed in the third column in Table 3.

A few themes did not fit with the concept of boundary erosion as presented by Markham and Aveyard. These stimulated us to refine and expand the concept of boundary erosion so that it could encompass these apparently divergent themes [54]. These are presented alongside our line-of-argument synthesis in the Discussion section.

Table 3 was adapted from Britten et al.’s worked example of meta-ethnography [53] and was used and modified repeatedly by two reviewers to build up the line of argument of the overall synthesis of the theory of change for the interventions included in our systematic review, expressed in the final column.

## Results

**Fig. 1:**
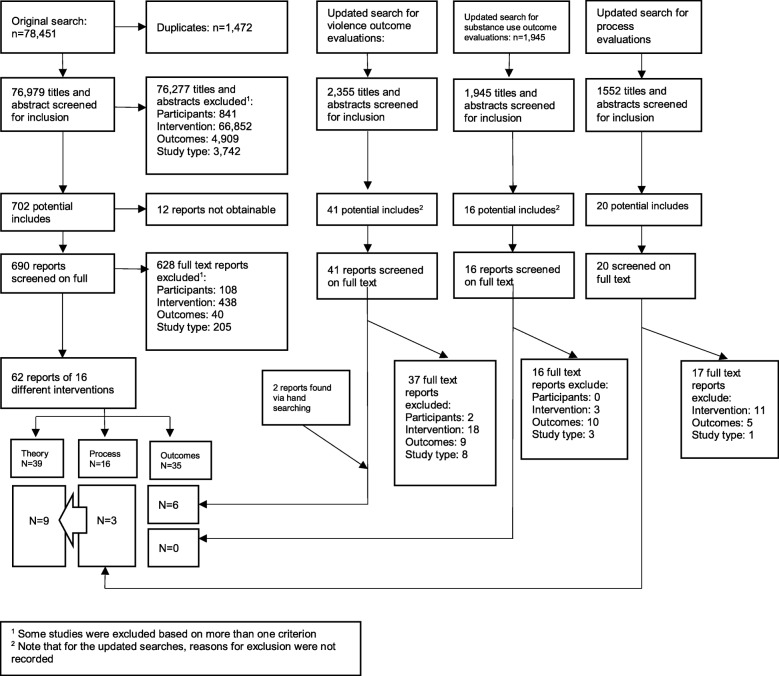
Screening outcomes and included studies

Our original searches generated 76,979 unique titles and abstracts that were screened for possible inclusion in the study. From these, we identified 62 relevant reports in this study, of which 39 included some description of theory of change, covering 16 different interventions. Updated searches for outcome evaluations retrieved an additional 2355 and 1945 references, yielding an additional six reports of outcome evaluations. Those for process evaluations retrieved an additional 1552 references yielding an additional three reports of process evaluations. All nine studies included some description of theories of change. Figure 1 reports on the screening procedure for the overall review, indicating which study reports were included in the syntheses of theories of change, as well as the process and outcome evaluations from which these were drawn. Table 1 below summarises the reports that were quality assessed and data extracted for this review. Flay et al. [36] describes three interventions and is therefore repeated, so there are a total of 48 unique reports. Together, these reports provided the theoretical description of 18 of the interventions included in the overall systematic review.

### Study quality

Table 2 below summarises the quality appraisal of the components of each report included in this study that emphasised theory. The combined appraisal for each study ranged from quite poor (20% of quality indicators judged to be present by reviewers) to very good (90% of quality indicators judged to be present by reviewers). There was some disagreement between the two reviewers carrying out the quality assessment, with an overall agreement rate of 71%.

The remainder of the results present our synthesis of theories of change for the overall category of interventions integrating health and academic education to prevent violence or substance use. Because of the poor inter-rater consistency of quality appraisal, we do not refer below to summaries of the quality of theories of change in the synthesis. Our synthesised theories of change for each individual intervention are not reported here but can be found in Additional file 1.

### Programme inputs

Our review focused on school-based interventions and these overwhelmingly involved health curricula delivered in classrooms. Some programmes also included after-school or out-of-school components, while others had whole-school or family components. As an example of a multi-component intervention, the Positive Action curriculum:

*Teaches specific positive actions for the whole self: the physical, intellectual, social, and emotional areas. Together, these make up the comprehensive set of skills for successful learning and living…The program trains teachers and parents to identify, teach, and reinforce positive thoughts, actions, and feelings about themselves by students and others in the school, leading to continual reinforcement of positive actions and enhanced student bonding with parents and school*. ([58], pp.476–477)

Some interventions required the training of teachers or other staff in delivery of the curriculum. Some curricula were externally facilitated and therefore no internal training of school staff was required.

Curricula addressed a wide variety of topic areas and desired skills. Of note, only two interventions dealt directly with substance use. Most curricula concentrated on conflict resolution, problem-solving skills and social-emotional skills like emotional intelligence and empathy in students, as an example from 4Rs indicates here:

*The approach of embedding social-emotional learning and conflict resolution lessons in a balanced literacy delivery strategy, and research tying together the social-emotional and academic domains, support our expectation for longer term effects on behavior and academic achievement*. ([59], p. 536)

### Mechanisms of change: improving health by eroding boundaries

Through our application of a meta-ethnographic approach, an overarching line of argument that emerged from our analysis was that *eroding boundaries at multiple and mutually reinforcing levels—by integrating academic and health education, by strengthening relationships between teachers and students, by generalising learning from classrooms to the wider school environment, and by ensuring consistent messages from schools and families—will lead to the development of a community of engaged students oriented towards pro-social behaviour and away from substance use, violence and other risk behaviours.*

#### Eroding boundaries between health and academic education

A recurring theme was that interventions integrated and thereby eroded the boundaries between health and academic education. There were two types of integration, which we have defined here as ‘full’ integration and ‘partial’ integration.

In the full integration approach, health education was completely integrated into existing academic subject classes, aiming to build both academic and health knowledge and skills seamlessly and simultaneously. An example of this integration is DRACON [60], which used drama classes as a medium for building drama skills while also aiming to enable children to learn conflict resolution skills intended as a means of reducing aggression. Another example of a fully integrated curriculum was 4Rs, which ‘uses high-quality children’s literature as a springboard for helping students gain skills and understanding in several areas including handling anger, listening, cooperation, assertiveness, and negotiation (…) ensuring students understand the primary themes of the story and allowing them to connect the themes to their own lives.’ ([61], p. 414)

In the second approach, partial integration, interventions addressing substance use or violence were delivered within the normal school curriculum but were treated as discrete modules or programmes, not weaved into normal academic education. What made them ‘integrated’ was simply that while the interventions mainly addressed the knowledge and skills necessary to avoid substance use and violence, they also included some academic elements, regarded as important to address the range of determinants of substance use or violence.

An example of this type of integration is PATHS, which, in its grade five curriculum, included literacy components. However, the PATHS curriculum is primarily focused on building non-academic skills, and although the curriculum is delivered during normal school hours within academic classes, these classes are distinct from the normal academic curriculum.

*Language arts (both reading and writing) are bridged to PATHS in most lessons by including supplementary suggestions for teachers to utilize such things as quality children’s literature to reinforce lesson concepts. Further, one of the chapters in the Instructional Manual provides many ideas for how teachers can directly tie PATHS concepts to language arts, social studies/history, and other subject areas.* ([62], p. 404)

Two common themes emerged in the synthesis as reasons why integration of health and academic curricula was attempted. The more *pragmatic* rationale referred to the decreasing time in the school day dedicated to teaching topics such as health, wellness or conflict resolution. This theme resonated particularly strongly in American programmes delivered after the 2001 No Child Left Behind Act [63]. This act emphasised academic performance on standardised tests, which was often cited in practice as causing schools to focus less on other aspects of student development. Authors discussed how such interventions:

*Evolved in response to the tension between the movement to reform education between standards-based accountability with its focus on academic achievement, on the one hand (e.g., the policy and practice zeitgeist promoted by the No Child Left Behind Act of 2001), and social and character development, on the other*. ([51], p. 156)

A second, more *scientific* theme suggested that academic performance and health outcomes could be improved simultaneously and that these outcomes were mutually reinforcing. The extract below from Roots of Empathy offers an example of this kind of discussion.

*Schools can be reassured by scientific and pragmatic evidence that time spent implementing [social-emotional learning (SEL)] will enhance the academic success of students (…) Current research shows that SEL programs contribute to the academic success of students as well as to their health and wellbeing*. ([64], p. 54)

In discussions of the rationale for integration, one additional opinion on the state of health education in schools was expressed by investigators of The Gatehouse Project, who maintained that there was a new convergence of academic and health mandates in schools, which may reflect the particular policy context in Australia.

*There are signs that health and educational agendas are converging. There has, for example, been an increasing interest in the forms of schooling that might best meet the educational needs of early adolescents. The call for reform (…) has been driven by the identification of alienation or disengagement of young people as the major barrier to educational success*. ([65], p. 588)

Two additional points concerning the rationale for integration were raised to a much lesser extent, but are likely relevant to most of these programmes. These are as follows: that an integrated curriculum provides opportunities for repetition and reinforcement of health concepts and that, when health messages are not as overt—if explored through literature or other subject matter—students may be less resistant to them [66].

*Optimal learning occurs when information is embedded in meaningful contexts, applications and multiple representations are provided, and there are opportunities for learners to generate personally relevant questions*. ([67], p. 220)

Several interventions aimed to integrate health and academic education by making learning more experiential, rooting learning in active participation and providing opportunities for practical skill development. For example, in the I-LST programme, ‘teachers were also encouraged to be certain that the activities were developmentally appropriate and that the lessons included facilitation, coaching and behavioral rehearsal of skills as well as student-centred learning strategies’ ([67], p. 222).

#### Eroding boundaries between teachers and students

The role of the teacher was central to programme-driven change at all levels (e.g. student, classroom, whole school and family). A recurring theme was that interventions aimed to transform relationships between students and teachers and thus establish a better classroom climate. While this theme resonates with the concept of boundary erosion, it also suggests an elaboration of the latter is required, as has previously been argued [68]. By boundary erosion, what we mean is not an erosion of source of differences between staff and students or a challenge to disparities of power but rather a transformation of the quality and strengthening of relationships so that these move beyond merely the instrumental task of academic education and embrace more affective dimensions and students’ overall development. In the included studies, the positive relationships between teachers and students were often seen as increasing student connectedness to the classroom and school, and invoking a sense of responsibility in students to act in a way that is beneficial for themselves, their peers and their teacher.

*Teacher–student relationships are a joint function of the unique characteristics of children (e.g., their social– cognitive attributions and problem-solving style) and teachers (e.g., their social– emotional abilities and experiences of job stress and burnout) and the cultural norms, values, and practices they bring to the relationship and to the classroom. Together these characteristics contribute to the climate of the classroom.* ([51], p.154)

The establishment of student-teacher bonds was regarded as key to *normalising positive behaviours* in students.

*Once strong bonds are established, individuals will tend to behave in a manner consistent with the norms and values of the individuals and groups with whom they associate. In turn, stronger pro-social bonds support positive belief formation against antisocial behaviors (e.g. adolescent substance use)*. ([69], p. 700)

Teachers’ relationship to the curricula they delivered, specifically, their internalisation of the curriculum’s messages was regarded as important. Teachers invested in the integrated curriculum were expected to role-model desired behaviours expressed within the curriculum, further normalising these for students.

*When teachers embrace and practice the program’s principles and implementation strategies, they establish a set of expectations and norms for behaviors in their classrooms, and children begin using those skills and behaviors*. ([51], p. 157)

Steps to Respect highlights the role of teachers in changing social norms for young people by changing their attitudes about, in this instance, bullying.

*Because bullying is a social process strongly influenced by the reactions and behaviors of peers, the program seeks to change attitudes about the acceptability of bullying through clearly labeling bullying behavior as unfair and wrong, increasing empathy for students who are bullied, and educating students about their responsibilities as bystanders to bullying*. ([70], p. 425)

#### Eroding boundaries between classroom and the wider school

Another recurring theme was the importance of interventions aiming to generalise learning from the classroom to the wider school environment. Engaging students outside of the classroom enabled them to reinforce positive behaviours and to increase their broader sense of connectedness with the school.

Having opportunities at multiple levels for skill reinforcement was emphasised by several programmes. At the school level, providing an environment to reinforce skills or behaviours developed through the curriculum could be achieved by involving non-teaching members of staff in some of the programme training, as in the 4Rs intervention.

*Ecologically oriented programs emphasize not only the teaching of skills, but also the creation of meaningful real-life opportunities to use skills and the establishment of structures to provide reinforcement for effective skill application*. ([62], p. 399)

Positive reinforcement of pro-social skill demonstration could occur via explicit rewards or simply by students feeling good about themselves as a result of practising such behaviours. Positive reinforcement was often described as being strengthened through opportunities for demonstration in different areas of life leading to further reinforcement. Demonstration of positive behaviours was then felt to contribute to a positive sense of self, leading to further positive behaviours. Such processes were regarded as enabling internalisation of curriculum content by students.

*PATHS highlights that, ‘internalization is the primary process utilized in the development of an individual’s conscience. When the outcome is positive, the conscience…works as a powerful system through which a person can ‘take ownership’ and achieve mastery over his or her own impulses and actions*.’ ([62], p. 401)

Many interventions, either directly or indirectly, sought to improve students’ sense of connectedness to the school environment. A sense of connectedness and bonding with the school was seen to be linked to overall emotional wellbeing and a sense of security experienced by students in school.

*The extent to which an individual has robust social ties is likely to have a direct influence on self-concept and sense of belonging, and, in turn, reactions to social stressors. Conversely, the experience of ongoing insecurity and threat has a detrimental effect on emotional wellbeing. An individual’s capacity to deal with adversity is in part dependent on the availability of support in the immediate social environment, as well as the skills he or she has for making appropriate connections at times of stress*. ([65], p. 587)

Connectedness was also seen to help foster better academic learning as the school becomes a more positive environment that students invest in.

##### Eroding boundaries between schools and families

Some multi-level interventions included family components, which were theorised to provide opportunities for students to apply their learning to engage in pro-social behaviours, in particular, conflict resolution at home, and to receive positive reinforcement. Some interventions also aimed to provide training to parents so they might role-model positive behaviours. For example, from the KAT programme:

*Where parents or other community members are actively involved in programmes, they are exposed to the same health-behaviour messages as younger participants and, if they accept those messages, can reinforce them through their own actions, behaviours and attitudes*. ([71], p. 3)

Interpreting this sub-theme in the light of our overarching theme of boundary erosion, these interventions can be viewed as aiming to ensure consistent messaging and erosion of boundaries between learning and behaviour at school and at home.

#### Additional mechanisms to reduce substance use

Although the notion of boundary-erosion offered a useful overarching theme to structure our analysis, some mechanisms that emerged from our synthesis, though they did not contradict the existing thematic structure, could not easily be incorporated within it. As well as cultivating pro-social skills and relationships outlined above, additional key mechanisms of change found in the synthesis for interventions that primarily aimed to reduce substance use involved educating students about substance use and providing training in resistance skills (that is, teaching students to resist peer pressure to use substances).

For example, within Positive Action:

*The program targets the reduction of students’ health-compromising and other negative behaviors (e.g., substance abuse, violence, disciplinary referrals, suspensions), while simultaneously enhancing health-enhancing and other positive behaviors (e.g., honesty, time management) and behavioral attributions directed at the self (…) and social relationships (…), with these leading to improved school-related performance*. ([72], p. 2)

### Hypothesised intervention outcomes

Most of the interventions aimed to reduce aggression, violence or bullying in young people through the mechanisms outlined above, usually in combination. Proximal outcomes commonly highlighted across the interventions leading to reduced violence included the development of pro-social skills, emotional intelligence (broadly understood as self-awareness) and self-management (encompassing managing emotions, emotional regulation, and responding constructively to bullying and conflict). These proximal outcomes were theorised as arising out of the mechanisms described above. Most authors made an overall assumption that anti-social behaviours occur because of a deficit in these social and emotional skills.

For example, the Roots of Empathy programme was reported to have, ‘significantly improved the attitudes, knowledge and social emotional competencies of all participating teachers and children. A decrease in the frequency of bullying and an increase in pro-social behaviours were reported by all participants.’ ([64], pp. 68–69)

Whereas the Positive Action programme was:

*Designed to target the enhancement of positive behaviors and behavioral attributions directed at the self (e.g. self-responsibility, positive self-concept) and social relationships (e.g. conflict resolution, respect, kindness), while simultaneously reducing students’ negative behavioral problems (e.g. substance abuse, disciplinary referrals, suspensions), with these leading to improved school-related performance (e.g. improved attendance, academic achievement)*. ([72], p. 73)

## Discussion

### Summary of the synthesis

The interventions within this review aimed to integrate and thus erode boundaries between academic and health education. They also generally aimed to train teachers to model positive behaviours, reinforce such behaviours among students and enhance their relationships with students. We characterised these as fitting within an overarching theme of boundary erosion, but in doing so clarified that this means transforming relationships from being instrumentally focused on academic learning to being more affective and focused on students’ broader development. This work in the classroom was supported by other components delivered at multiple levels (e.g. the overall school environment and the family), which aimed to ensure that learning and reinforcement of positive behaviours occurred beyond the classroom. We interpreted this mode of delivery as a mechanism that aimed to erode boundaries between what occurred in classrooms and other settings such as the wider school and students’ families. Through these multiple, but theoretically related, mechanisms, interventions were thus intended to promote experiential learning, the practising and reinforcement of positive behaviours and enhanced relationships between teachers and students and between students and their pro-social peers. In turn, it is theorised that these will provide students with various attributes necessary to reduce engagement in substance use and violence as well as to increase academic attainment. These attributes include the knowledge and skills necessary to avoid substance use and prevent conflict, the attitudes and social norms supportive of such behaviours and students’ sense of security within and positive connectedness to their classrooms and schools.

### Strengths and limitations of the review

Overall strengths of the review include having a very comprehensive search strategy, which we believe resulted in maximising our identification of studies relevant to our research questions, as well as a structured approach to the synthesis of theories of change.

Although other existing theory syntheses have focused on some of the same programmes that we have covered here [36, 37], they did exclusively focus on interventions that integrate health and academic learning. Furthermore, through robust systematic review methods, we have achieved more breadth and depth in the analyses of these theories. In contrast to other reviews [40, 41], we chose to focus on theories of change presented in empirical reports or theory reports linked to empirical studies. This decision reflected our (correct) expectation that there would currently be no stand-alone theoretical literature on this newly emergent category of intervention.

Employing meta-ethnographic reciprocal translation allowed us to draw out synergetic ideas from each theory that supported the emergent overarching theme of boundary erosion for health promotion in schools. Authors presented their views of theories that influence the interventions they introduced and, in some instances, explicitly offered theories of change. The use of meta-ethnography allowed us to engage with authors’ descriptions of theories of change as their own interpretation of the world, rather than as secondary constructs based on previous evidence, which was helpful to overcome some of the challenges of applying qualitative synthesis approaches to theory data. Further, the application of multiple methods of qualitative synthesis and the use of an existing theory of schools and health promotion enabled us to develop a deeper analysis of the common mechanisms of action underlying how the interventions in our review are intended to work.

All reports included were appraised for quality and assigned a score based on the criteria indicated in our methods. There was some disagreement between the two reviewers carrying out the quality assessment. As mentioned, there was little guidance available from other studies as to how to resolve challenges encountered in this appraisal. We encountered four main challenges appraising theories introduced through this literature. First, with regards to generalisability, we recognised that many theories of change are programme-specific and thus not intended to be generalisable. Second, we aimed to see if theories were based on existing evidence and/or tested empirically. However, we found that often programme theories drew from multiple existing theories, impeding any simple judgement on the overall evidence base. Third, it was clear that different authors assumed different levels of prior understanding of constructs among their readers, as some provided detailed explanations of constructs and their relationships, while others proceeded as if these were widely understood. It was difficult to decide how to assess the clarity of constructs that were not explained but which we assumed many readers would indeed be familiar with, such as social norms. Finally, we pragmatically used quantitative scores to represent the overall quality of studies and determine which studies we synthesised first despite our tool not having been validated to do this. Overall, as has been noted by authors elsewhere [49], despite carrying out a formal appraisal process, no reports were excluded and all contributed—albeit to different degrees—to the synthesis on the basis of their conceptual strength despite methodological weaknesses.

To achieve a standardised system for appraisal, all reports were treated in the same way, although we were aware that theory was described to different extents depending on each report’s objectives, audience, journal of publication and so forth. Therefore, in some cases, authors’ having more text dedicated to describing theory inevitably led to a higher quality score as well as greater weight being given to their report in our theory synthesis. Furthermore, where there were multiple reports about a single intervention, some of these scored higher in quality assessment, compared to studies in which only one paper reported on theory. Within those assessments, greater weight was given to the studies of higher quality.

These challenges indicate that there continues to be a need to refine criteria. For fields where there is little previous theoretical cohesion, it may be necessary to synthesise every theory in equal measure, particularly since the threat of biasing of syntheses is so much less than is the case with synthesis of empirical evidence.

In terms of applying existing theory to our analysis, our identification of the erosion of boundaries as an overarching theme was informed by our previous reading of the theory of human functioning and school organisation [57]. This theory proposes that young people engage in healthy behaviours when they feel committed to school in terms of learning and as a social community. The theory further suggests that this is most likely to occur in schools that ‘erode boundaries’ (or establish enhanced connections) between, for example, academic and broader learning, teachers and students and schools and their surrounding communities. While not cited by the theoretical literature included in this review, this theory resonates with many of the themes apparent in our initial coding.

Thinking about erosion of boundaries is, in our view, a helpful way of interpreting these theories collectively, but it did require some elaboration of what is meant by boundary erosion in the case of teacher-student relationships (where it meant a transformation of the strength and quality of relationship rather than a negation of all differences and disparities) and student-student relationships (where the emphasis was on building bonds with pro-social students, not all students, and resisting normalised peer behaviour around risk-taking).

The body of literature from which we drew our synthesis raises some additional issues and limitations. In relatively few instances did authors refer to the importance of intervening at specific developmental stages to affect desired changes, so the age at which interventions should be introduced to students did not feature in the mechanisms of change we have proposed. Of note, those interventions intended to reduce substance use focused on adolescents in secondary school, as did those that aimed to reduce conflict and bullying. Interventions like PATHS and Roots of Empathy, which aim to develop social and emotional learning skills and emotional intelligence more broadly, were seen to be of greater use when introduced to younger students. It is unclear whether it is a strength or a weakness of these programmes that some are presented as largely universal across grades, while others are suggested to be age group-specific.

Although some programmes aimed to target both violence and substance abuse, we found in the synthesis that much more explanation and detail was offered about the former, which might affect the salience of our proposed synthesis in some contexts.

Furthermore, there was a general assumption in the literature that risk behaviours arise from deficits in individual knowledge, social and emotional skills and attitudes, albeit within a broader social and institutional context. However, others have argued that behaviours such as violence or substance use emerge as active, symbolically significant practices among young people who are, for complex structural and institutional reasons, disengaged from schools in terms of learning and the social community [56, 73]. Thus, behaviours such as smoking, drug use or violence might occur not merely through deficits but as a result of rationally chosen and socially sanctioned decisions, albeit perhaps within a context of limited choices. The lack of consideration of such mechanisms within programme theories in this review might lead such interventions to fail to address important influences on young people’s substance use and violence. Moreover, despite some mention of the importance of relationships outside the classroom, the classroom environment is overwhelmingly the focus of these interventions, which may fail to address the broader context in which young people develop behaviours.

Finally, most theories did not consider how moderating factors might interact with mechanisms of change as would be the case with theories informed by realist ontologies [74]. Haegerich and Metz [37] extrapolated a summary of moderating factors for some of the same interventions in our synthesis, which included student (e.g. socioeconomic status, ethnicity, gender, intervention dosage) and contextual (e.g. school, family, cultural or policy) characteristics. However, discussions of the role of these factors were consistently underdeveloped in most theories in our synthesis, despite potential implications for programmes’ impacts and the extent to which they might translate between different populations and settings.

### Implications for future research and policy

This review of theoretical literature provides a clearer definition of this novel and potentially important category of intervention, and an insight into the common mechanisms through which diverse real-life examples of integration are intended to work. Centring on the notion of interventions eroding boundaries within and beyond schools will be useful when we come to synthesise empirical evidence from process and outcome evaluations of these same interventions, as we will be able to categorise evaluated interventions by their main intended mechanism of action.

In terms of policy, this intervention type presents great promise as a means of addressing health in busy school timetables, addressing health determinants relating to health knowledge and skills and school engagement and education more generally. Here, we have defined and added clarity to these interventions and their theoretical underpinnings. We are in the process of synthesising process and outcome evaluations of these interventions to determine their feasibility and effectiveness in reducing violence and substance use.

## Conclusion

Here, we have applied Markham and Aveyard’s model of boundary erosion to explain the social and psychological processes that underlie interventions that integrate academic and health education for reduced substance use and/or violence. In particular, eroding boundaries between health and academic education, teachers and students, classrooms and the wider school and schools and families were seen to be the most critical to establishing new frameworks of family, classroom or school organisation that are conducive to promoting both academic and social-emotional outcomes. A better understanding of the theory underlying these approaches should support their further development and evaluation.

## Additional file

Additional file 1: Synthesised theories of change for each individual intervention. (DOCX 1841 kb)

## Abbreviations

4Rs: Reading, Writing, Respect, and Resolution
I-LST: Integrated Life Skills Training
KAT: Kids, Adults Together
LIFT: Linking the Interests of Families and Teachers
LRHC: Learning to Read in a Healing Classroom
PATHS: Promoting Alternative Thinking Strategies
PRISMA: Preferred Reporting Items for Systematic Reviews and Meta-Analyses
SEL: Social and emotional learning
SS: Second Step
