# Supplementary material for: A systematic review and synthesis of theories of change of school-based interventions integrating health and academic education as a novel means of preventing violence and substance use among students
Source: Syst Rev. 2018 Nov 13;7:190. doi: 10.1186/s13643-018-0862-y (PMC6234552; doi:10.1186/s13643-018-0862-y)
Supplement: Supplementary file 1 — Synthesised theories of change for each individual intervention. (DOCX 1841 kb) [file 13643_2018_862_MOESM1_ESM.docx]

**Reading, Writing, Respect, and Resolution (4Rs)**

**
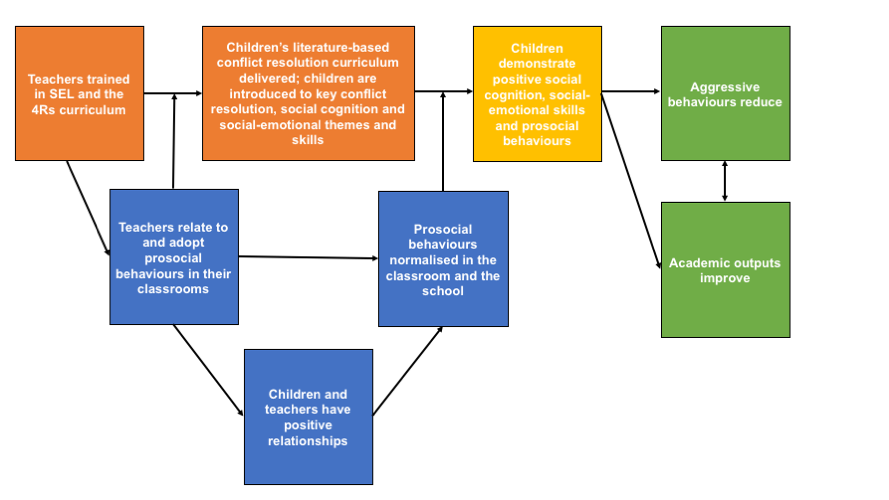
**Through the introduction of a literacy-based conflict resolution curricula in primary school classes, the 4Rs programme aims to develop children’s social-emotional and academic skills for reduced aggressive behaviours and improved academic outcomes. Figure 1 below outlines an overall logic model for the intervention, with each constituent part described more fully in the text that follows.

**Figure 1.** Logic model for the 4Rs intervention

Orange = intervention inputs; yellow = proximal outcomes; green = distal outcomes (impacts); blue = mechanisms expected to facilitate outcomes

**Intervention inputs**

The intervention has two key inputs, the training of teachers, who are expected to deliver the 4Rs curriculum and model its promoted behaviours in their classrooms, and the actual application of the 4Rs literacy-based curriculum. These inputs are the orange boxes in Figure 1. These two inputs were key themes in the theory of change.

**1. Teachers trained in social-emotional learning and the 4Rs curriculum**

Teachers are the key facilitators of the intervention. The initial input of 4Rs is extensive teacher training, not only on the 4Rs curriculum, but also in a more complex introduction of principles related to social-emotional learning (SEL)—generally understood here as the experience, understanding, and management of emotions, both in oneself and in others—and social cognition—how people take in and process information about other people and social situations. Through this training, it is expected that teachers will gain competence in SEL and concepts linked to social cognition, as well as conflict resolution, all of which should hopefully be applied by teachers in their day-to-day interactions with students. The facilitating effects of the teachers’ own beliefs around the themes of the 4Rs curriculum is described more fully under mechanisms of change.

**2. Children’s literature-based conflict resolution curriculum delivered; children are introduced to key conflict resolution, social cognition and social-emotional themes and skills**

Through the literacy-based 4Rs curriculum, children and teachers learn important interpersonal skills such as how to: better recognise ambiguous social cues as non-threatening; be good listeners; show care and empathy; appreciate diversity; value community; find ways to mediate and resolve conflict; and recognise the benefit of prosocial behaviours (e.g. through positive reinforcement and reward; observing other children develop positive relationships with the teacher). Specific skills that 4Rs aims to develop relate to handling anger, listening, curbing aggression, being assertive, and following prosocial negotiation. Additional skills for teachers are around classroom management that fosters a warm and supportive environment that is conducive to prosocial interactions.

The intervention relies on the use of children’s literature as a means of introducing key themes that are expected to increase positive social interactions amongst students. This reading and reflection of literature—considered rigorous literacy instruction—forms the basis of the curriculum used by the intervention alongside coaching given to teachers to reflect and practice their own social and conflict resolution skills.

*“4Rs uses high-quality children’s literature as a springboard for helping students gain skills and understanding in several areas including handling anger, listening, cooperation, assertiveness, and negotiation begins with a comprehensive book reading and discussion, ensuring students understand the primary themes of the story and allowing them to connect the themes to their own lives.”*(1)p.414

The use of literature is intended to allow the 4Rs intervention to be integrated into English classes, or any other classes that might “fit” with the curriculum. This integration is partly for practical reasons, as non-academic curricula have found increasingly less classroom time availed to them.

*“4Rs evolved in response to the tension between the movement to reform education between standards-based accountability with its focus on academic achievement, on the one hand (e.g., the policy and practice zeitgeist promoted by the No Child Left Behind Act of 2001), and social and character development, on the other … From a local perspective, the 4Rs evolved in response to the dramatic reorganization of schools and districts in its local city and the new requirement that city schools adopt a balanced literacy approach to reading that integrates both phonics and whole language approaches to literacy promotion. By integrating the conflict resolution lessons into a curriculum that employs this approach, Morningside Center made it possible for schools to adopt the program into an increasingly tightly scheduled school day by embedding it in the new regularly scheduled balanced literacy block.”*(2)p.156

The authors also refer to the scientific rationale for the integration of academic and social-emotional learning skills based on previous evidence that these are mutually reinforcing. The intervention is expected to be able to add to this evidence through a similar pathway to impact, as discussed later on.

*“The 4Rs Program provides a pedagogical link between the teaching of conflict resolution and the teaching of fundamental academic skills, thereby capitalizing on their mutual influence on successful youth development.”*(2)p.156

*“Our findings to date contribute to the growing evidence that primary prevention strategies designed to address children’s social-emotional as well as academic learning can be effectively integrated and become part of standard practice in classrooms and schools.”*(1) p.417

**Desired outcomes**

There are proximal and distal outcomes theorised in the 4Rs programme (the green boxes in Figure 1). The former relate to the specific skills and competencies that are expected to be built in children, which will ultimately lead to more prosocial behaviours and positive interactions between peers. The latter being the overall impacts of the programme, which is expected to reduce aggressive behaviours—and therefore violence in school—overall, and also to positively impact academic outcomes. Both are key themes in the theory of change. Regarding academic outcomes, as described above, an emerging sub-themes was that there is thought to be a mutually reinforcing aspect of better social cognition and demonstration of SEL and improved academic outcomes, but with its use of literature, this intervention also does directly aim to improve literacy skills as well.

Another sub-theme guiding the theory of change is that the intervention follows the theory of developmental cascades,(3) which suggests that development of skills and knowledge in students and teachers will impact proximal factors related to prosocial behaviours, which will, in turn, impact classroom environments, and ultimately school environments for the normalisation of prosocial behaviours. As such, the intervention aims to target and reinforce effects at multiple levels.

*“In microcontexts (e.g., family, classroom, and school), among the most salient proximal processes are those that involve important relationships. Children experience classrooms through their relationships with their teachers and with their peers, and together children and teachers contribute to a dynamic and enduring set of interactions characterized by regular and consistent patterns. This set of relationships in aggregate constitutes the culture and climate of the classroom environment for all children. Teacher– student relationships are a joint function of the unique characteristics of children (e.g., their social– cognitive attributions and problem-solving style) and teachers (e.g., their social– emotional abilities and experiences of job stress and burnout) and the cultural norms, values, and practices they bring to the relationship and to the classroom. Together these characteristics contribute to the climate of the classroom.”*(2)p.154

*“4Rs is guided by developmental cascades theory which emphasizes an ecological, multi-level lens, focuses attention on change processes in multiple domains, and considers child development in context as dynamic systems.”*(4)p.534

A final sub-theme around outcomes is the timing of the intervention’s introduction. That is, early introduction appears to be key, as it is assumed by the programme developers that be introducing 4Rs in the preschool and elementary school years, it will precede the development of negative and aggressive behaviours in participants. The 4Rs curriculum targets multiple developmental domains in order to reduce current social-cognitive issues in children (e.g. hostile attribution bias) that underlie aggression and conflict but also to develop children’s ‘social competence’(1) to reduce violence and increase prosocial behaviour in the long-term (e.g. by fostering positive peer-networks). Children with less antisocial behaviours or normative ideas about aggression are less likely to rely on strategies of conflict as they grow up.

**Proximal outcomes: children demonstrate positive social cognition, social-emotional skills and prosocial behaviours**

Within the overall theme of proximal outcomes, there are three sub-themes: social cognition, social-emotional skills and prosocial behaviours.

*Social cognition*

Social cognition in the case of 4Rs has multiple targeted domains addressed through the 4Rs curricula, including hostile attribution bias, normalised aggression (which we have made distinct from normalised prosocial beliefs and behaviours, as these are targeted through different aspects of the intervention), and perceived acceptability of aggressive responses, including poor negotiation strategies with peers.

*“The key mental and interpersonal social– cognitive processes directly targeted by 4Rs include hostile attributional bias, namely, the tendency to attribute hostile intent to an ambiguous or prosocial cue; normative beliefs about aggression, or the perceived acceptability of aggressive responses under varying conditions of provocation; aggressive and prosocial fantasies, a form of cognitive script rehearsal about aggressive/antisocial and prosocial interactions and events; and aggressive interpersonal negotiation strategies, or children’s propensity to select aggressive over prosocial responses in hypothetical problem-solving situation.”*(5)p.830

*Social-emotional skills and intelligence*

Development of the social-emotional domain in children is thought to have the strongest link to the development of academic skills. However, it is given less direct focus as a targeted domain within the intervention.

“*The key processes in the social-emotional domain include children’s aggressive and prosocial fantasies, and their levels of affective symptomatology, including symptoms of depression and attention deficit hyperactivity disorder (ADHD).Moreover, because of the fear, anxiety, and sadness caused by environments characterized by high concentrations of children who misperceive social interactions and cues, or who hold beliefs that aggression is a normative response to problem-solving situations, the 4Rs Program is expected to reduce children’s depressive symptomatology and build their capacity to focus attention*.”((4) p.536)

As above, the 4Rs curriculum also relies on the proposed mutually reinforcing nature of the development of both academic and social-emotional skills.

*“Teachers learn how to use high-quality children’s literature as a springboard for helping students gain skills and understanding in the areas of handling anger, listening, assertiveness, cooperation, negotiation, mediation, building community, celebrating differences, and countering bias. By focusing on basic human themes of conflict, feelings, relationships, and community, the 4Rs curriculum adds social and emotional meaning and depth to rigorous literacy instruction.”*(2)p.156

In most instances, these are inter-related.

**Distal outcomes: aggressive behaviours reduce + academic outputs improve**

The overall these of distal outcomes has two sub-themes, which are the impacts the intervention is expected to achieve. Improved prosocial behaviours and better conflict resolution are overall expected to lead to less violence and aggression, and in turn, to also improve academic outcomes. As these are felt to be mutually reinforcing, there exists a cyclical relationship between the two (see logic model).

*“The approach of embedding social-emotional learning and conflict resolution lessons in a balanced literacy delivery strategy, and research tying together the social-emotional and academic domains, support our expectation for longer term effects on behavior and academic achievement.”*(4)p.536

**Mechanisms of change expected to facilitate outcomes**

Under the primary theme of mechanisms of change, there are three sub-themes, which are the three key pathways to impact in the intervention (the blue boxes in Figure 1), which centre around the role of the teacher and their modelling of the intervention’s principles in their own classroom, which is expected to contribute to an overall normalisation of prosocial behaviours.

**1. Teachers relate to and adopt prosocial behaviours in their classrooms**

Foremost in the normalization of prosocial behaviour is the role of the teacher, whose personal beliefs about the intervention content are central to the quality of instruction. As above, teachers’ ability to model prosocial behaviours is felt to be fundamental to the normalisation of a classroom environment that discourages aggression.

*“The program’s theory of change emphasizes the role of introducing teachers to a set of social–emotional learning skills and concepts and then supporting them in the use of these skills and concepts in their everyday interactions in the school with one another, with school administrators, and with the children in their classrooms through the consistent teaching of lessons from the 4Rs curriculum. At its core, the program’s theory of change involves helping teachers more deeply assimilate, find utility in, and become skilled at practicing the concepts of the 4Rs Program in their own lives and teaching them in their classroom through the consistent delivery of lessons from the 4Rs curriculum and the provision of greater social– emotional learning opportunities in which students can practice the component skills and be supported in applying them in real-life situations.”*(2)p.157

**2.** **Children and teachers have positive relationships**

Related to the role of the teacher is the development of positive relationships in the classroom system. Through positive teacher-children relationships (‘teacher-child’ as well as ‘teacher-children’ as a whole), children can model their own behaviour on that of their teachers. Peer relationships are improved also because children model their own behaviour on those of other children that they see have closer and more positive relationships with the teacher. Through these multiple relational processes, the classroom system as a whole becomes a more caring environment with reduced levels of aggression. This fosters better academic performance in children and enables the teacher to focus on academic topics by reducing the work needed to control classroom behaviour. Furthermore, school administrators and other members of staff also receive training through the programme, so that they can be aware of, endorse and support the teacher in practicing the conflict resolution skills promoted by the intervention.

*“But it is not merely the practice of good listening skills by the teacher or any given student that is important; it is how the use of these skills reflects a set of transactional social processes enabling teachers and students to develop closer, more intimate relationships. Increases in the quality of the relationships among teachers and students in turn facilitates future positive communications by fostering a more responsive classroom overall.”*(6)p.4

**3. Prosocial behaviours normalised in the classroom and the school**

Key to the intervention is the normalisation of prosocial behaviours in the classroom. These are expected to be modelled by teachers, within their interactions with students, and also by students in their interactions with teachers and peers. These prosocial interactions are therefore thought to develop positive relationships within a classroom, which encourage further prosocial engagement.

*“When teachers embrace and practice the program’s principles and implementation strategies, they establish a set of expectations and norms for behaviors in their classrooms, and children begin using those skills and behaviors. Teachers who practice good listening skills (e.g., direct eye contact, paraphrasing, acknowledging comprehension) during interactions with their students and other adults, and who can teach these skills and provide real-life, real time examples of how they are effective, increase the likelihood that their students will employ them in their own interactions.”*(2)p.157

**Moderating Factors**

Overall, little mention is made of factors that might affect the mechanisms in the theory of change or influence the effectiveness of the intervention. As such, no transferrable themes around these emerged, although moderating factors briefly mentioned once are related to: exposure (for example attendance in school; exposure to the intervention or the teacher); and poverty-related risks (poorer children appear to benefit more from the intervention than less poor children).

**Assumptions**

Underlying the theory of change, implementers have acknowledged a number of assumptions. Thus, under the theme of assumptions, sub-themes of expectations around development of desired SEL competencies through normalisation of desired behaviours, reinforcement of positive behaviours, and also through observation of positive behaviours in others emerged.

*“Like most school-based SEL programs, the 4Rs is grounded in several theoretical assumptions about students’ social and emotional development. First, students are expected to develop social-emotional competencies by reacting to norms within the school environment that are changed to emphasize more positive social behaviors. Second, they develop social-emotional competencies through environmental consequences of their actions, particularly reinforcement for positive behaviors (e.g., praise or rewards for helping others). Third, they develop these competencies through observing others who model the desired skills and characteristics, as well as through practicing them in social contexts.”*(7)p.8

**References**

1. Aber L, Brown JL, Jones SM, Berg J, Torrente C. School-based strategies to prevent violence, trauma, and psychopathology: The challenges of going to scale. Development and Psychopathology. 2011;23(02):411-21.

2. Brown JL, Jones SM, LaRusso MD, Aber JL. Improving classroom quality: Teacher influences and experimental impacts of the 4rs program. Journal of educational psychology. 2010;102(1):153.

3. Masten AS, Cicchetti D. Developmental cascades. Development and psychopathology. 2010;22(03):491-5.

4. Jones SM, Brown JL, Lawrence Aber J. Two‐year impacts of a universal school‐based social‐emotional and literacy intervention: An experiment in translational developmental research. Child development. 2011;82(2):533-54.

5. Jones SM, Brown JL, Hoglund WL, Aber JL. A school-randomized clinical trial of an integrated social–emotional learning and literacy intervention: Impacts after 1 school year. Journal of Consulting and Clinical Psychology. 2010;78(6):829.

6. Jones SM, Brown JL, Aber JL. Classroom settings as targets of intervention and research. 2008.

7. Sung J. Teachers' perceptions and experiences of the implementation of a social-emotional learning program in an inner-city public elementary school. New York: Columbia University; 2015.

**Bullying Literature Project**


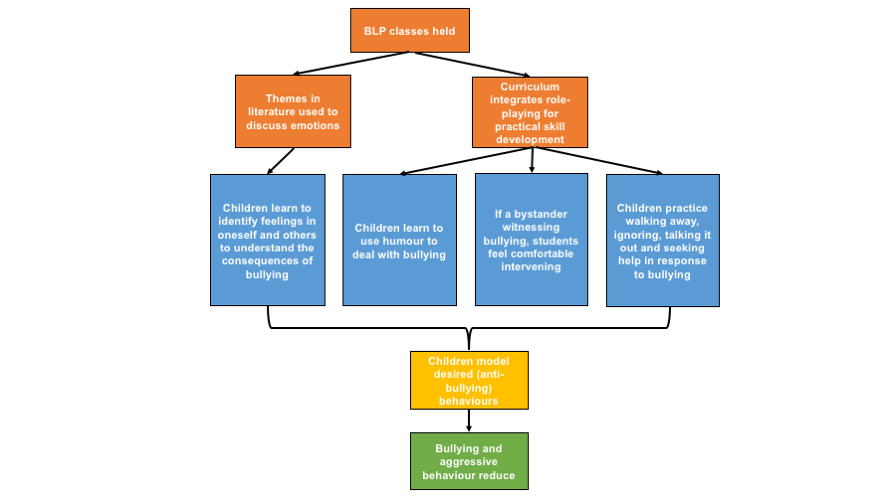
The Bullying Literature Project (BLP) integrates themes related to bullying into the children’s literature used within a standard English curriculum. BLP also provides an opportunity for children to role-model practical skills to address or avoid bullying. Ultimately, the programme aims to ensure that children respond constructively to bullying, with reduced demonstration of aggressive and bullying behaviours.(1–3) Figure 1 highlights the key components of the programme through a logic model of its predicted pathways to outcomes.

**Figure 1**. Logic model of the Bullying Literature Project. Orange = intervention inputs; yellow = proximal outcome; green = distal outcome (impacts); blue = mechanisms expected to facilitate outcomes

**Inputs**

The key theme of the intervention is that literature can be used both to discuss emotions and as a platform from which practical skills to address bullying—both as a target and a bystander—can be built. Thus, the actual BLP curriculum is the primary input of the intervention. This intervention is delivered by external facilitators. As such, there are no inputs around teacher or staff training.

**Integration**

The author highlights the cost-effectiveness of addressing bullying by capitalising on pre-existing curriculum. Unlike in other interventions, authors do not refer to the mutual benefit of both academic and social-emotional (or on this case, anti-bullying) learning.

*“The Bullying Literature Project (BLP), a brief class-wide bullying intervention designed to integrate into the existing curriculum and provide educators a cost-effective way to target bullying on campus.”*(1)p.iv

*“One possible methodology for integrating academics and a bullying intervention program is through bibliotherapy.”*(1)p.7

**Mechanisms of change**

There are four mechanisms through which the BLP curriculum is expected to achieve the desired outcomes of the programme. Of these, one key theme emerges around the importance of modelling of desired behaviours and practices, which is facilitated through the role-playing components of the curriculum. The authors suggest that this aspect of the BLP programme is what distinguishes it from other bibliotherapy-based school anti-bullying programmes. In particular, learning to walk away, ignore, talk it out or seek help are strategies emphasised to address bullying. Additional mechanisms that emerged as sub-themes include: identifying feelings in oneself and others; using humour to deal with bullying; and intervening in bullying rather than being a bystander.

*“The discussion questions* [in the BLP curriculum] *were designed to highlight key points in the story, help students identify feelings in themselves and others, promote positive bystander behavior, change negative attitudes regarding bullying, and emphasize effective ways to handle bullying and peer conflict.”*(1)p.17

This programme differs from many of the other anti-bullying or aggression-reducing interventions that we have explored in that the social-emotional learning aspects seem to be of less importance, and understanding emotions and developing empathy are introduced, but given considerably less emphasis than in other interventions included in this review.

**Outputs**

The author is not explicit about the desired outcomes of the intervention within a discussion of programme theory. However, extrapolating from the outcomes of the overall study she conducted, bullying behaviours and responses to bullying were explored, both of which are expected to be improved through this programme.

**Assumptions**

The author refers to the importance of an anti-bullying curriculum that is integrated into the regular curriculum. However, this intervention relied on eternal facilitators to deliver the programme. Thus, it is an assumption that something not provided by regular teachers could be fully integrated into the curriculum, both from the perspective of a lack of connection with the English curriculum currently being taught, and also considering sustainability and cost-effectiveness. The author argues the latter as a justification for integration.

**References**

1. Couch L. The Bullying Literature Project: An Evaluation of a Class-Wide Bullying Intervention Program. 2015.

2. Wang C, Couch L, Rodriguez GR, Lee C. The Bullying Literature Project: using children’s literature to promote prosocial behavior and social-emotional outcomes among elementary school students. Contemp Educ Psychol. 2015;19(4):320-329.

3. Wang C, Goldberg T. Using children's literatrue to decrease moral disengagement and victimization among elementary school students. Psychology in Schools. 2017;54:918–931.

**DRACON**


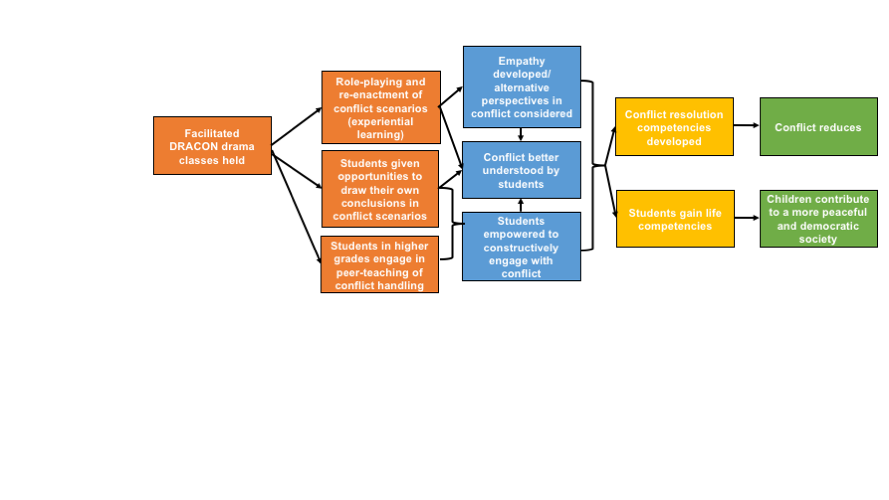
DRACON is an integrated school-based programme that uses educational drama to teach conflict management to students, emphasising the importance of experiential learning. Figure 1 below highlights a proposed logic model for the programme.

**Figure 1.** Proposed logic model for the DRACON programme

Orange = intervention inputs; yellow = proximal outcomes; green = distal outcomes (impacts); blue = mechanisms expected to facilitate outcomes

**Inputs**

The sole input described by authors are drama sessions, facilitated by trained DRACON instructors. These sessions integrate practical conflict management into drama classes. The DRACON programme sees this integration as ideal because drama is considered, by the authors, as a close representation of human experience and experiential learning, which is seen as central to conflict management and resolution.

*“The basic idea of this project has been to merge two academic and practical fields, which often have been separated—educational drama and conflict management. Conflict management on a deeper level requires processing personal experiences of conflict and that is precisely what can happen through drama work. The fundamental hypothesis in this project is that drama can be an effective way to learn conflict handling.”*(1)p.2

Additionally, as described below, in drama sessions, students are also provided with empowering opportunities through self-directed navigation of conflict scenarios and engagement in peer learning.

**Mechanisms of change**

Two key themes emerge as mechanisms of change for this intervention: experiential learning and student empowerment.

In terms of experiential learning, the authors cite the work of Kolb (1984) who defined this as a “*process whereby knowledge is created through the transformation of experience.*”(Kolb, 1984, p. 38, cited in (1) p.6)

The authors discuss the centrality of experiential learning, seen as a cycle that involves the whole person (rather than just one’s intellect) in the process of learning, as a mechanism to teach conflict management and competencies to students.

*“We believe that experiential learning is one of the best ways for students to learn conflict handling skills (…) In conflict situations and in learning about conflicts it is not only logical thoughts that are involved; the whole person is engaged with senses and emotions. Therefore, in many situations pedagogical methods that profoundly engage the whole person are needed for more efficient learning.”*(1)p.6

Further, development of empathy can be facilitated through the experiential learning that is, in particular, offered through drama lessons. Role-playing or re-enactment in drama provide students with ‘distance’ from the conflict that enables them to not only embrace alternative perspectives, but also to explore outcomes of alternative responses to a conflict situation.

“*They* [students] *can also explore how the conflict looks to other participants, for instance*

*how the opponents experience the conflict, through playing the role of the opponent.”*(1)p.3

*“Through re-enactment or role-play the participants access a more meaningful experience of the conflict, including thoughts, feelings and body experiences. On the other hand, the participants can distance themselves from these experiences through the fictional character of role-playing. In this way they can explore alternative actions and their consequences.”*(1)p.3

Regarding empowerment, Malm (2007) proposes that a way of empowering students is through assigning them a role in the process of learning from the intervention.

*“It is not enough to teach conflict handling skills to our students. We want them to take over the responsibility for the teaching and learning processes as well as for the handling of actual conflicts among themselves. Empowerment for learning takes the form of developing drama programmes in which the students are not told how to behave in conflicts but are provided with conflict situations from which they can learn and draw conclusions themselves.”*(1)p.6

In some cases, the students can be empowered through becoming peer-teachers and mediators in situations of conflict:

*“Empowerment for teaching takes the form of peer teaching, where higher-grade students who have participated in the DRACON programme teach students in the lower grades about conflict handling. Empowerment for conflict handling takes the form of building self-help capacity in all students as well as intervention capacity in some of them. By intervention we mean basically some form of peer mediation.”*(1)p.6

A sub-theme around the understanding of conflict emerged. A goal of the curriculum—as well as a product of both indicated mechanisms of change—is that conflict will be better understood by students. This understanding is of the root causes of conflict and how it affects people experiencing conflict. Improved understanding of conflict is felt to be a necessary precursor to developing conflict resolution skills.

**Outcomes**

The DRACON intervention is expected to achieve a number of outcomes through the process outlined above. As more proximal outcomes, students are expected to develop conflict resolution competencies and ‘life competencies’. What these ‘life competencies’ are is not fully described. The programme is thus also seen to develop a more ‘harmonious school environment’ through a reduction in conflict.

As more distal outcomes, students’ life competencies are seen as a way to raise empathic and responsible young people who will go on to contribute to a peaceful and democratic society overall.

*“By developing and researching drama programmes for constructive conflict handling, this project is aimed at contributing to the creation of a more harmonious school environment, and to provide the students with life competences and social values that will have an impact on the future of society and ultimately contribute to the building of a democratic and peaceful culture.”*(1)p.4

**Assumptions**

The overall programme theory has five key assumptions.

*“Our theoretical thinking and our practical work can be summarised in five basic assumptions: (1) It is possible to improve conflict literacy handling through drama. (2) School is a strategic arena for learning conflict handling and literacy skills. (3) Early adolescence is a critical period for learning conflict resolution strategies, as there is a high frequency of conflicts. (4) Ways of handling conflicts are culturally conditioned. (5) Empowerment of students is needed in order to build up self-help as well as intervention capacities.”*(1)p.3

Additionally, a fundamental underlying assumption in the DRACON literature is that conflict-related competencies, including empathy, can only be taught through practice. This drives their reliance on drama exercises as ways to re-enact experiences and role-play. The two extracts below show the overarching impact of this assumption on the programme theory.

*“Practical skills or conflict competence cannot be taught only in theory, just as conflict cannot be studied on a purely theoretical level. Empathy, active listening and appropriate communication are life competencies that one can only achieve through practical training, as they involve not only mental intelligence but also emotional and physical intelligence.”*(1)p.2

*“We believe in the importance of learning conflict competence through one’s own experience (experiential learning) rather than through books or lectures.”*(1)p.3

Specifically, regarding their educational drama approach, the authors also suggest that, *“the learning situation needs to be structured through a trained facilitator and that is exactly what happens in educational drama.”*(1)p.3 It is not clear whether the authors are implying that these facilitators need to be external DRACON-trained facilitators or simply drama teachers who have been trained in a conflict resolution approach to experiential learning in drama.

These assumptions are not fully explained or supported with theoretical or empirical evidence from other authors or studies.

**References**

1. Malm B, Löfgren H. Empowering students to handle conflicts through the use of drama. Journal of Peace Education. 2007;4(1):1-20.

**English Classes**

This intervention introduces health topics to students through finding compatible points within English curricula where these topics can be addressed, while still achieving curricular goals. Although not explicit, it appears that, through this infusion of health into repeated English classes, students will internalise health messages and will have better health behaviours, including reduced substance use.

Figure 1 below is a suggested logic model for this programme. This intervention does not offer a clear theory of change for how it is expected to function. As with some of the other interventions included in this synthesis, what we have provided is an extrapolation based on whatever detail the authors have provided.


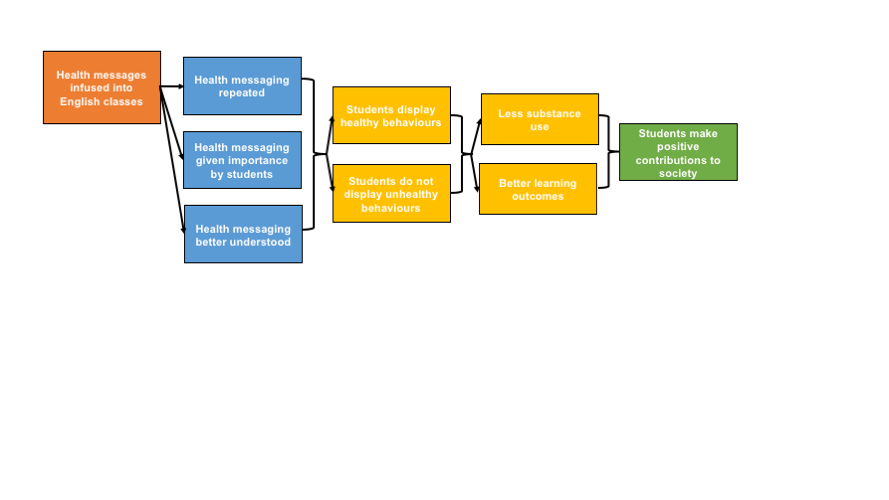


**Figure 1.** Proposed logic model for the integrated health and English classes programme

Orange = intervention inputs; yellow = proximal outcomes; green = distal outcomes (impacts); blue = mechanisms expected to facilitate outcomes

**Inputs**

The only input is the integrated English curriculum itself. Integration here is achieved by infusing health topics into the English curriculum where appropriate.

The only theme that emerges from this study is derived from learning theory. The authors suggest that, *“students often forget material that is not repeated, and they sometimes do not pay attention to (and therefore do not learn) lessons presented in classes they perceive as relatively unimportant. Conversely, it can be inferred that the repetition of (health) concepts, especially in varied settings (in this case, different classes), increases the likelihood that they will be retained. Also, integrated subject matter helps students better understand ideas and solutions to problems that span several disciplines.”*(1)p.S-37 Thus, through repetition of material and positioning it within a context in which students are more likely to ascribe it with importance, health messaging is more likely to be retained.

**Mechanisms of change**

As above, the authors imply that students will be more receptive to health messaging through the English classes platform. Further, as a result, they will be more likely to demonstrate the healthy behaviours encouraged and avoid the unhealthy behaviours that are discouraged, such as substance use. From this, the authors imply that students will, overall, be healthier, and therefore better able to learn.

*“It was hoped that these efforts would increase students’ awareness and practice of healthy behaviors. Students’ health can impact profoundly on their ability to learn. Unhealthy students and those who practice high-risk behaviors are more likely than others to fail in school or at least to leave school without the skills necessary to become productive contributors to society.”*(1)p.S-36

**Outcomes**

The key proximal outcomes of this programme are suggested to be increased healthy behaviours and decreased unhealthy behaviours. Distal outcomes related to these are therefore reduced substance use, better learning outcomes and, ultimately, productive contributions to society.

**Assumptions**

The authors presume that repetitive health messaging will somehow lead necessarily to healthier practices and reduced unhealthy practices with little justification of this assertion. Further, the link to positive learning outcomes and later to productive contributions to society are not well grounded in any literature.

**References**

1. Holcomb JD, Denk JP. An Interdisciplinary English/Health Connection: Promoting Health Awareness and Healthy Behaviors. Journal of Health Education. 1993;24(sup1):S-36-S-41.

**Hashish and Marijuana**

Hashish and Marijuana is a substance use reduction/avoidance curriculum that teaches the chemical aspects of the substances during chemistry classes and constructive decision-making during participatory components outside of chemistry classes.


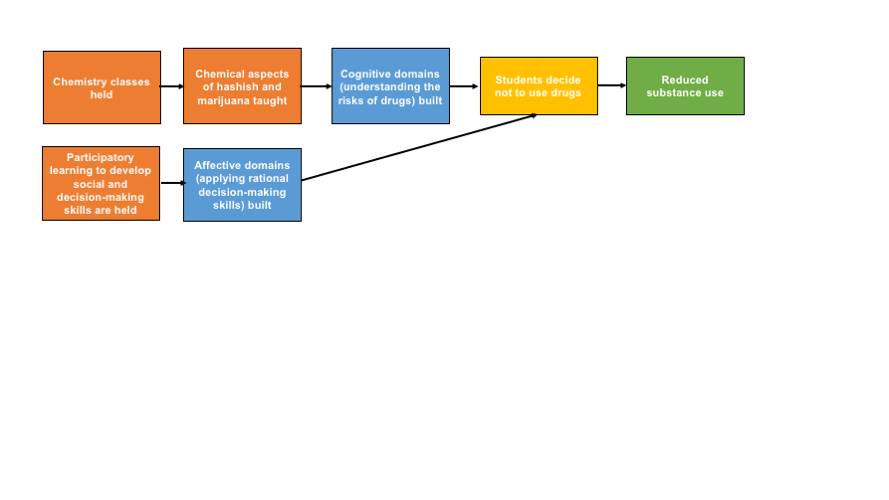
**Figure 1.** Proposed logic model for the Hashish and Marijuana programme

Orange = intervention inputs; yellow = proximal outcomes; green = distal outcomes (impacts); blue = mechanisms expected to facilitate outcomes; red = theoretical positioning

**Inputs**

The intervention’s core input is chemistry classes, which teach the chemical aspects of hashish and marijuana. In order to encourage behaviour change, there are also group work, class discussions, independent field trips, and any other curricular additions instructors may choose to use to develop social- and decision-making skills.

Integration of health and academic curricula are achieved through the use of chemistry classes to teach chemical aspects of hashish and marijuana that are intended to dissuade students from using them, while building aptitude in chemistry as well.

**Mechanisms of change**

A central theme—perhaps the only theme emerging from the description of theory for this intervention—is that, both cognitive and affective domains are built through the intervention. The former developed through chemistry classes and the latter through the more participatory and interactive curricular additions. As such, it is felt that knowledge will be acquired alongside desired behaviour change as students apply rational value judgments to their decision-making.

*“There is a continuous effort to simultaneously integrate the cognitive and the affective domains throughout the program...active participation and personal involvement on the part of the students...are specifically built into the program.”*(1)p.40

Coupled with the curriculum around the chemical and biological aspects of drugs, it is therefore postulated that students will have a *“[weakened] desire for personal experiences of using drugs...to help certain students in finding 'real' solutions to some of their real problems*.”(1)p.40

**Outcomes**

The main outcome for this intervention is reduced substance use, specifically hashish and marijuana among students receiving the programme.

**References**

1. Zoller U, Weiss S. “Hashish and Marijuana”—An Innovative, Interdisciplinary Drug Education Curricular Program for High Schools. Journal of Drug Education. 1981;11(1):37-46.

**Infused Life Skills Training (I-LST)**

The Infused Life Skills Training programme (I-LST) is an intervention that hinges on the integration of health education into the regular curriculum. Lessons are designed to be relevant to students, interactive and with opportunities for repetition and reinforcement across subject areas. Provision of health education through this platform is therefore felt to optimise learning, leading students to internalise health messages and exhibit positive health behaviours. Figure 1 below outlines a suggested logic model for this programme.

**
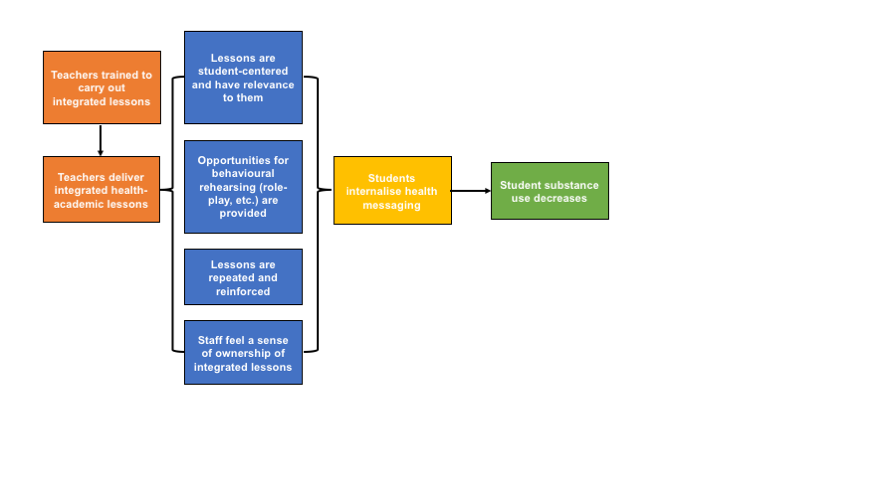
**

**Figure 1.** Proposed logic model for the Infused Life Skills Training programme

Orange = intervention inputs; yellow = proximal outcomes; green = distal outcomes (impacts); blue = mechanisms expected to facilitate outcomes

**Theoretical positioning**

The authors do not really indicate a theoretical positioning, except suggesting that the structure of the lessons should follow Hunter’s integration model, “Instructional Theory into Practice”.(1)This model indicates that lessons must simultaneously address academic and life skills objectives.

**Inputs**

There are two basic inputs within this intervention: the training of teachers, which included components of brainstorming around possible areas of integration, and the actual delivery of integrated curricula.

**Mechanisms of change**

The authors do not explicitly state potential mechanisms of change, so what is presented here is an extrapolation. The primary mechanism of change appears to be integration of messaging around health topics (substance use in particular) into academic learning in an engaging way. In particular, “*teachers were also encouraged to be certain that the activities were developmentally appropriate and that the lessons included facilitation, coaching and behavioral rehearsal of skills as well as student-centered learning strategies*.”(2)p.222 This format of lesson was felt to be the most effective in conveying health messaging.

“*A well-implemented, infused program that includes faculty from various academic areas can send consistent prevention messages to students through multiple channels and help as many faculty as possible to develop a sense of ownership and commitment to the prevention effort. The focus of an infused curriculum is to enhance the regular subject area by increasing student knowledge, understanding, and motivation while building prevention-related personal competencies*.”(2)p.221

Health lessons were integrated into a broad range of core academic subjects including science, English, social studies, geography, math, art, music and physical education. As needed, designated health lessons topped up lessons that teachers were unable to integrate. Teachers themselves were responsible for identifying potential links between the two types of curricula.

**Outputs**

The overall output of the intervention is improved health, but from the perspective of this review, the aspect of health of our interest is reduced substance use.

**Assumptions**

There are two key assumptions behind this intervention, all of which are focussed around the potential of integrated health and academic lessons, and could therefore be thought to also underlie the intervention’s mechanisms of change:

1. “*Such an integrated approach to prevention also increases the probability the components will become an integral part of the ongoing permanent curriculum…[representing] a mode of drug prevention programming that is potentially easier for a school to adopt and maintain; therefore, it may be less of a burden on both the already limited time for health education and for time away from other content areas.*”(2)p.221 Therefore, this format of delivery may guarantee a place for health education in schools with constrained timetables.
2. “*Optimal learning occurs when information is embedded in meaningful contexts, applications and multiple representations are provided, and there are opportunities for learners to generate personally relevant questions*”,(2)p.220 thus making an integrated learning approach a more effective one.

**References**

1. Hunter M. Instructional theory into practice. Virginia: Polythecnic Institute. 1984.

2. Bechtel LJ, Vicary JR, Swisher JD, Smith EA, Hopkins AM, Henry KL, et al. An interdisciplinary approach for the integration and diffusion of substance abuse prevention programs. American Journal of Health Education. 2006;37(4):219-25.

**Kids, Adults Together (KAT)**

The Kids, Adults Together (KAT) programme is intended to reduce alcohol use in young people. It has an anti-alcohol curriculum integrated into other subject areas to mutually develop academic skills as well as practical skills that young people will need to avoid and decline the use of alcohol. Additionally, there is a parent evening, in which students present to their parents what they have learned about alcohol. A DVD is made available to students and their parents to watch and discuss at home. Beyond the knowledge and practical skills that the classroom curriculum aims to develop, the crux of the programme’s intended effectiveness lies in the development of prosocial bonds between students and adults, especially their parents. These positive bonds are felt to decrease the likelihood of negative behaviours and provide positive reinforcement of positive behaviours. Further, parents who participate in the programme are expected to reinforce and role-model its messages at home, further decreasing the likelihood of their children using alcohol if a norm against using alcohol has been established in their household. Figure 1 outlines a proposed logic model for this programme.


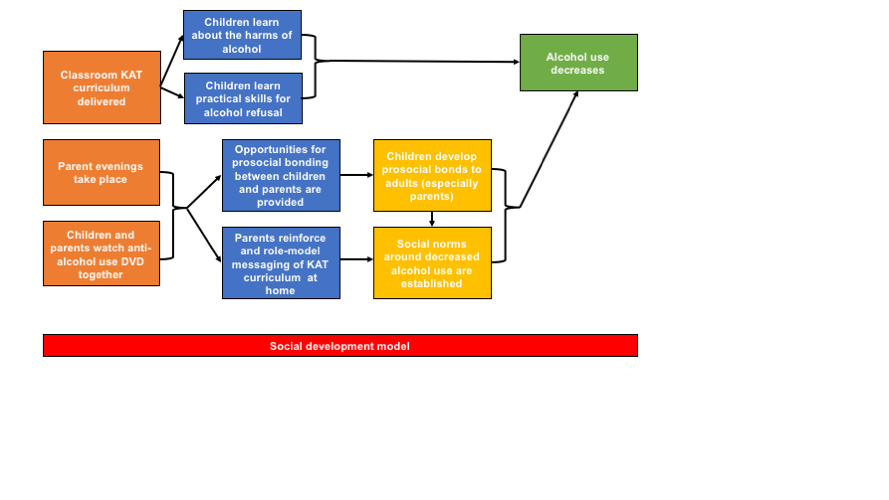


**Figure 1.** Proposed logic model for the Kids, Adults Together programme

Orange = intervention inputs; yellow = proximal outcomes; green = distal outcomes (impacts); blue = mechanisms expected to facilitate outcomes; red = underlying theoretical position

**Theoretical positioning**

The underlying theory around which the intervention is based is the social influence or social development model.(1) This model, “*children can be ‘ inoculated’  against social pressure to adopt undesirable behaviours such as drug and alcohol use” primarily through “through interactions with others, resulting in the formation of attachments which, if strong, can have a lasting effect on behaviour through supporting the acquisition of skills and influencing norms and values*.”(2)p.2 The authors also use this theory as the basis for inclusion of parents in the intervention as highlighted in the inputs below, suggesting that:

“*Attachment to others who offer opportunities for and reward prosocial behaviour is a protective factor against antisocial behaviour. Thus, involvement of both parents and children in interventions may increase the quality and frequency of parent– child interactions*.”(2)p.xxi

**Inputs**

There are three components to this intervention: 1. a classroom component, in which students learn material (around alcohol use) and prepare presentations for a parent evening; 2. a parent evening in which students demonstrate what they have learned about alcohol to their parents so that they can discuss together; and 3. a follow-up DVD for parents and children to watch together.

**Integration**

The classroom component integrates lessons around the effects and consequences of alcohol into various classes. For example, in English classes, in addition to learning about alcohol, students also learned how to collect and present data; in art classes, learning was tied into development of skills in arts and design, such as creating anti-alcohol posters and awareness material; and in drama classes, students role-played the effects of alcohol on families and the broader community.

**Mechanisms of change**

There are a number of mechanisms through which the KAT intervention is expected to achieve its desired outcomes in students. Regarding the curriculum itself, the interactive teaching styles are felt to be more effective than didactic approaches, leading students to engage more with messaging. Alongside this interaction is the provision of opportunities to role-model desired behaviours (or to role-model negative behaviours to explore their consequences), practice refusal skills and problem-solve.

Having programme components at different levels is expected to be most impactful at the family level. Engagement of families is expected to: *“[create] opportunities for prosocial interaction between and within families; [strengthen] the necessary skills which parents and young people need to communicate about alcohol-related issues; and [encourage] parents to reward and reinforce prosocial behaviour and attitudes in relation to alcohol.”* (2)p.5 Therefore, the creation of prosocial bonds and attachment between students and adults—especially their parents—is felt to be critical to this intervention’s success.

Beyond rewarding positive behaviours, involvement of parents is expected to provide opportunities for children to have any messaging from the programme reinforced and role-modelled at home due to their direct engagement with and internalisation of the KAT curriculum during parent evenings and through watching the DVDs. By seeing these behaviours at home, it is expected that a norm around alcohol use will be established, which children will adhere to due to their desire to maintain prosocial bonds.

*“Where parents or other community members are actively involved in programmes, they are exposed to the same health-behaviour messages as younger participants and, if they accept those messages, can reinforce them through their own actions, behaviours and attitudes.”*(2)p.3

**Outputs**

Overall, children are expected to use less alcohol as a result of this programme.

**Assumptions**

One assumption is simply that bonding to adults (especially parents) and having reinforcement of less or no alcohol use at home will be more effective in mitigating alcohol use than the use of alcohol by and with peers.

A second is that, “*according to the social influence model, interactive teaching and learning methods (e.g. role play) might increase programme effectiveness by providing opportunities for communication and social interaction and enhancing young people’ s critical awareness of social norms and pressures around substance use.”* (2)p.2 Thus, the assumption is that young people may have lacked this awareness, when in fact, the negative consequences of alcohol may already be well-known.

**References**

1. Hawkins JD, Weis JG. The social development model: An integrated approach to delinquency prevention. Journal of Primary Prevention. 1985;6(2):73-97.

2. Segrott J, Rothwell H, Hewitt G, Playle R, Huang C, Murphy S, et al. Preventing alcohol misuse in young people: an exploratory cluster randomised controlled trial of the Kids, Adults Together (KAT) programme. Public Health Research. 2015;3(15):1-188.

**Learning to Read in a Healing Classroom**

The intervention aims to improve the academic and psychosocial outcomes of students by improving teacher motivation and well-being, and modifying the social and pedagogical interactions between students and teachers.


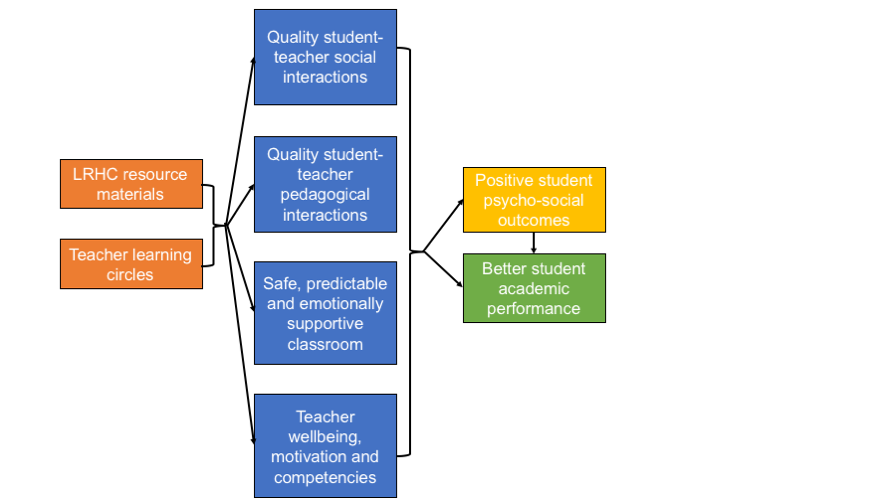


**Figure 1.** Proposed logic model for the LRHC programme

Orange = intervention inputs; yellow = proximal outcomes; green = distal outcomes (impacts)

**Intervention inputs**

The intervention consisted of two key components: integrated teacher resource materials and collaborative school-based Teacher Learning Circles.(1,2)

The materials aim to create student-centered, safe, predictable, and emotionally supportive learning environments, with scaffolded pedagogical content and practices to improve French reading instruction. The materials focus on addressing students by name, avoiding corporal punishment and using positive discipline strategies, establishing a regular classroom routine, encouraging students to express themselves, using small-group learning activities to encourage interaction, connecting academic content to students’ lives, asking open-ended questions, and promoting student participation. These should provide teachers with pedagogical content knowledge and skills, allowing them to respond to children’s social and emotional needs, and improve the quality of the learning environment.

Teacher Learning Circles gave teachers the opportunity to exchange information,

collaborate, solve problems, and support and motivate one another. They aimed to promote collegiality and increased sense of professional self-worth. Participation aimed to increase teachers’ motivation and improve the social and pedagogical processes in classrooms and thus engender improvements in students’ well-being and academic performance.

**Mechanisms**

The intervention inputs aim to establish a safe, orderly, and emotionally supportive classroom environment leading to improved student-teacher interactions. The quality of social and pedagogical interactions between teachers and students are theorised as key predictors of academic and psychosocial outcomes.

**Outcomes**

The theory is informed by self-determination theory which suggests that student-teacher and student-student interactions can meet students’ needs for autonomy, safety, connectedness, and competency. Meeting these needs are viewed as fundamental for mental health and can also motivate students to pursue positive social and academic goals.(3-5)

**References**

1. Aber JL, Tubbs C, Torrente C, Halpin PF, Johnston B, Starkey L, Shivshanker A, Annan J, Seidman E, Wolf S: Promoting children's learning and development in conflict-affected countries: Testing change process in the Democratic Republic of the Congo. *Development and Psychopathology.* 2017, 29(1):53-67.
2. Torrente 2015: Torrente C, Johnston B, Stakrey L., Seigman E, Shivshanker J, Weisenhorn N, Annan J, Aber JL. Improving the quality of school interactions and student well-being: impacts of one year of a school-based program in the Democratic Republic of the Congo. Journal on Education in Emergencies. 2015;1(1):48-91.
3. Deci EL, Ryan RM. The ‘What’ and ‘Why’ of Goal Pursuits: Human Needs and the Self-Determination of Behavior. *Psychological Inquiry.* 2000; 11 (4): 227–68.
4. Deci EL, Vallerand RJ, Pelletier LG, Ryan RM. Motivation and Education: The Self-Determination Perspective. *Educational Psychologist*. 1991; 26 (3-4): 325–46.
5. Hughes JN, Luo W, Kwok O, Loyd LK. Teacher-Student Support, Effortful Engagement, and Achievement: A 3-Year Longitudinal Study. *Journal of Educational Psychology.* 2008; 100 (1): 1.

**LIFT**

**
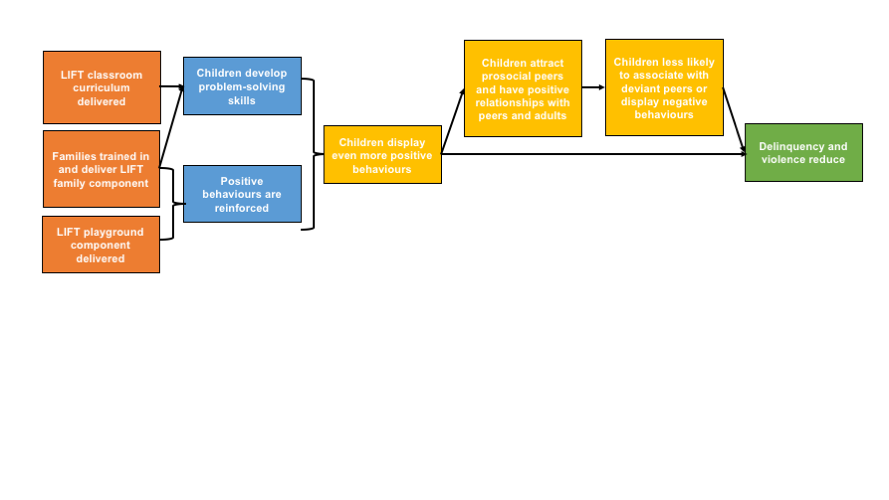
**The Linking the Interests of Families and Teachers (LIFT) intervention has three components: classroom and playground components in addition to a parent training programme. The overall aim of LIFT is to reduce violence and delinquency in children. The overarching theme guiding LIFT’s theoretical model is that children will not engage in antisocial behaviours that predict later violence and delinquency if, early on, good, pro-social behaviours are positively reinforced. Thus, through each component, there are aspects of problem-solving skill development in order to train children to use good behaviours and positive reinforcement of these behaviours. Figure 1 suggests a logic model for the programme. Of note, however, is that the authors were not explicit in a theory linking inputs, mechanisms of change, and outputs to their programme as we have done here. Thus, all of the relationships expressed in Figure 1 are extrapolated on the basis of whatever information has been provided.

**Figure 1.** Proposed logic model for the LIFT programme

Orange = intervention inputs; yellow = proximal outcomes; green = distal outcomes (impacts); blue = mechanisms expected to facilitate outcomes

**Theoretical positioning**

The authors suggest that the LIFT programme is based on coercion theory. However, that appears contradictory, as coercion theory emphasises the role of negative reinforcement in the development of a child, whereas LIFT emphasises the role of positive reinforcement.

*“In coercion theory, the key mechanism hypothesized to drive the development of child problem behaviors is negative reinforcement. Negative reinforcement is the association of certain behaviors with the termination or delay of aversive situations, such as a person hitting the snooze button when his or her alarm rings in the morning. In contrast, the more familiar positive reinforcement or reward paradigm is the association of certain behaviors with a preferred occurrence or situation, such as a child receiving a piece of candy with lunch because he did not fight with his sister in the car on the way to school.”*(1)p.166

The authors offer a proposed pathway to deviance and violent when children express poor behaviours that are consistently negatively reinforced.

*“Children who display frequent defiance and opposition to those around them are likely to be disliked and shunned. As adults and peers alike withdraw from contact with a child, he or she is less likely to receive reinforcement for the positive behaviors that are displayed. A lack of adult engagement places a child at risk for exposure to other rejected peers who are willing to engage the child. It is often through relationships with these deviant peers that a youth with a history of troubled relationships begins to commit criminal behaviors. Youth who commit many delinquent behaviors are likely to commit a variety of different kinds of offenses, including violent acts.”*(1)pp.166–167

Thus, suggesting that antisocial behaviour at a younger age is the *“best predictor of violence during adolescence”,*(1) the LIFT intervention aims to curb antisocial behaviour by rewarding positive behaviours. The authors do not, however, offer an exploration of the theoretical basis of positive reinforcement.

**Inputs**

There are three key inputs within the LIFT programme. The authors do not provide a theoretical rationale for any of these components separately, beyond loosely implying that, together, they will facilitate positive reinforcement of good behaviour in children.

The classroom component has lectures and role-plays, which are intended to teach children problem-solving skills. The playground component gives children time for free play. Positive and negative skills are then reviewed, and rewards for positive behaviours are given out. Finally, the parents’ training ensures that parents are aware of and trained in the curriculum, so that they can provide opportunities for problem-solving and positive behaviour for children in home-based activities.

Integration of the LIFT curriculum within the regular academic curriculum was not explored by the authors and was given no theoretical basis.

**Mechanisms of change**

As above, the authors are not explicit in the mechanisms of change that are expected to facilitated the desired outcomes of less delinquency and violence in young people. However, if we presume here that the opposite pathway than what is proposed through negative reinforcement occurs, then the pathway expressed through the blue boxes in Figure 1 are plausible. That is, positive behaviours occur, these are reinforced and are more likely to result in more positive behaviours. As a result, positive bonds with peers and teachers will result, with further reinforcement of positive behaviours as a result. Ultimately—to borrow the term “prosocial peers” from other interventions—young people are then likely to associate with prosocial peers and will be less likely to commit violent acts or act in a delinquent manner.

**Outputs**

The overall outputs of the programme are reduced delinquency and violent acts committed by young people.

**Assumptions**

The authors appear to make an overarching assumption that the opposite of negative reinforcement will occur with positive reinforcement, without providing a basis for this assumption.

**References**

1. Eddy JM, Reid JB, Fetrow RA. An Elementary School-Based Prevention Program Targeting Modifiable Antecedents of Youth Delinquency and Violence Linking the Interests of Families and Teachers (LIFT). Journal of Emotional and Behavioral Disorders. 2000;8(3):165-76.

**Peaceful Panels**

Peaceful Panels is an art curriculum that teaches communication and empathy to students. Students then create comics that reflect their understanding of conflict resolution. The programme critically engages with participants to think about how they view bullying and violence. Overall, the programme is expected to develop conflict resolution skills in participants for reduced school violence. Figure 1 below suggests a proposed logic model for the programme.


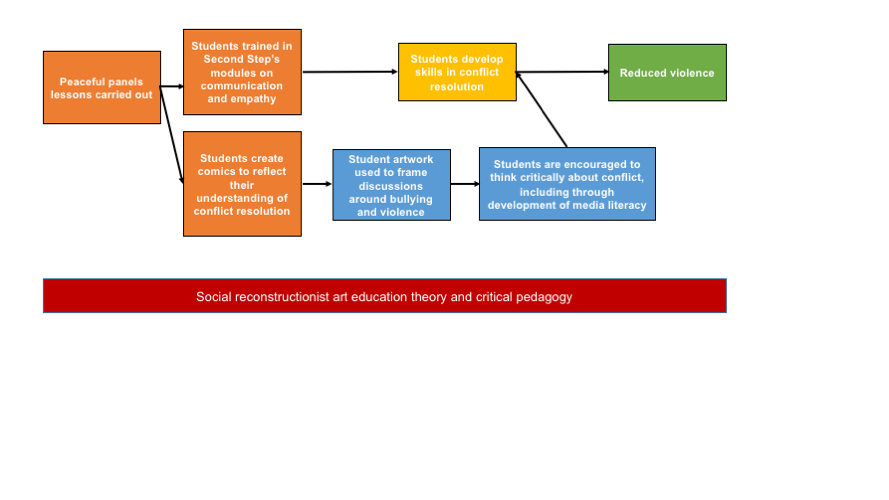


**Figure 1.** Proposed logic model for the Peaceful Panels programme

Orange = intervention inputs; yellow = proximal outcomes; green = distal outcomes (impacts); blue = mechanisms expected to facilitate outcomes; red = theoretical positioning

**Inputs**

The intervention is carried out in art classes. The major input is the art classes themselves. However, there are two components to these classes. The first is a conflict resolution lesson, modelled after the Second Step programme’s (1) lessons on communication and empathy. The second involves students creating comics to reflect their understanding of how to resolve a conflict.

The author provides some rationale for the integration of conflict resolution into art classes, specifically through the creation of comics. He suggests that, *“the reading, writing, and creation of comics have been found to be a successful strategy in teaching many other subjects. The reported success of using comics to teach school subjects [suggests that] a similar approach might be an effective way to teach violence prevention, an effective part of the reduction of bullying incidents in schools.”*(2 p.7)

Further, Wales offers that it is important that students receive lessons around violence reduction through various learning mediums, as students learn differently.

*“If schools do not employ diverse approaches to violence prevention education that include arts-based approaches, there is a risk of not reaching those students who are most engaged when those approaches are employed...there is a segment of the student population that is best reached through arts-based approaches.”*(2 p.9)

**Theoretical positioning**

The author draws heavily from social reconstructionist art education theory and critical pedagogy. The former suggests that art can be used to explore social issues and the latter suggests that classes can be built around student-teacher dialogue rather than didactic teacher-centered instruction. Taken together and applied to education, these aim to support critical thinking in young people and to empower them to change social conditions.

*“Social reconstructionist art education...curricula uses art to address social issues and combines students’ aesthetic sensitivity with a sense of social responsibility. Rather than teaching facts from institutionalized knowledge, social reconstructionist art educators seek to foster students’ critical thinking skills.”*(2 p.10)

In the Peaceful Panels curriculum, these approaches, therefore, guided teaching.

**Mechanisms of change**

A central theme to this programme is that art is considered an important medium through which student reflection on and analysis of situations of conflict can arise. Further, artwork can be used to frame a discussion on specific topic, in the case of Peaceful Panels, on conflict and bullying and transforming antisocial behaviours.

*“Art is useful as a diagnostic tool for teachers to discover student awareness of topics and can be used to promote reflection. The potential of using student drawings to promote reflection suggested that a strategy could be developed to teach violence prevention through comics. According to social reconstruction theory, art education should equip students to analyze and express opinions on issues and concerns that pertain to their own lives and to assume responsibility in creating a more equitable world.”*(2 p.23)

A secondary theme was also around critical thinking skills as they apply to the media and the use of art to develop media literacy skills among students, particularly in understanding bullying and violence.

*“Students are bombarded with media messages and they need to be taught to develop critical media literacy and to realize how these messages have impacted their identities. Using art-based educational approaches, teachers have guided students in an examination of how bullying and violence has been portrayed in popular media in order to help them learn to think critically about popular culture.”*(2 p.63)

**Outcomes**

The author is not explicit about exactly how the proposed mechanisms of change then lead to outcomes. The primary proximal outcome expected through the programme is improved conflict resolution among students. Students are then expected to engage in less conflict for reduced violence overall.

**References**

1. Frey KS, Hirschstein MK, Guzzo BA. Second step preventing aggression by promoting social competence. Journal of Emotional and Behavioral Disorders. 2000;8(2):102-12.

2. Wales A. Peaceful Panels: using comics to teach anti-bullying to junior high school students. Prescott Valley: Northcentral University; 2013.

**Positive Action**

Positive Action (PA) is a school-based intervention that teaches Skills for Successful Learning and Living (SSLL). A key theme of the programme is that students are expected to develop skills in order to better manage negative emotions and to exhibit a positive sense of self. In doing so, it is expected that their academic outcomes as well as their character will improve in the long-term, and that they will engage in less health-compromising behaviours, such as violence and substance use, which emerged as sub-themes with less direct influence within the programme.(1–8)

PA is delivered primarily through teacher training and a classroom curriculum, which was resoundingly the main theme emerging from PA literature around the programme’s inputs, although participating schools also have a “school climate” component, and the families of enrolled children can also access a family component, all of which are expected to reinforce core teaching around *“self-concept, positive actions for body and mind, social and emotional positive actions focusing on getting along with others, and managing, being honest with, and continually improving oneself.”*(1)p.8

The intervention is supported by theories of ‘triadic influence’ (2)—that behaviour is influenced by factors at different levels, proximal and distal—self-concept and self-esteem, effectively that positive actions will reinforce a positive sense of self, which will further result in positive actions. These theoretical underpinnings are reflected in Figure 1 below, which presents a logic model for the overall programme.


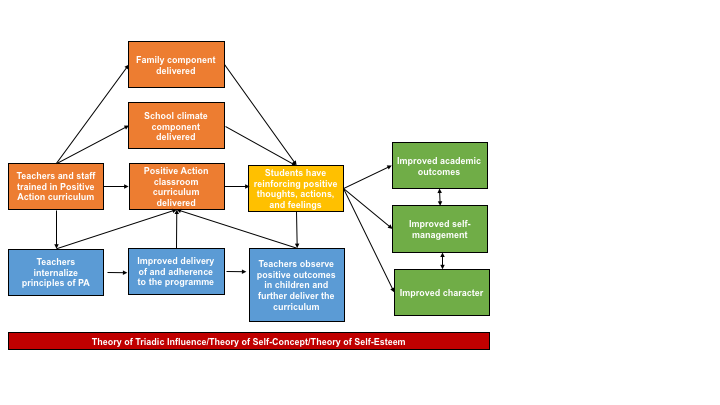


**Figure 1.** Logic model for the Positive Action intervention

Orange = intervention inputs; yellow = proximal outcomes; green = distal outcomes (impacts); blue = mechanisms expected to facilitate outcomes; red = underlying theoretical positioning

**Intervention inputs**

The intervention has four main components: training of staff and teachers; a family component; a school climate component; and the PA classroom curriculum. Emerging as key themes, however, are the training of teachers and staff and the classroom curriculum. The family component and the school climate component however are barely described, and the central building blocks of the programme’s theory of change appear to be the classroom component and teachers’ engagement with the PA curriculum.

The PA curriculum, *“teaches specific positive actions for the whole self: the physical, intellectual, social, and emotional areas (…) Together, these make up the comprehensive set of skills for successful learning and living (SSLL). The program trains teachers and parents to identify, teach, and reinforce positive thoughts, actions, and feelings about themselves by students and others in the school, leading to continual reinforcement of positive actions and enhanced student bonding with parents and school.”*(3)pp.476–477

As in the above extract, the curriculum targets six areas, most of which focus on developing a ‘sense of self’ among students and encouraging positive self-management of thoughts, actions, and feelings. The curriculum relies extensively on the Thoughts-Actions-Feelings (TAC) cycle, which—although introduced here as a key input—features also as part of the mechanisms of change within the intervention, as outlined later. The teaching of the TAC cycle and students’ application of it is seen as a fundamental aspect of what will ultimately change their character for the better in the long-term.

*“Using the Thoughts-Action-Feelings circle (see Figure 2), teachers, students and parents are taught to be aware of how they feel about themselves when they engage in an action, how this leads to further thoughts, how those thoughts lead to further behavior, and how they can change negative thoughts into positive thoughts to lead to positive behaviors.”*(4)p.30

Although parents/family and school climate components are mentioned in passing, they are explained to a much lesser extent:

*“The family involvement component leads to changes in opportunities for family involvement with the school focused on SSLL, as well as positive increases in the discussion and utilization of SSLL principles in school–parent and parent–child relations.”*(3)p.480

*“The school climate component leads to changes in school-wide activities such as reinforcement and recognition of positive behavior and character attributes demonstrated by students, as well as assemblies and other events that focus on SSLL.”*(3)p.480

*“The program trains teachers and parents to identify, teach, and reinforce positive thoughts, actions, and feelings about themselves by students and adults in the school.”*(1)p.8

Underlying all of the programme components are theories of triadic influence, self-concept, and self-esteem (2, 5, 6). These are primarily reflected in the classroom curriculum, although the school and family components are expected to reinforce resulting behaviours. All theories are very similar, suggesting overall that positive thoughts will lead to positive actions, or behaviours, which lead to positive feelings, or sense, of self, which motivates children and adults to repeat the entire cycle. This aspect of the theory is reflected in the Thoughts-Actions-Feelings cycle described. Of note is that, although the programme—as described by some authors—appears to be underpinned by the Theory of Triadic Influence and the Theory of Self-Concept, others are more forthcoming that the Theory of Triadic Influence, in particular, was not used in the planning of the intervention, but is simply a useful way of understanding it.

*“Although the Theory of Triadic Influence (TTI) did not guide the development of PA, the outcomes assessed by PA and the focus of the program can clearly be mapped onto the TTI.”*(1)p.8

**Desired outcomes**

There are proximal and distal outcomes theorised for the PA intervention as it is based on:

“*…a holistic approach to reducing behavior problems by addressing distal and proximal influences within the same program.*”(7)p.48

**Proximal outcomes: students have reinforcing positive thoughts, actions and feelings**

Once again, a key central theme was the reinforcement of positive thoughts, actions and feelings among children through the intervention.

***“****The Thoughts-Actions-Feelings About Self circle illustrates this self reinforcing process that is taught to students; showing them that thoughts lead to actions, actions lead to feelings about self, and feelings about self lead to more thoughts. The aim of PA is to get everyone into the positive cycle by consciously making positive choices. This is intrinsically motivated change, where a person chooses to do positive actions to feel good about his or her self.”*(1)p.7

**Distal outcomes: improved self-management, academic outcomes and character and reduced health-compromising behaviour**

Four subthemes could be identified under the theme relating to the expected distal outcomes of the PA programme. The first and more common subtheme emerging across publications was the outcome of improved self-management, which is seen to be pivotal to the other two distal outcomes, although all three are felt to be mutually reinforcing, as described below.

*1. Improved self-management*

Self-management refers to the ability to recognise feelings and thoughts in oneself and to then respond in a controlled and constructive way that is conducive to prosocial interactions. For example, a child that has just had their toy stolen may vocalise their disappointment and ask for the toy to be returned, rather than lashing out aggressively. It is then felt that by acting in a positive manner, the child will then feel positively about his or herself. This outcome is explicitly linked to the Thoughts-Actions-Feelings cycle introduced through the curriculum, which forms the core of the PA curriculum, and is the major outcome emphasized by authors.

“*The Thoughts-Actions-Feelings About Self circle illustrates this self reinforcing process that is taught to students; showing them that thoughts lead to actions, actions lead to feelings about self, and feelings about self lead to more thoughts. The aim of PA is to get everyone into the positive cycle by consciously making positive choices. This is intrinsically motivated change, where a person chooses to do positive actions to feel good about his or her self. Children and youth must not only be taught what is right versus wrong, but also need to be supported and reinforced for acting in such a way. The PA program does this in a way that is effective for students, as well as their instructors, parents, and community members*.”(1)p.7-8

*2. Improved academic outcomes*

As described more fully in the extract below, the PA curriculum’s intended outcomes are felt to have a positive impact on students’ academic performance although academic outcomes are not directly targeted:

*“The PA program is designed to teach students the positive actions in the physical, intellectual, emotional and social areas of their lives. The program specifically focuses on enhancing positive behavior, changing behavioral attributions directed at the self and social relationships, and the prevention of problem and health-compromising behaviors. In turn, these are hypothesized to lead to improved school-related performance.”*(4)p.29

*3. Improved character*

The third subtheme related to discussions about improved character, which was felt to be largely driven by improved self-management. This improvement was felt to be a longer-term, distal impact that would persist throughout a child’s life, into their adulthood, resonating in different areas of life and ultimately contributing to positive citizenship.

“*By building in relevance, Positive Action provides a foundation of strong, proactive behavior, character development, and academic achievement. Students gain social and emotional maturity and sound decision-making skills – aspects of a positive character that easily translate into active citizenship*.”(3)p.478

*“The Thoughts–Actions– Feelings Circle also helps students develop a positive character by teaching how important their values are to all aspects of their lives – including education (…). The goal is to help students value positive actions, like learning to achieve academically and becoming a good person, so that they can achieve success and happiness – or feel good about who they are, how they treat others, and what they are doing with their lives.”*(3) p.478

*4. Reduced health-compromising behaviours*

A fourth and final sub-theme was around reduced health-compromising behaviours, primarily substance use. Although substance use is not directly addressed through any aspect of the programme—it is absent from the curriculum and the school and family components—it is hypothesized to be reduced through the PA curriculum and the resulting outcomes that it achieves. Improved healthy behaviours are then expected to reinforce the key outcomes of the programme as indicated above.

*“The program targets the reduction of students’ health-compromising and other negative behaviors (e.g., substance abuse, violence, disciplinary referrals, suspensions), while simultaneously enhancing health-enhancing and other positive behaviors (e.g., honesty, time management) and behavioral attributions directed at the self (e.g., self-responsibility, positive self-concept) and social relationships (e.g., conflict resolution, respect/kindness), with these leading to improved school-related performance (e.g., improved attendance, academic achievement*).”(7)p.2

**Mechanisms of change expected to facilitate outcomes**

There are three interlinked mechanisms through which the PA intervention is expected to achieve the outcomes outlined above: the loop model between delivery and outcomes; the simultaneous influencing of the program on proximal and distal outcomes; and the improvement of student’s self-management and its relation to academic outcomes. All three mechanisms are centered on the idea that the internalization of the values of the PA overall philosophy, exemplified by the Though-Actions-Feelings Of Self Cycle, will bring benefit to all parties involved (teachers, students, family and the overall school). However, in the theory of change, notably, the most attention is given to the role of the teacher in implementing these mechanisms and, to some extent, of the student in manifesting the PA philosophy. Only brief mention is made of other aspects such as the role of enhancing of student and parent bonding and improving teacher-parent relationships.

*1. Role of teachers and the feedback loop between programme delivery and outcomes*

The primary theme emerging in relation to mechanisms revolves around the role of the teacher, and suggests the following feedback loop: once teachers have been trained in the PA curriculum, they will internalize the PA philosophy and will demonstrate it in their classroom 🡪 this internalization will, in-turn, lead to greater adherence to the curriculum 🡪 greater use of and fidelity to the PA curriculum will then increase the likelihood that the outcomes linked to positive thinking, acting and feeling will be demonstrated by students 🡪 and finally, once teachers see students behaving positively, they will be motivated to continue applying the curriculum.

*“Teachers deliver a program with enthusiasm and confidence towards topic comprehension or comfortableness (i.e., quality of delivery), do minimal modifications to the materials/content (i.e., adherence), and do so continuously within the classroom (i.e., dosage). Students in turn enjoy the program’s activities and message (i.e., responsiveness), reciprocating this back to the teacher in the form of engagement during the lessons and by exhibiting proposed program outcomes, such as intra- and inter-personal behaviors in the classroom (e.g., self-honesty, respect for others, improved academic performance). Teachers, in turn, observe that students are not only participating in the program activities, but also benefiting from the exposure, and decide to incorporate additional program materials and concepts into their curriculum.”*(7)pp.9–10

*2. Impacting proximal and distal factors*

Although not really emphasized as a key mechanism of change within the theory of change, although key to the Theory of Triadic Influence, which authors suggest is very applicable to PA, an identifiable sub-theme in the authors’ discussions relates to the lack of effectiveness of programmes that fail to address impacts at multiple levels. As such, contributing to the anticipated effectiveness of the PA intervention is its ability to impact proximal and distal factors, which affect the achievement of both proximal and distal outcomes. This is seen as a distinct characteristic of the PA program:

*“The goal is for students and adults to gain not only the knowledge, attitudes, norms, and skills that they might gain from other programs, but also improved values, self-concept, family bonding, peer selection, communication, and appreciation of school.”*(3)p.479

*“PA is designed to affect more distal (and more fundamentally influential) influences on school climate and student behavior and performance. The expected result is improvement in a broad range of behaviors (both negative and positive), emotional wellbeing, and school performance.”*(3) p.479

“*Once a behavior is performed, reactions to that behavior feed back into the different streams and levels of influence, as the experience of a behavior may change a person's self-control, self-efficacy, attitudes, bonds with others, beliefs about norms, etc*.”(1)p.9

*3. Improvements in self-management and student character leading to improvements in academic outcomes*

The final sub-theme regarding mechanisms of change is that improved self-management and character are expected to be linked to improved academic performance in students and vice versa. The bidirectional arrows between these distal outcomes in Figure 1 represents this mutually reinforcing relationship. Improved self-management provides a link to improved academic outcomes because children who are better able to self-regulate their emotions and behaviours demand less of the teachers’ attention in class, thus freeing up teachers’ time and energy to deliver academic sessions and improve academic skills in the classroom.

The program is, *“designed to target the enhancement of positive behaviors and behavioral attributions directed at the self (e.g., self-responsibility, positive self-concept) and social relationships (e.g., conflict resolution, respect, kindness), while simultaneously reducing students’ negative behavioral problems (e.g., substance abuse, disciplinary referrals, suspensions), with these leading to improved school-related performance (e.g., improved attendance, academic achievement).”*(7)p.73

**Moderating Factors**

Multilevel components in the PA programme are expected to moderate one another. In particular, these components are felt to mutually reinforce a type of school climate that is conducive to students’ social and character development. The characteristics of those responsible for implementing the intervention are thus felt to be of importance. As above, the role of the teacher is central to the intervention.

*“Implementation of the program components and, thus, effects on SSLL activities in the school, is moderated by school/administrator, teacher/staff, and family characteristics. Enhanced SSLL, in turn, impact student social and character development and supportive attitudes and skills (see Student Effects) both directly and through improvements in relevant facets of the school and classroom environment.”*(3)p.480

Finally, children’s household environments are felt to moderate not only the impact of the family component of the intervention, but their individual receptiveness to and internalization of PA. As a result, the need for reinforcement of taught skills in the classroom is emphasized:

*“Students coming from less effective homes (disadvantaged, high-risk, low parenting skills) have fewer of these [social-emotional] skills and need to have them taught. Standard education does little to compensate for lack of skills–they mostly teach the same way to all students regardless of student readiness to learn. Even students from homes where these skills are taught need to have them reinforced in school. Teaching these skills provides an opportunity for disadvantaged students to catch up and for students with the skills to practice and improve them.”*(3)p.477

**Assumptions**

Having an educational basis for PA, in which the curriculum is introduced and subsequently reinforced primarily through classroom activities is assumed to be effective.

*“Theoretically, it is expected that (a) learning SSLL is similar to learning other academic skills (i.e., initial learning can be enhanced over time if children are reinforced in applying the skills to increasingly complex situations regarding health, social relationships, and academics), and (b) learning and skill acquisition are best accomplished through a combination of direct instruction, interactive approaches, and engagement in positive activities, also characterized as sequenced, active, focused, and explicit.”*(3)p.474

Notably however, the fundamental theoretical tenet of the intervention, which posits that positive thoughts lead to positive actions and feelings of self, which reinforce each other, is, to some degree an assumption in and of itself. Authors do cite an evidence base for this thinking, but much is theoretical rather than empirical:

*“The program also assumes that when people feel positive about themselves, they will, in a reflexive manner, have more positive thoughts and engage in more positive behavior.”*(3)p.475

Moreover, based on further, broader assumptions about positive and negative values, and a clear distinction between good and bad behaviours (“*Children and youth must not only be taught what is right versus wrong, but also need to be supported and reinforced for acting in such a way”* (1)p.8, which is presumed to be universally shared.

“*Regardless of age, socioeconomic status, gender, or culture, students and adults all over the world suggest the same top values of respect, fairness, kindness, honesty, understanding/empathy, and love.”*(3)p.477

**References**

1. Lewis K. Evaluation of a social-emotional and character development program: methods and outcomes. Corvallis: Oregon State University; 2012.

2. Flay B, Petraitis J. The theory of triadic influence: An integrative model of substance use. Advances in Medical Sociology. 1994;4:19-44.

3. Flay B, Allred C. The Positive Action Program: improving academics, behavior, and character by teaching comprehensive skills for successful learning and living. In: Loval T, Toomey R, Clement N, editors. International Research Handbook on Values Education and Student Wellbeing. New York: Springer; 2010. p. 1040.

4. Flay B, Berkowitz MW, Bier MC. Elementary school-based programs theorized to support social development, prevent violence, and promote positive school climate: Description and hypothesized mechanisms of change. Journal of Research in Character Education. 2009;7(2):21-49.

5. Combs AW. Perceiving, behaving, becoming: A new focus for education. 1962.

6. DuBois DL, Felner RD, Brand S, Phillips RS. Early adolescent self-esteem: A developmental–ecological framework and assessment strategy. Journal of research on adolescence. 1996.

7. Beets M. Factors associated with the implementation fidelity of a school-based social and character development program: findings from the Positive Action Program, Hawai'i. Corvallis: Oregon State University; 2007.

8. Malloy M, Acock A, DuBois DL, Vuchinich S, Silverthorn N, Ji P, Flay BR: Teachers' perceptions of school organizational climate as predictors of dosage and quality of implementation of a social-emotional and character development program. Prevention Science. 2015;16(8):1086-1095.

**Promoting Alternative Thinking Strategies (PATHS)**

The PATHS program seeks to intervene in children’s development during their time in school, this being seen as a central part of life where positive change can take place. As an overarching theme, theories contributing to the development of PATHS stress the importance of ‘affect’ and emotional development as precursors to cognitive, verbal or academic learning. Hence it emphasises the role of emotional and social competence, self-management as well as neurobiological development as underlying structures that allow both academic skills development and the prevention of problem behaviours. Among these behaviours, the programme targets aggression, violence and substance use, which are assumed to result from maladaptation, neurobiological challenges and poor emotional intelligence manifested as lack of self-management.

**Key theoretical concepts and models**

The theory of change for the PATHS program is outlined as a series of five theoretical models seen as feeding into different aspects of the intervention. For each theoretical model, key themes and subthemes are briefly outlined below. However, it should be noted that these key theoretical principles are described by authors as parts of their respective theory, rather than clearly linked as building blocks of an overall theory of change for PATHS. Authors are often not explicit about how these theoretical models translate into the PATHS programme—that is, what type of inputs or outcomes might be expected within PATHS as a result.

*1. The Affect, Behaviour, Cognition, and Development (ABCD) model*

The fundamental tenet of the ABCD model (1), is that affective development precedes learning. Children in pre-school years (0-3) start off by experiencing feelings before they can verbalise them. They then begin to verbalise their feelings and develop ‘affective perspective-taking skills’, that is, the ability to observe others’ feelings and reactions. Between the years of 5-7, children learn to become more independent, and understand their roles and responsibilities in social interactions. During the elementary school years, there is a strengthening of the integration between emotions, behaviour and language.

The ABCD model specifically focuses on, *“the promotion of optimal growth and on the developmental integration of affect (i.e., emotion, feeling, mood), emotion language, behavior, and cognitive understanding.”*(2)p. 437

Although authors make no explicit links between this model and the implementation of the PATHS programme, the PATHS curriculum covers five domains: self-control; emotional understanding; positive self-esteem; healthy relationships; and interpersonal problem-solving skills. Specifically, the domain of emotional understanding seems to fit well within the goals of the ABCD model, although this is an inference made by us. As such, we propose the following logic model element for PATHS, aligning with the ABCD model (Figure 1).

Of note, however, is that much of the ABCD model emphasises the pre-kindergarten age range. PATHS does have a pre-kindergarten curriculum, although none of the empirical studies we included in this review focus on that age period because unlike the grade five curriculum, the pre-kindergarten curriculum does not integrate health and literacy education. As such, this model may be of less relevance when considering the theory of change that underlies the grade five PATHS programme.


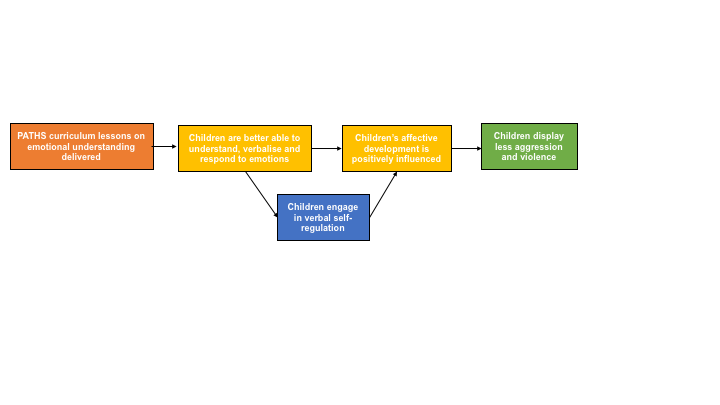
**Figure 1.** Logic model for the ABCD model applied to the PATHS programme

Orange = PATHS programme inputs; yellow = proximal outcomes; green = distal outcomes (impacts); blue = mechanisms of change

2. *The Eco-Behavioural Systems Model*

Children develop within environments and, in keeping with eco-behavioural systems (3) model, authors posit that children-centred interventions that do not address the overall school environment will miss out on the important developmental benefits of teaching and reinforcing skills at multiple levels. Reinforcement of skills introduced through PATHS is a key theme within this model. Specifically, providing learning and application opportunities that integrate affect, cognition and behaviour are encouraged.

*“Ecologically oriented programs emphasize not only the teaching of skills, but also the creation of meaningful real-life opportunities to use skills and the establishment of structures to provide reinforcement for effective skill application.”*(4)p.399

Considering multiple levels, teachers, as well as school staff and parents, are trained in the PATHS curriculum not only to establish positive and responsive classroom, school and community environments that support children’s development, but also so that such skills may be modelled and generalised at each of these levels. Generalisation of the skills introduced through the PATHS curriculum is a second key theme within this model.

*“The extensive generalization procedures, teacher training, and focus on some level of parent participation used in PATHS have the goal of combining classroom instruction with efforts to create environmental support and reinforcement from peers, family members, school personnel, behavioural health professionals, and other concerned community members.”*(4)p.399

Within PATHS, both reinforcement of skills and generalisation of skills, which are mutually beneficial, are emphasised to teachers through their training, which encourages the role modelling of key skills by teachers. This is theorised as facilitating similar skill developing among the children.

*“Training emphasizes the manner in which the teacher uses the curriculum model and generalizes the skills to build a healthy classroom atmosphere (i.e., one that supports the children’s use and internalization of the material they have been taught).”*(4)p.399

This model is outlined in Figure 2 below, which proposes another element of the logic model for how this theory applies to PATHS.


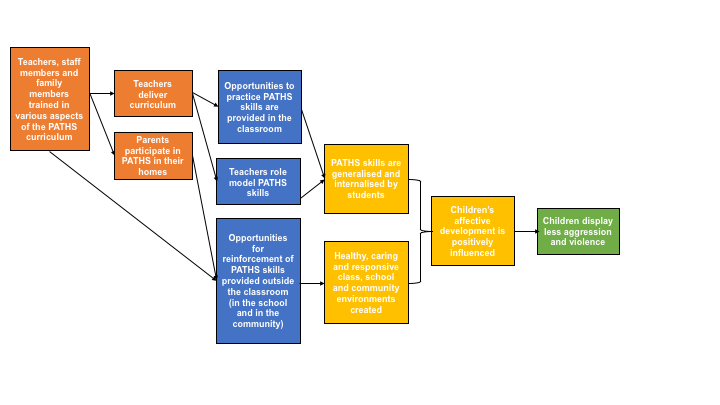


**Figure 2.** Logic model for the Eco-Behavioural Systems model applied to the PATHS programme

Orange = PATHS programme inputs; yellow = proximal outcomes; green = distal outcomes (impacts); blue = mechanisms of change

*3. Neurobiology and Brain Structuralisation and Organization*

Another important theme in the literature on the PATHS program relates to two key notions drawn from neurobiological theories of brain structuralisation and organisation (5). The first subtheme of relevance relates to the notion that, due to particular ways in which their brain develops, children can be actually unaware of, or unable to recognise, their feelings. The second subtheme of interest relates to the idea that affective and social functioning are controlled by parts of the brain that are distinct from those in charge of cognitive development, hence children can perform well cognitively and function academically but continue to struggle with social functioning.

Authors mention that the PATHS programme employs teaching strategies that promote brain structuralisation/organisation aimed at supporting the development of self-control. It does these by: a) supporting the ‘horizontal’ structures of communication in the brain through labelling exercises that teach children to name and recognise their feelings; and b) building up the ‘vertical’ structuralisation in the brain that is responsible for regulating emotion and controlling impulse.

*“Thus, PATHS incorporates the use of Feeling Face cards that include both the facial drawing of each affect (recognition of which is mediated by the right hemisphere) and its printed label (which is mediated by the left hemisphere). Additionaly, a color-coded differentiation of comfortable (yellow) versus uncomfortable (blue) feelings is utilized. These cards facilitate and encourage verbal discussion of emotional experiences (both at the time they are occurring and in recollection), which further strengthens neural integration.”*(4)p.401

*“Given the crucial importance of the optimal development of “vertical” control, several strategies are utilized in PATHS to teach children self-control in ways that theoretically promote neuronal structuralization and growth between the frontal cortex and limbic system. These paradigms are designed to be developmentally appropriate, beginning with relatively simple motor control in the early years (the “Turtle Technique”), followed by more complex models with increased use of language and cognition (Control Signals Poster and formal problem solving) that build on one another as the children mature.”*(4)p.400

**
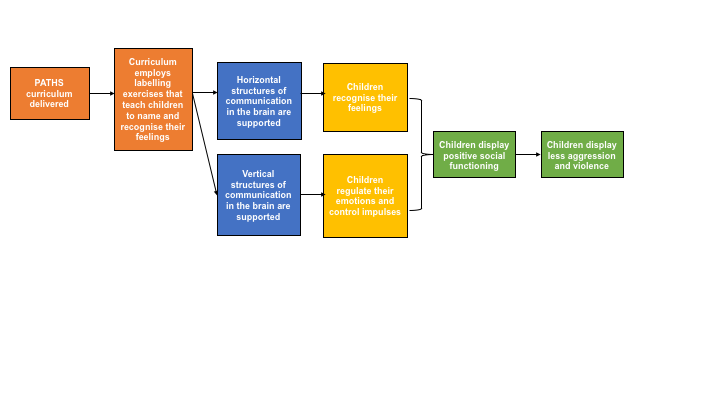
**This model is outlined in Figure 3 below, which proposes a part logic model for how this theory applies to PATHS.

**Figure 3.** Logic model for the Neurobiology and Brain Structuralisation and Organization model applied to the PATHS programme

Orange = PATHS programme inputs; yellow = proximal outcomes; green = distal outcomes (impacts); blue = mechanisms of change

4. *Psychodynamic Education*

PATHS draws from psychoanalytic theory and, particular, the Theory of Psychodynamic Education (6). A key theme here is how education can enhance affective development. Several techniques are used in psychodynamic education within the context of PATHS. These are: establishing positive student-teacher relationships; students internalising prosocial values; teachers encouraging student creativity and imagination; students having optimal emotional and cognitive integration and expressions of affect; and the establishment of learning as a process of joyful discovery. The role of the teacher is central to this theory. The authors suggest that, given a positive relationship between primary school students and teachers, lessons introduced by teachers will be seen as an absolute truth. Coupled with the demonstration of the very prosocial behaviours encouraged by the PATHS curriculum by teachers, it is theorised that students will be much more likely to adopt similar prosocial behaviours. Likewise, the role-modelling of positive behaviours by their peers is felt to elicit such behaviours in students.

Secondly, through the PATHS curriculum, students are encouraged to understand and verbalise their emotions to peers and teachers, which is also encouraged through psychodynamic education. In doing so, children are given platforms to discuss their feelings, which enables them to feel, *“listened to, supported, and respected by both teachers and peers, which facilitates the internalization of feeling valued, cared for, appreciated, and part of a social group.”*(4)p.402

This sense of belonging also emerges as a sub-theme within the Theory of Psychodynamic Education as it applies to PATHS, as it is also felt to inspire children to treat others kindly and respectfully.

*“Among other things, this application of psychoanalytic theory emphasizes positive teacher-student relationships, internalization of prosocial values, use of creativity, optimal emotional and cognitive integration, appropriate expression (rather than repression) of affect, and learning as a process of joyful discovery. It can be noted that the incorporation of psychoanalytic theory distinguishes PATHS from the vast majority of other SEL curricula.”*(2)p.437

*“Because research strongly suggests that learning experiences in the context of meaningful relationships influence the development of neural networks, PATHS strategies are designed to optimize the nature and quality of teacher-child and peer-peer interactions (Kusché & Greenberg, 2006a). When children feel listened to, supported, and respected by both teachers and peers, they internalize being valued, cared for, appreciated, and part of a social group. This, in turn, motivates children to value, care for, and appreciate themselves, their environment, their social groups, other people, and their world (a crucial antidote to violence).”*(2)p.439

Another sub-theme that emerges is self-motivation to regulate actions so that good behaviours become internalised. This is theorised as being brought about by positive social relationships and teacher and peer role-modelling. The PATHS programme stresses the importance of promoting that the children own the ‘internalization’ of prosocial values and concepts, so that through ownership they will adopt them as their own (rather than being told that is how they are meant to behave). Moreover, children are taught the importance of prosocial values, why these matter and how they are beneficial to relationships, so they are given the opportunity to endorse them at a deeper level rather than merely enacting them because *that is what they are supposed to do.*

*“Teachers are encouraged to utilize actual classroom experiences, and the use of children's creative, imaginal processes is emphasized. Students develop a healthy sense of self-esteem from observing the positive reactions of others towards them, not because they are encouraged to parrot simplistic affirmations.”*(4) p.401

*“An important way in which psychodynamic education differs from other models is its emphasis on internalization. Among other important uses, internalization is the primary process utilized in the development of an individual’s conscience. When the outcome is positive, the conscience (specialized functioning in the preorbital frontal lobes) works as a powerful system through which a person can “take ownership” and achieve mastery over his or her own impulses and actions.”*(4) p.401

This model is outlined in Figure 4 below, which proposes a part logic model for how this theory applies to PATHS.

**
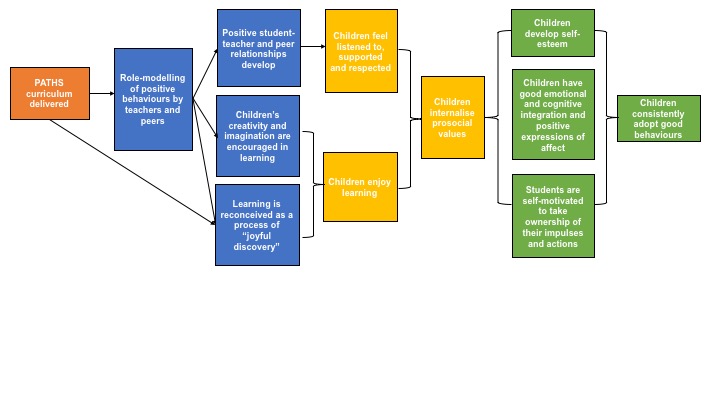
**

**Figure 4.** Logic model for the Theory of Psychodynamic Education applied to the PATHS programme

Orange = PATHS programme inputs; yellow = proximal outcomes; green = distal outcomes (impacts); blue = mechanisms of change

5. *Psychology of Emotional Awareness*

A final theme concerns the centrality of the theory of the psychology of emotional awareness (which the authors also refer to as emotional competence and emotional intelligence)(7). Emotional intelligence relates to children’s ability to understand the causes of their emotions while they are experiencing them. Although children might have strategic plans for how they respond emotionally to certain situations, these can be detailed in real-life situations. Emotional intelligence allows children to understand their own emotions in the moment and find ways to act in a positive way accordingly. As with many of the other theories introduced thus far, central sub-themes here relate to how this is promoted by modelling and reinforcement by peers and adults—especially teachers.

*“Children’s ability to understand their own and others’ emotions is a central component of effective problem-solving and social interactions. When emotional awareness is not sufficiently emphasized in SEL programs, children can model optimal problem-solving skills as a cognitive exercise, but do not utilize them when strong emotions are experienced during real life experiences. Children must be able to effectively regulate their emotional arousal and accurately process the emotional content of a situation in order to successfully employ cognition to solve a problem.”*(2)p. 439

As in the discussion of psychodynamic education, this theory also emphasises the ways in which children can learn to value themselves and others when they are listened to, feel valued and are allowed to discuss their feelings. Furthermore, empathy and respect for a broader community (that extends beyond the classroom) are also explicitly promoted by the PATHS programme to prevent divisions arising between in- and out-groups.

Authors describe how a sense of belonging to a broad human community can deter violent behaviours.

*“It can be noted that a sense of belonging is a powerful, biologically-based deterrent to the enactment of violent behaviors towards other group members. However, this does not prevent destructive actions and maltreatment towards members of “other” groups (e.g., the basis of gang warfare, genocide, etc.). Thus, in addition to reinforcing inclusion in smaller prosocial groups (e.g., the classroom, the school, etc.), we also emphasize an awareness of larger group memberships, such as constituency in the local neighborhood, the global community, and as part of life on our planet.”*(4)p.403

This model is outlined in Figure 5 below, which proposes a part logic model for how this theory applies to PATHS.

**
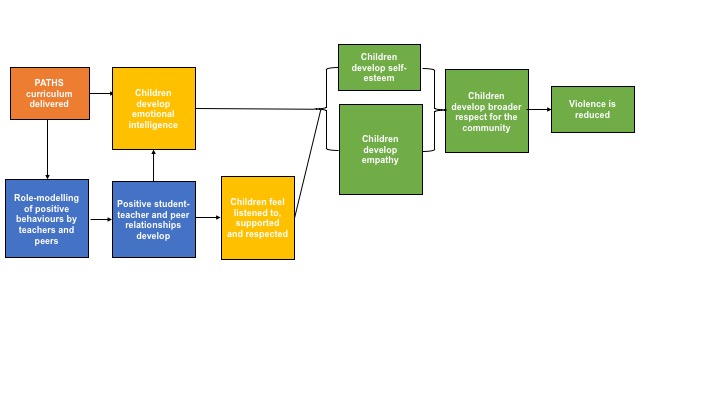
**

**Figure 5.** Logic model for the psychology of emotional awareness applied to the PATHS programme. Orange = PATHS programme inputs; yellow = proximal outcomes; green = distal outcomes (impacts); blue = mechanisms of change

*6. Social Information Processing Theory*

Although most of the PATHS literature refers to the five theoretical models outlined above an additional theoretical contribution (Social Information Processing Theory (8, 9) is introduced by Crean (10) to link affective issues of development to aggressive behaviours:

*“*[Social Information Processing] *models of aggressive behavior were developed to describe at a proximal level how cognitive and emotional processes lead a child to engage in aggressive behavior. Here, attribution of intent and social problem solving abilities (generating alternative response options, evaluating responses, selecting the preferred option, and then carrying out the selected response) are fundamental and both are targets for change in the PATHS intervention.”*(10)p.58

The authors also note that, *“the PATHS curriculum’s central focus on identifying and understanding emotions and effective social problem solving resonates closely”*(10)p.58 with the theory, referring specifically to aspects of the curriculum within the domains of emotional understanding, healthy relationships and interpersonal problem solving skills.

**Drawing the Theories Together**

There are a number of common themes in the five above theories (excluding the Social Information Processing Theory, as authors do not explicitly indicate that it informed the programme), derived through axial coding between the theories.

*Inputs*

In terms of inputs, clearly PATHS itself is common to all of the theories, with different domains or aspects of the curriculum being important for each. However, all five domains featuring in the PATHS curriculum—self-control, emotional understanding, positive self-esteem, healthy relationships, and interpersonal problem-solving skills—are highlighted throughout the above theories.

*Mechanisms of change*

Although there are a number of different mechanisms of change highlighted throughout, recurring ones concern: the importance of teachers and peers role-modelling PATHS skills and behaviours; the development of healthy student-teacher and peer relationships; and opportunities for reinforcement of PATHS skills and behaviours emerging through positive classroom, school, and family environments.

*“Thus, although a central goal of PATHS is to promote the developmental skills of each child by providing learning that integrates affect, cognition, and behavior, a critical ingredient for success is the development of healthy, caring, and responsive classrooms within the context of the school environment.”*(4)p.399

*Proximal outcomes*

These concern the specific skills or behaviours that children are expected to demonstrate after receiving the PATHS curriculum, and that are promoted through some of the mechanisms of change described above. These largely centre around: development of emotional intelligence; students being able to control their emotions and self-regulate; students internalising PATHS skills, behaviours, and prosocial values; and students generally feeling valued, specifically through feeling respected and listened to.

A major contribution of the programme to children’s development is that they are expected to be able to better identify, process and respond to cues that would ordinarily trigger an aggressive or violent emotional response. It is expected that, over time, this positive response—called ‘social competence’—will become second nature to the children. In Figure 6, this aspect is emphasised as ‘internalisation’ by the child. It is also highlighted in Figure 4 as the internalisation of prosocial values, and underlies the development leading to less violence and aggression in children expressed in the ABCD Model (Figure 1).

*“For example, in the early elementary years, a child with well-developed social-emotional skills who has been rebuffed when attempting to enter a game with peers might walk away, calm down, and assess how both s/he and the other kids are feeling. The child might then think about alternative solutions to the problem such as another strategy for entering the game, finding something else to do, or asking someone else if they want to play. Moreover, for the socially competent child, most of this process will be primarily “automatic” and will not require focused conscious meta-awareness.”*(4)p.398

*Distal outcomes (impacts)*

Finally, although some of these distal outcomes are expressed as proximal outcomes in some theories, they are largely grouped around children having higher self-esteem, positive expressions of affects, consistent adoption of good behaviours (which is inclusive of healthy behaviours, such as avoidance of substance use), and finally, less aggression and violence.

Taken together, these various aspects of the individual theories’ logic models can be brought together into our suggested logic model for PATHS, a synthesis of all five in Figure 6 below.


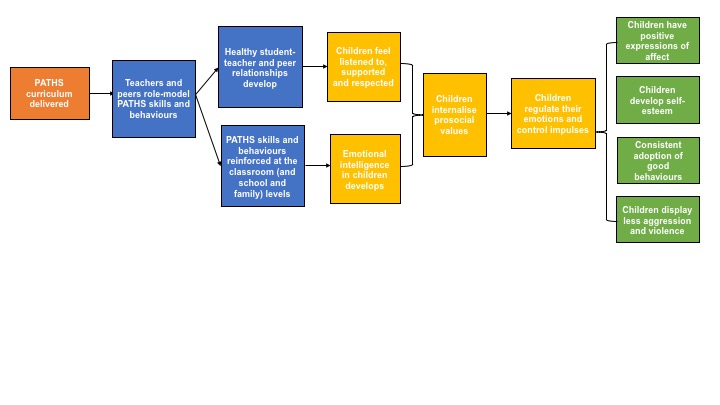


**Figure 6.** Overall logic model for the PATHS programme after synthesis of its five core contributing theories

Orange = PATHS programme inputs; yellow = proximal outcomes; green = distal outcomes (impacts); blue = mechanisms of change

**Integration of PATHS into the academic curricula**

The theme of integration or its rationale are not very developed in the theories that the PATHS programme relies on. However, authors do report the training of teachers in PATHS materials, with suggestions on how to use high quality reading materials to teach the programme skills as part of broader academic learning activities.

*“PATHS provides specific ideas and materials for integration of concepts through other academic areas and teachers report that this is an often overlooked, yet critical, area for school-based prevention programming”*(10)p.57

*“PATHS was designed to be taught by regular classroom teachers (initially with support from consultant trainers) as an integrated component of the regular school curriculum.”*(4)p.4

*“Finally, language arts (both reading and writing) are bridged to PATHS in most lessons by including supplementary suggestions for teachers to utilize to reinforce lesson concepts, such as children’s literature. Further, one of the chapters in the instructional manual provides many ideas for how teachers can directly tie PATHS concepts to language arts, social studies/history, and other subject areas.”*(4)p.404

From personal communication between the study team and Mark Greenberg, it was confirmed that the only the grade five curriculum places emphasis on developing language and literacy skills as part of the PATHS curriculum. Otherwise, the only opportunities for integration are those expressed above, which rely largely on teachers taking the initiative to integrate material into regular classroom lessons. Thus, integration may or may not occur outside of the grade five curriculum.

**Assumptions**

Although not highlighted by other authors, Crean et al (10)p.57 point out that underlying the PATHS programme, there are four assumptions:

*“The following four assumptions guide the PATHS prevention strategy: (1) Children’s ability to understand and discuss emotions is related to both communicative development and the ability to inhibit behavior and show self-control; (2) Children’s ability to manage, understand, and discuss emotions operate under developmental constraints and is also affected by socialization practices; (3) Children’s ability to understand their own and others’ emotions is a central component to effective social problem solving; and (4) The school environment is a fundamental ecology that can be a central focus of change*.”(10)p.57

Two further underlying viewpoints emerged from our analysis. First, mention in the text that without internal emotional self-regulation at the individual level there would be chaos in social interactions, requiring extensive external regulation.

*“By promoting the development of internal self-control and self-motivation, along with healthy standards for behavior, children develop autonomy, mature decision-making skills, and the ability to consider the needs and feelings of others. These achievements are crucial for violence prevention and school safety, because they allow individuals to control themselves. There will never be enough police or teachers to provide the external control that is alternatively necessary if internal controls have not been incorporated or are not operative.”*(4)p.401

Seecond, a statement regarding the consequences of a lack of a sense of community, which could mean that small-group bonding (such as that promoted in a classroom) could resolve in-fighting amongst groups through a process of ‘othering’.

*“In PATHS, we encourage classroom cohesion through regular activities including ongoing complimenting, stories, and teaching sharing as a positive endeavor. However, the same need for group membership can also promote destructive actions and maltreatment towards members of “other” groups (e.g., the basis of gang warfare, genocide, etc.). Thus, in addition to reinforcing classroom inclusion, we also emphasize an awareness of larger group memberships (i.e., neighborhood, global community, and as part of life on our planet) and the nurturing of compassion towards others.*”(2)p.439

**References**

1. Greenberg MT, Kusché CA. Promoting social and emotional development in deaf children: The PATHS project: University of Washington Press; 1993.

2. Kusché C, Greenberg M. The PATHS curriculum: promoting emotional literacy, prosocial behavior, and caring classrooms. Handbook of school violence and school safety: international research and practice. 2012:435-46.

3. Elias MJ. Promoting social and emotional learning: Guidelines for educators: Ascd; 1997.

4. Greenberg M, Kusché C. Building social and emotional competence: the PATHS curriculum. In: Jimerson S, Furlong M, editors. Handbook of School Violence and School Safety. 1. New York: Taylor & Francis Group; 2006. p. 712.

5. Kusché CA, Greenberg MT. Brain development and social-emotional learning: An introduction for educators. The Educator’s Guide to Emotional Intelligence and Academic Achievement M Elias & H Arnold, Eds: Corwin Press Thousand Oaks, CA. 2006.

6. Kusche C, Riggs N, Greenberg M. PATHS: Using analytic knowledge to teach emotional literacy. The American Psychoanalyst. 1999;33(1).

7. Goleman D. Emotional intelligence. New York, NY: Bantam. 1995.

8. Huesmann LR. An information processing model for the development of aggression. Aggressive behavior. 1988;14(1):13-24.

9. Dodge KA, Coie JD, Lynam D. Aggression and antisocial behavior in youth. Handbook of child psychology. 2006.

10. Crean HF, Johnson DB. Promoting Alternative Thinking Strategies (PATHS) and elementary school aged children's aggression: results from a cluster randomized trial. Am J Community Psychol. 2013;52(1-2):56-72.

**Raising Healthy Children**

Raising Healthy Children (RHC) is an intervention that uses four components to strengthen skills for resisting negative peer influences and development of prosocial bonds that are seen as protective against risk of substance use. These components are: 1. staff and teacher training in RHC programme and monthly coaching; 2. parenting workshops to decrease familial conflict and to clarify expectations around substance use; 3. peer intervention strategies through classroom activities, summer camps, and retreats aimed at developing social-emotional learning; and 4. student interventions like after-school tutoring, study clubs in grades 4–6, and workshops teaching academic skills, prosocial bonding, substance refusal skills, and developing healthy behaviours. Together, this multi-level approach is expected to establish strong bonds between children, teachers and parents.

It is postulated that: *“after* *strong bonds are established, individuals will tend to behave in a manner consistent with the norms and values of the individuals and groups with whom they associate. In turn, stronger prosocial bonds support positive belief formation against antisocial behaviors (e.g. adolescent substance use).”*(1)p.700


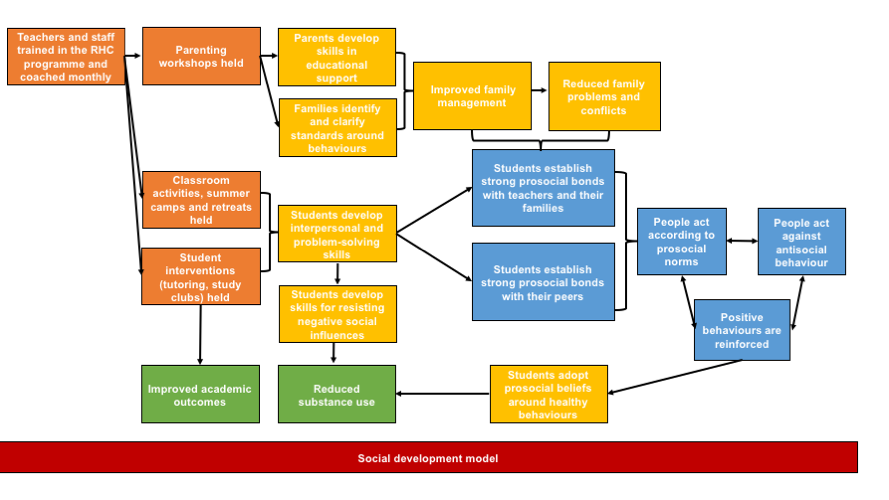


**Figure 1.** Logic model for Raising Healthy Children

Orange = intervention inputs; yellow = proximal outcomes; green = distal outcomes (impacts); blue = mechanisms expected to facilitate outcomes; red = underlying theoretical positioning

The logic model in Figure 1 above presents a slight oversimplification of the programme to highlight the main proposed effects of RHC at two targeted points in children’s lives: the early (elementary school) years, and the later (middle and high school) years. In reality, the overall outcomes of the programme are meant to be achieved in both periods, but particular emphasis is given to developing prosocial bonds, especially with the family and school, in early years, and developing prosocial bonds with peers, as well as minimising favourable attitudes to substance use, in later years. The outcome of decreasing substance use, particularly through establishing norms against substance use, is given weight in later years.

**Key theoretical concepts**

The RHC programme is based on the social development model (SDM),(2) itself supported by social control, social learning and differential association theories. Within the SDM framework, early intervention is emphasised, alongside the broader social context in children’s life (for example family, peers and community). However, the main theme is related to the changing phases of development which is linked to the need for a flexible programme design that reflects aspects of children growth into adolescence.

“*The SDM emphasizes that prevention should (…) identify and address the changing needs of its target population with regard to risk and protective factors that change in influence during the course of development.”*(1)p.700

**Intervention points (inputs)**

*The involvement of prosocial others*

As the main theme in the theory of change for the RHC program is the central notion that prosocial bonds are key to preventing risk of substance use Therefore, creating opportunities for engagement with “prosocial others”—explained as family members, teachers, and non-substance using peers—in children’s development is one of the four main targets of RHC. As above, engagement with family members and teachers is emphasised in earlier years, while engagement with pro-social peers is emphasised in later years. This important aspect of RHC is described more fully in domains of change below.

*Other areas of input*

The remaining three area of input mentioned are the teaching of academic, cognitive and social skills; providing positive reinforcements for prosocial behaviours; and setting beliefs and standards around substance use avoidance. In later years, the final inputs around substance use avoidance also include the teaching of skills to resist negative social influences. Critically, these three additional inputs are felt to reinforce prosocial bonding.

However little explanation is offered of how these are understood or implemented, though some information can be inferred from the discussion of the mechanisms of change below.

**Domains of change**

Adapting the programme to changes in students’ life-stages influences both the inputs and the expected outcomes as the targets for intervening in children development shift. Two key periods are discussed, namely childhood and adolescence, coinciding with school years.

In early childhood, *“the primary domains of social influence during elementary school years are theorized within the SDM to be the family and school”* hence the *“RHC intervention components during this period focused on [family and school]”*,(1)p.700 specifically, on the prosocial bonds developed there.

The authors base this on previous evidence of early interventions, showing that, *“teachers reported less disruptive and aggressive behavior and stronger effort on school work for intervention students compared to controls.”*(1)p.700

More discussion is devoted to the transition to adolescence, when peer relationships become key areas to intervene in.

*“Preventive interventions that target norms and teach skills for resisting negative social influences during this period* [adolescence] *have been shown to be effective in reducing substance use. Thus, the constellation of intervention components within RHC gradually shifted from early risk and protective factors in the social domains of school and family (e.g., academic performance, bonding, and parental monitoring) toward individual- and peer-related risk and protective factors (e.g., refusal skills, healthy beliefs, and associations with substance-using peers).”*(1)p.700

Of note, a subtheme in the discussion of domains of change and different phases of adolescence relates to the potential usefulness of addressing risk and protective factors for substance use not only in relation to absolute use, but also aiming for a reduction.

“*Whereas preventive interventions for early-adolescent substance use often center around abstinence themes, once adolescents begin to use substances, messages related to the prevention of escalating or problematic substance use become increasingly important. Furthermore, recent data have shown that some degree of experimentation with substances is normative. Noting this, an increasing number of researchers have suggested that a concomitant goal of prevention should be the reduction in the amount of use (quantity or frequency) among users. As the prevalence of substance use increases typically during adolescence, a corresponding increase in the frequency of use is likely. Thus, social development approaches to the prevention of substance use address risk and protective factors not only for initial and experimental use, but for heavy or problematic use as well.”*(1)p.700

**Mechanisms of change**

Through the development of multiple prosocial bonds with teachers in the school, with family and with peers, the RHC programme is expected to: “*(a) improve academic achievement, (b) increase students’ bonding to school, (c) teach refusal skills, and (d) develop prosocial beliefs regarding healthy behaviors.”*(1)p.701

As well as strengthening relationships between teachers and students, the programme targets changes at the family level, developing ways to: “*(a) enhance parents' skills in child rearing and educational support, (b) decrease family management problems and conflict, (c) identify and clarify family standards and rules regarding student behaviors (e.g., substance use, dating, and sex), and (d) practice peer resistance skills.”*(1)p.701

At the peer-two-peer level, the RHC programme activities cover a range of interactions through, *“classroom instruction and annual summer camps during elementary school, and social skills booster retreats in middle school (…) for students to learn and practice social, emotional, and problem-solving skills in the classroom and other social situations.”*(1)p.701

The RHC programme is seen to be effective at improving students’ prosocial bonds at multiple levels (school, family, peers). These stronger prosocial bonds overall are felt to result in people acting according to (pro)social norms. These, in turn, are expected to be protective against antisocial behaviour.

However, the pathway between the development of prosocial norms and bonds and a reduction in substance use is not explained in any greater detail.

**Outcomes**

Through the flexible mechanisms outlined above, tailored to different phases of students’ growth, the RHC programme targets a reduction in substance. Further, the multi-level intervention including family and prosocial peers is seen as a pathway to achieving a variety of interlinked outcomes including, but not limited to, prevention of substance use:

*“Through the combined use of school, student, peer, and family intervention strategies, RHC sought to reduce risk factors of poor family management, family conflict, early antisocial behavior, academic failure, low commitment to school, associations with substance using peers, and favorable attitudes toward drug use; and enhance protective factors of bonding to family and school, setting healthy beliefs and expectations, and teaching social and emotional skills.”*(1)p.701

**Assumptions**

Throughout, the authors make the assumption that bonds with prosocial peers will reduce substance use. Although RHC also emphasises the need to educate around the dangers of substance use in order to normalise non-substance use, there still seems to exist the leap between simply encouraging bonding and decreasing the use of substances, which is not necessarily founded.

Another assumption that the programme makes is that risk and protective factors change throughout development, and thus, the programme’s targets need to change accordingly. This assumption forms the basis of the rationale behind targeting of two different domains within the programme.

**References**

1. Brown EC, Catalano RF, Fleming CB, Haggerty KP, Abbott RD. Adolescent substance use outcomes in the Raising Healthy Children project: a two-part latent growth curve analysis. Journal of consulting and clinical psychology. 2005;73(4):699.

2. Catalano RF, Hawkins J. The social development model: a theory of antisocial behavior. In: Hawkins J, editor. Delinquency and Crime. New York: Cambridge University Press; 1996. p. 149–97.

**Roots of Empathy**

**
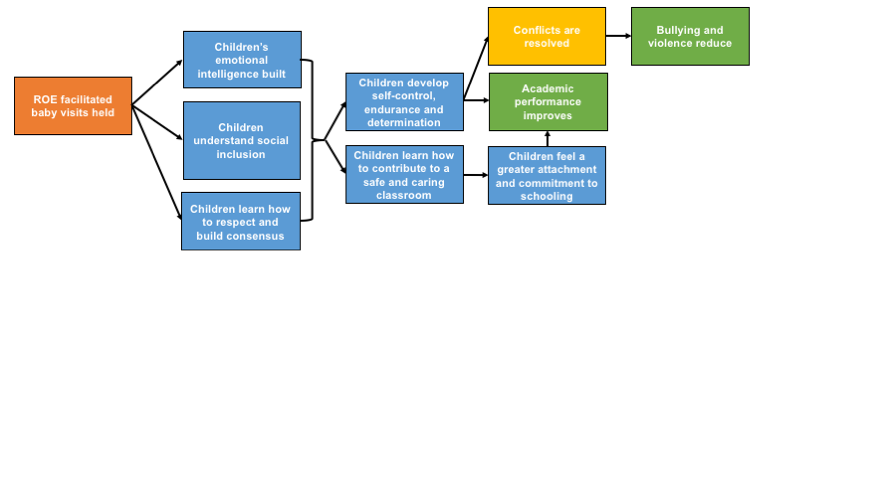
**Roots of Empathy (ROE) is a programme that brings a visiting baby and one or both parents into a classroom at repeated times throughout the year as a springboard for teaching empathy to students with the help of a trained ROE instructor. The programme aims to support the development of social and emotional learning (SEL) that will improve students’ connection to their school and each other. Whilst becoming involved with the baby’s development, needs and temperament, students learn about behavioural, health and substance issues also. Figure 1 below outlines a logic model for the programme.

**Figure 1.** Proposed logic model for Roots of Empathy

Orange = intervention inputs; yellow = proximal outcomes; green = distal outcomes (impacts); blue = mechanisms expected to facilitate outcomes

**Rationale for integration**

An important theme in the discussion of the theory of change of ROE interventions is the central role played by SEL in academic learning. The integration of a broad range of SEL skills, concepts and practices is seen as creating the ideal platform for students to learn academically. There are attempts to link the ROE curriculum into aspects of the academic curriculum for greater ease of integration. Hence the rationale for integration in the programme is theoretical and scientific as well as practical, concerning both concepts and delivery modes.

*“There is an increasing emphasis on the need for teachers to embrace strategies that support the growth and development of social outcomes for children while helping them deal with the increasing challenges of today's classrooms (…) This can be achieved by embracing social emotional learning (SEL) concepts and programs that balance reform agendas and recognise the goals of education as producing ‘academically competent, responsible, productive, and caring students and citizens‘.”*(1)p.53

Evidence is offered of the need to implement SEL-centred programs.

*“Schools can be reassured by scientific and pragmatic evidence that time spent implementing SEL will enhance the academic success of students (…) Current research shows that SEL programs contribute to the academic success of students as well as to their health and wellbeing.”*(1)p.54

**Inputs**

The main input of the ROE programme are the facilitated baby visits, supported by a programme-trained instructor. These cover a variety of areas related to the baby’s development, needs and “temperament”, followed by structured discussions.

*“Learning originates from the baby visits, with themes developed throughout the year in a curriculum that is designed across the years K–8. Connections are made with the learning areas of literacy, writing, art, music, mathematics and science. Discussion of child abuse, foetal alcohol spectrum disorder and violence is facilitated by the instructor with the classroom teacher's support.”*(1)p.53

**Mechanisms of change**

There are two overarching themes that are suggested to drive the outcomes of the programme. The first is the development of emotional intelligence through ROE. The second is the establishment of connectedness through school through children’s perceptions that classrooms are safe and caring. These two are not mutually exclusive, however. The SEL skills, including emotional intelligence, that are developed through the programme facilitate the establishment of connectedness to school.

*Developing emotional intelligence*

The authors suggest that children with social and emotional challenges struggle to learn academically.

*“A child who is distressed has a limited capacity to hear, understand or engage in learning, so for optimal learning the child‘s brain must be attuned to the environment, attentive, focused, motivated and engaged.”*(1)p.66

The authors cite the work of Goleman,(2) whereby emphasis is placed on the role of emotional intelligence in students’ learning, which helps students to develop self-control, endurance and determination.

“*Developing emotional intelligence is about teaching skills that make the learner confident, motivated, aware of expected behaviour, able to manage personal impulses, seek help when appropriate, wait their turn, be aware and effectively interact with others.”*(1)pp.55–56

*Establishing connectedness to school*

Through interaction with the baby over the course the school year, students are expected to develop empathy and awareness of others’ needs and emotions, which promotes understanding of concepts like social inclusion and consensus-building.

*“Children learn messages of social inclusion … to respect and build consensus … [about] how to contribute to a safe and caring classroom … [and ] about emotional literacy.”*(from Gordon 2005 p.6 in 1 p.61)

Through the improvement of these SEL competencies, students also become better at learning academic concepts through: “*increased engagement in learning, with greater attachment and commitment to schooling resulting in improved academic performance and life success.*”(1)p.57

Briefly mentioned, although not described within the context of the programme, is the importance of creating school environments that foster SEL skill development by providing opportunities for students to practice and role model them.

The authors list the key SEL competencies that should be fostered in school with the use of the ROE programme by drawing on the Collaboration for Academic, Social and Emotional Learning (CASEL) framework. These include building competencies in a wide range of areas such as: self-awareness; social awareness; responsible decision-making; self-management; and relationship management. However, these are just presented as a list of desirable attributes to develop, and if and how they may be developed through ROE is not explained. Therefore, we have not highlighted these aspects in the proposed logic model in Figure 1 unless specifically drawn out by the authors.

**Outcomes**

Broadly, the ROE curriculum is expected to help children navigate and control their emotions for better conflict resolution and, ultimately, less violence. The authors cite evidence of reduced bullying as a result of the programme.

“[*ROE]* *significantly improved the attitudes, knowledge and social emotional competencies of all participating teachers and children. A decrease in the frequency of bullying and an increase in pro-social behaviours were reported by all participants. Partnerships between teachers, children, parents and the school community strengthened.*”(1)pp.68–69

**References**

1. Cain G, Carnellor Y. 'Roots of Empathy': A research study on its impact on teachers in Western Australia. The Journal of Student Wellbeing. 2008;2(1).

2. Goleman D. Emotional Intelligence. Why It Can Matter More than IQ. Learning. 1996;24(6):49-50.

**Second Step**

The intervention includes teaching in risk and protective factors linked to aggression and violence, including training on empathy, emotion regulation, communication skills, and problem-solving strategies.(1-5)


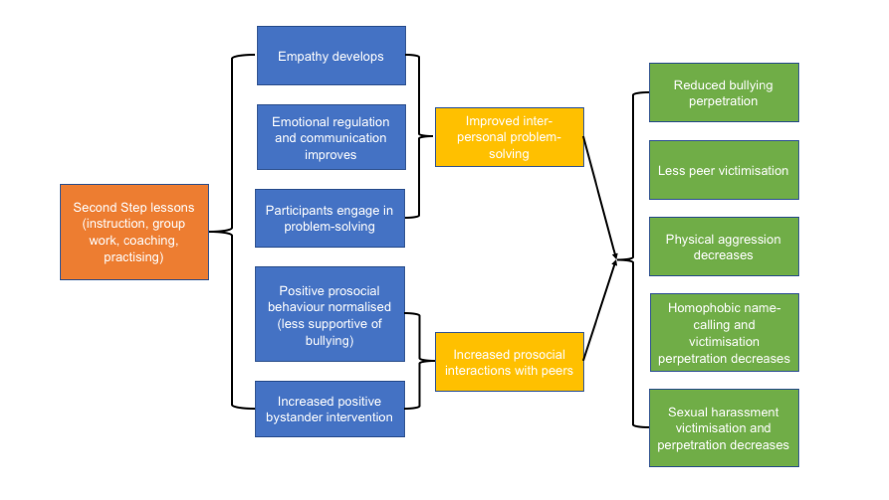


**Figure 1.** Proposed logic model for Second Step

Orange = intervention inputs; yellow = proximal outcomes; green = distal outcomes (impacts); blue = mechanisms expected to facilitate outcomes

**Inputs**

Students learn and practice skills through dyadic and group activities. Lessons are scripted and interactive, including group discussions and activities, class instruction and discussions, dyadic and individual work. Lessons are supported by a DVD. Drawing on Bandura's social learning theory,(6) lessons focus on skills-based and focus on cueing and coaching. The use of group and other collaborative work should increase skills by allowing students to practice in a supportive environment.

**Mechanism**

Students are taught cognitive–behavioural strategies to cope with stress and regulate emotions so are less likely to engage aggressively with peers. Work focuses on promoting empathy (discriminating affective cues in others; perspective-taking; responsiveness to others’ emotions), communication (active listening; assertive communication), emotional regulation (cognitive behavioural strategies to cope with stress and regulate emotions), problem-solving (encoding, representing and interpreting situational cues; identifying and selecting possible responses to a situation; and enacting these), awareness of bullying in peer context (perspective taking skills and empathy for victims; responsibility as bystanders; and positive responses to erode peer support for bullying) and awareness of sexual harassment (definitions, assertive refusal skills).

**Outcomes**

The major outcomes are bullying perpetration, peer victimization, as well as homophobic name-calling and sexual harassment victimization and perpetration.

**References**

1. Espelage DL, Low S, Polanin JR, Brown EC. The impact of a middle school program to reduce aggression, victimization, and sexual violence. Journal of Adolescent Health. 2013;53:180-186.
2. Espelage DL, Low S, Polanin JR, Brown EC. Clinical trial of Second Step middle-school program: impact on aggression & victimization. Journal of Applied Developmental Psychology. 2015;37:52-63.
3. Espelage DL, Low S, Van Ryzin MK, Polanin JR. Clinical trial of Second Step middle school program: impact on bullying, cyberbullying, homophobic teasing, and sexual harassment perpetration. School Psychology Review. 2015;44(4):464-479.
4. Farrell AD, Mehari KR, Kramer-Kuhn AM, Mays SA, Sullivan TN: A qualitative analysis of factors influencing middle school students' use of skills taught by a violence prevention curriculum. Journal of School Psychology. 2015;53(3):179-194.
5. Low S, Smolkowski K, Cook C: What constitutes high-quality implementation of SEL programs? A latent class analysis of second step implementation. Prevention Science. 2016;17(8):981-991.
6. Bandura A. Self-efficacy: Toward a unifying theory of behavioral change. Psychological Review. 1977;84:191–215.

**Steps to Respect**

**
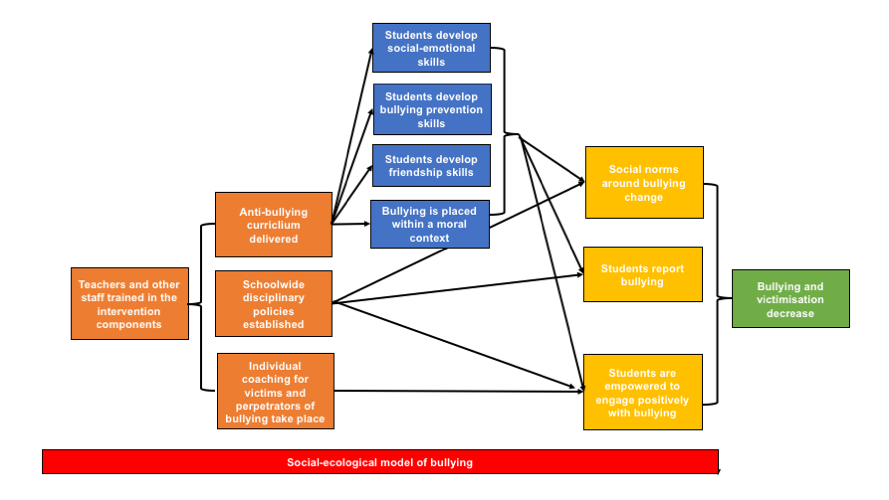
**The Steps to Respect programme has both classroom and schoolwide components and is intended to reduce bullying and victimisation. The classroom component involves a literature-based curriculum that teaches students a number of bullying prevention skills, social-emotional skills and friendship skills. Additionally, children are taught how to engage as bystanders of bullying and how to report bullying. At the school level, there are policies established to change disciplinary action towards bullying, including increased monitoring of bullying and providing individual coaching for victims and perpetrators of bullying. The school climate overall is expected to become more positive, and together, these components are intended to change social norms around bullying. Figure 1 below provides a proposed logic model for the programme.

**Figure 1.** Proposed logic model for the Steps to Respect programme

Orange = intervention inputs; yellow = proximal outcomes; green = distal outcomes (impacts); blue = mechanisms expected to facilitate outcomes; red = underlying theoretical influences

**Theoretical positioning**

The intervention is aligned with the social-ecological model of bullying, which suggests that bullying behaviour is shaped by contextual factors. Therefore, this intervention has components at different levels in order to address these factors. In particular, peer attitudes and social norms around bullying are felt to determine bullying behaviour, and are given particular focus within this intervention.

**Inputs**

There are four inputs within this intervention. The programme begins with the training of teachers and administrators in the intervention’s materials. As the intervention has a schoolwide approach, inclusive of classrooms and broader school policy, training focuses on these two aspects. The goal is to change the way bullying is thought of and approached.

*“A core instructional session for all school staff aims to dispel myths suggesting that bullying is inconsequential or usually perpetrated by easily identified “problem” students. Staff receive an overview of program goals and key features of program content. Teachers, counselors, and administrators receive additional training in how to coach students involved in bullying episodes. Third through sixth-grade teachers are provided with a curriculum orientation. They practice role-playing exercises, an often omitted aspect of classroom lessons reporting students and encourage socially responsible actions.”*(1)p.468

Following from training is the introduction of a new set of school-wide policies around bullying, which are intended to form the basis of new and effective disciplinary policies for students who are engaged in bullying, to monitor students involved in bullying and to improve intervening within bullying situations to ultimately foster a positive school climate and establish positive norms around bullying.

Next there is the classroom curriculum itself.

*“Classroom curricula target the upper three elementary grades and are intended to promote socially responsible norms and behavior and increase social-emotional skills. Lessons help students recognize bullying, increase empathy for students that are bullied, build friendship skills to increase protective social connections, improve assertiveness and communication skills to help students deter and report bullying, and teach appropriate bystander responses to bullying.”*(2)p.425

Finally, there are individual coaching sessions, tailored to students who are involved in bullying, to work with the student to find solution-orientated approaches to reducing bullying. There are sessions for perpetrators and victims.

**Mechanisms of change**

The mechanisms of change correspond to the student or school level. However, in either case, the intervention targets children in their later primary school years.

“*Bullying, and the rewards thereof, increase at the end of elementary. Acceptance of aggression also increases and begins to stabilize then. Because behavior, beliefs, and patterns of interaction that are longstanding become increasingly resistant to change, the Steps to Respect program targets the upper elementary school years as a particularly important time to influence bullying-related skills, beliefs, and behavior.”*(1)p.467

At the level of the student, classroom lessons are used in order to teach social-emotional skills like empathy, self-management and conflict resolution and to use cognitive-behavioural techniques—namely placing bullying within a moral context—in order to help change social norms around bullying. In addition, bullying prevention skills such as assertive responses to bullying, help-seeking and problem-solving to avoid future problems are emphasised. Friendships are recognised as helping to buffer bullying, and as such, lessons also provide opportunities for students to practice friendship skills. The authors are not clear about what these skills may be.

“*Because bullying is a social process strongly influenced by the reactions and behaviors of peers, the program seeks to change attitudes about the acceptability of bullying through clearly labeling bullying behavior as unfair and wrong, increasing empathy for students who are bullied, and educating students about their responsibilities as bystanders to bullying.”*(2) p.425 It is important that children change their mentality around reporting bullying, which the authors describe as “telling to keep someone safe”, versus tattling in order to get someone into trouble. Further, teachers reiterate that bullying is not harmless or that certain people deserve to be bullied on the basis of their personal characteristics.

For those students engaging in bullying who end up receiving individual coaching sessions, it is expected that they will be empowered through these sessions not to bully or to respond constructively when bullied in the future.

At the school level, policies to address bullying are felt to encourage the development of a positive school climate and to change accepted norms around bullying.

However, both individual and school-wide components are expected to work together, as per the social-ecological model of bullying. Ultimately, “*the program is designed to reduce bullying in part through decreasing peer reinforcement of bullying behavior through increased positive bystander behaviors such as ignoring bullying, supporting students who are bullied, intervening to stop bullying incidents, and reporting bullying to school staff*.”(2)p.425

**Outputs**

Given its multi-component design, unsurprisingly, there are expected outcomes at different levels. At the individual level, students are expected to have improved social competence, better connections and relationships with their peers, experience less peer rejection and have greater confidence in addressing bullying.

At the peer level, bystander behaviour—and willingness to intervene in bullying—is expected to improve alongside less support for bullying, greater support for bullied students and better attitudes and willingness to report bullying.

At the school levels, positive norms against bullying are expected, including staff willingness to intervene in bullying and engage in coaching of students who are bullies or victims of bullying.

Overall, these outcomes are expected to come together to lead to the distal outcomes of reduced bullying and victimization and “improved school climate and school connectedness.”(2)p.427

**References**

1. Frey KS, Hirschstein MK, Edstrom LV, Snell JL. Observed reductions in school bullying, nonbullying aggression, and destructive bystander behavior: A longitudinal evaluation. Journal of Educational Psychology. 2009;101(2):466.

2. Brown EC, Low S, Smith BH, Haggerty KP. Outcomes from a school-randomized controlled trial of steps to respect: A bullying prevention program. School Psychology Review. 2011;40(3):423.

**The Gatehouse Project**

The Gatehouse Project was *“designed to make changes in the social and learning environments of the school and at the individual level by providing schools with strategies to enhance students’ sense of connectedness to school and increase individuals’ skills and knowledge for dealing with the everyday life challenges.”*(1)p.252 In the synthesis, the theme that has emerged more strongly relates to developers’ hypothesis regarding the role of students’ attachment to each other, to teachers and to their schools. These attachments are seen to facilitate the development of social connectedness, security, trust and positive self-regard. Ultimately, it was posited that such changes would improve students’ emotional wellbeing and reduce their substance use.


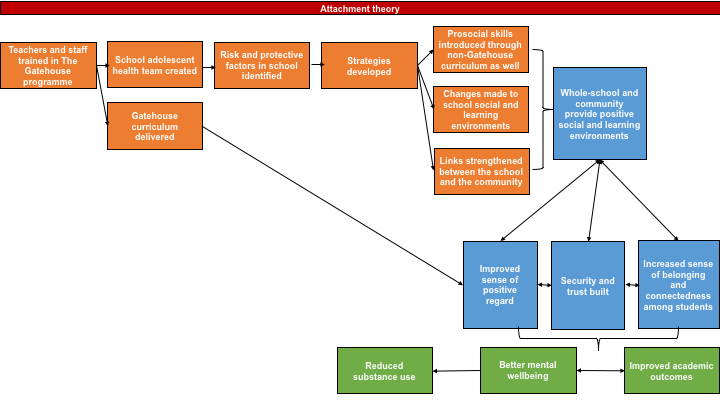


**Figure 1.** Logic model for The Gatehouse Project

Orange = intervention inputs; green = distal outcomes (impacts); blue = mechanisms expected to facilitate outcomes; red = underlying theoretical positioning

**Underlying theoretical positioning**

A fundamental theme in the theory of change for the Gatehouse Project relates to discussions of John Bowlby’s *attachment* theory.(2) This theory posits that negative mental health (such as anxiety and depressive symptoms) are rooted in poor interpersonal bonding and attachment. It follows that the perception of secure interpersonal attachments can enable positive psychological and social development. Authors state that *“three facets of the social context: security, communication and participation underpin an individual’s sense of attachment and were the major focus in the Gatehouse Project.”*(3)p.4

Within discussions of this perspective, authors also place emphasis on the importance of the social environment and the interpersonal bonds it facilitates. The quality of these relationships, in turn, can positively or negatively impact a sense of belonging and responses to challenges.

*“The extent to which an individual has robust social ties is likely to have a direct influence on self-concept and sense of belonging, and, in turn, reactions to social stressors. Conversely, the experience of ongoing insecurity and threat has a detrimental effect on emotional wellbeing. An individual’s capacity to deal with adversity is in part dependent on the availability of support in the immediate social environment, as well as the skills he or she has for making appropriate connections at times of stress*.”(4)p.587

For the Gatehouse Project theory of change, authors use Bowlby’s theory to highlight in particular the importance of students having a positive sense of attachment to their school, teachers and peers. Specifically, engagement with “supportive others” is believed by the authors to prevent “emotionally hazardous events”.(4)

In Figure 1, aspects of attachment theory are most clear in the mechanisms related to belonging and connectedness, and also to building security and trust. These refer to a student’s relationship with peers, teachers, the whole-school environment and also their families.

Other underlying theories only very briefly mentioned by the Gatehouse Project authors include: health promotion,(5) positive youth development,(6) social support theories (7) and the risk and protective factor framework.(8, 9) However, none of these are explained in any detail.

**Programme inputs**

The engagement of the whole-school is encouraged through The Gatehouse Project through the training of staff and the establishment of a school adolescent health team. This team is responsible for identifying risk and protective factors in the school environment and designing strategies to address these factors. It is expected that these strategies will help to infuse aspects of the Gatehouse curriculum throughout the overall curriculum delivered in the school. Further, staff are coached to design strategies that make positive changes to both the social and learning environments and that the school will also firmly establish positive links with the community. The authors do not make any links to the theoretical rationale for these specific inputs, but do emphasise the importance of a whole-school approach. We return to this theme later in our discussion of key mechanisms of change for the programme.

*“The whole school change component emphasized the development and implementation of strategies that addressed these three areas of action at multiple levels of the school community. These three foci provided schools with ‘lenses’ to view and critique what they were currently doing and what they might do to promote these. The comprehensive whole school strategy, therefore sought to: introduce relevant and important skills through the curriculum; make changes in the schools’ social and learning environments; and strengthen links between the school and its community.”*(1)p.252

An additional key programme input is that teachers are trained in the Gatehouse curriculum, which is then delivered to students, either in English classes, or classes that are specifically dedicated to student health and wellbeing (e.g. health or physical education classes) or within personal development or pastoral care curricula.

A goal of The Gatehouse Project is for the programme to become sustainable and institutionalised. As such, the developers recognised the value in trying to fully integrate the programme into existing curriculum rather than have it as an ‘add-on’. Thus, teachers were trained in the Gatehouse curriculum and given the tools to integrate programme content into mainstream curricula.

*“While we provided examples of teaching and learning strategies we encouraged staff to look for opportunities in activities they already undertook with students or to create new ones in line with the principles of the teaching and learning approach. We therefore encouraged staff to integrate and extend their learnings from these materials beyond set lesson times and into the teaching context.”*(1)p.259

*“The integration of the curriculum component with classroom and whole school context, and the encouragement of teachers to adapt and extend their use of the processes and materials was aimed at embedding the work beyond the project.”*(1)p.260

**Desired outcomes**

The authors do not provide a detailed exploration of desired outcomes for the intervention; rather, they emphasise the development of emotional wellbeing in students. Authors then suggest that improvement in emotional well-being leads to a reduction in alcohol and drug use, which are associated with wellbeing. Authors do not clearly elaborate on this link between emotional wellbeing and substance use in their discussion of the programme’s theory.

**Mechanisms of change expected to facilitate outcomes**

There are four mechanisms that emerged in the functioning of The Gatehouse Project. These are: developing a sense of belonging and social connectedness; a whole-school approach; building security and trust; and building positive self-regard. However, only the first two of these mechanisms can be considered as main themes, with building security and trust, and positive self-regard as subthemes seen to contribute to or be influenced by the other two.

*1. A whole-school approach*

An important theme in the Gatehouse Project literature relates to the important role schools are seen to play in promoting mental health and positive development of young people. As such, programmes like The Gatehouse Project can provide a platform from which schools can carry out this task.

*“The scope for siting curricula for promoting mental health in schools has grown as schools have come to play a broader role in equipping young people for adult life; schools have a greater readiness to promote and foster critical and reflective skills, problem-solving abilities and to encourage collaborative work. These skills have relevance not only for academic and workplace learning, but also for social and emotional development. This broader conception of the role of schools provides the scope for introducing curriculum modules focusing on the cognitive and interpersonal skills underlying emotional wellbeing.”*(4)p.591

Authors recognize that the disengagement of youth from their academic learning is often moderated by their social and emotional wellbeing. Further, a connection to their classrooms and school communities can foster a sense of belonging that facilitates better academic performance.

*“There are signs that health and educational agendas are converging. There has, for example, been an increasing interest in the forms of schooling that might best meet the educational needs of early adolescents. The call for reform and restructure in the middle years of schooling (10–15 years of age), has been driven by the identification of alienation or disengagement of young people as the major barrier to educational success in the younger teens. The extreme consequences of alienation are seen in young people who ‘drop out’ of school or have high levels of truancy or absenteeism. This same group are those that commonly have the worst mental health outcomes.”*(4)p.588

Further, strong links to the community are also suggested to be part of the whole-school approach, to establish a continuum of positive social and learning environments to which students are exposed.

*2. Developing a sense of belonging and social connectedness*

Closely aligned with assertion in attachment theory that social ties positively influence a sense of belonging and social connectedness, such ties are encouraged throughout The Gatehouse Project. Further, authors note the mutually reinforcing effects of a sense of belonging and connectedness with improved emotional and learning outcomes. Patton (4) suggests that feelings of belonging or connectedness and emotional wellbeing as well as improved learning outcomes all influence each other. A sense of belonging/connectedness influences and is influenced by students’ security, communication and positive regard.

It is expected that, with greater social connections, students would develop their capacity to deal with adversity by being able to draw from support in their social environment.

*3. Building security and trust*

This mechanism is an emerging sub-theme, strongly linked to the over-arching, and commonly cited, mechanism related to developing a sense of belonging and social connectedness. Specifically, developing these are felt to then lead to a sense of security and trust in young people, which facilitates positive mental health outcomes.

*4. Building a sense of positive regard for oneself*

This positive regard is further felt to be facilitated by valued participation in school, good communication, and development of skills that enable students to make valued contributions to school life. These elements were not however explained in any further detail.

All four mechanisms are understood to both influence and be influenced by one another (indicated through the bidirectional arrows between the mechanisms shown in blue in Figure 1). Together, these mechanisms are predicted to then affect the outcomes of better mental wellbeing and improved academic outcomes, indicated in the green boxes in Figure 1.

**Moderating Factors**

The authors highlight the need for school ownership of the programme and a willingness to invest time and resources in it, in order for it to be effective.

*“To initiate change, schools need to be convinced of the relevance of and need for change, consider the readiness of the staff to become involved and be prepared to allocate school resources, including time*.”(4)p.588

**Assumptions**

One fundamental assumption driving the theory of change is that authors presuppose that improved emotional wellbeing will lead to less substance use in adolescents. Some studies linking depression anxiety and substance use are referenced in the literature to support this claim, but the actual mechanisms supporting this connection are not explored.

As above, the authors also seem to be driven by the optimistic assumption that, contrary to what have been expressed in other interventions, that schools ARE increasingly interested in the broader wellbeing of students. Other interventions suggest the opposite—that there is less time and resources dedicated to non-academic pursuits. One reason for this assumption may be a difference in context. The Gatehouse Project is delivered in Australia, whereas many others are United States-based, where it is clear the “No Child Left Behind” Act of 2001 has emphasised standardised testing and academic achievement above all else.

**References**

1. Bond L, Butler H. The Gatehouse Project: a multi-level integrated approach to promoting wellbeing in schools. Evidence-based public health: Oxford University Press; 2010. p. 250-69.

2. Bowlby J. Attachment and loss: retrospect and prospect. American journal of Orthopsychiatry. 1982;52(4):664.

3. Patton G, Bond L, Butler H, Glover S. Changing schools, changing health? Design and implementation of the Gatehouse Project. Journal of Adolescent Health. 2003;33(4):231-9.

4. Patton GC, Glover S, Bond L, Butler H, Godfrey C, Pietro GD, et al. The Gatehouse Project: a systematic approach to mental health promotion in secondary schools. Australian and New Zealand Journal of Psychiatry. 2000;34(4):586-93.

5. Bandura A. Health promotion from the perspective of social cognitive theory. Psychology and health. 1998;13(4):623-49.

6. Benson PL, Scales PC, Hamilton SF, Sesma A. Positive youth development: Theory, research, and applications: Wiley Online Library; 2006.

7. Lakey B. Social Support Theory. Social support measurement and intervention: A guide for health and social scientists. 2000:29.

8. Bond L, Toumbourou JW, Thomas L, Catalano RF, Patton G. Individual, family, school, and community risk and protective factors for depressive symptoms in adolescents: a comparison of risk profiles for substance use and depressive symptoms. Prevention science. 2005;6(2):73-88.

9. Duncan SC, Duncan TE, Strycker LA. Risk and protective factors influencing adolescent problem behavior: A multivariate latent growth curve analysis. Annals of Behavioral Medicine. 2000;22(2):103-9.

**Youth Matters**


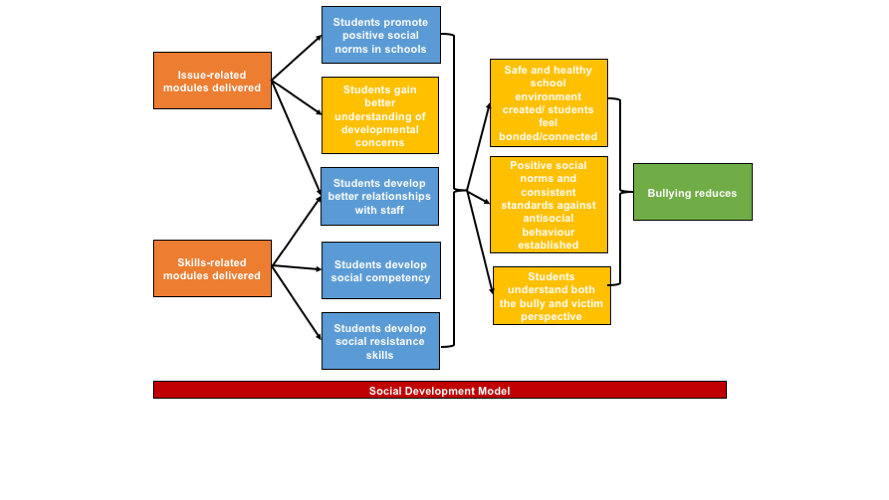
Youth Matters uses a series of instructional literature-based modules to reduce antisocial behaviour in children, with a focus on bullying. In Figure 1, we present an overall logic model for the programme.

**Figure 1.** Proposed logic model for the Youth Matters Programme

Orange = intervention inputs; yellow = proximal outcomes; green = distal outcomes (impacts); blue = mechanisms expected to facilitate outcomes; red = underlying theoretical positioning

**Key theoretical concepts**

Youth Matters is based on the social development model (1) and its linked notion that antisocial behaviour in children can be prevented by intervening in four key areas of development: *“(1) bonding, defined as attachment and commitment to family, school and positive peers; (2) belief in the shared values or norms of these social units; (3) external constraints such as clear, consistent standards against antisocial behavior; and (4) social, cognitive and emotional skills that provide protective tools for children to solve problems, assertively and confidently perform in social situations, and resist influences and impulses to violate their norms for behaviour.”*(2)p.287

**Inputs**

A range of instructional modules are delivered in Youth Matters, which are divided in interactive ‘issues’-related and ‘skills’-related modules.

Each module can be integrated into the English or health education curriculum through the use of literature.

“*A 30–40-page story that is designed to help schools meet academic standards in both health education and [English]. The substantive content of each story is directly linked to skills taught in the curriculum modules.”*(2)p.287

**Mechanisms of change**

As an overall theme, consistent with the first and second targeted aspects of the social development model, the program seeks to encourage positive relationships between students and adults in the school and to promote a positive (particularly, a ‘safe and healthy’) school and community environment. How these relationships are built and sustained, however, is not explored by the authors. As such, in Figure 1, the relationships between the curriculum and students developing better relationships with staff is presumed. Similarly, the relationships between these relationships, establishing positive school environments and the reduction of bullying are not well explained and those suggested in Figure 1 are also our extrapolations.

As above, modules are issue- or skills-related, with different mechanisms of change suggested for each.

In issue-related modules:

“*students discuss critical developmental concerns (e.g., being a good friend, teasing and bullying, building empathy, risks and norms surrounding aggression, etc.) and create projects that promote positive norms in school.*”(2)p.287

In skills-related modules:

“*students learn social competency and social resistance skills (e.g., asking for help, making better choices, standing up for yourself and others, preventing bullying behaviors, coping with bullying, etc.) that they can use to stay out of trouble, build positive relationships, make good decisions, and avoid antisocial behaviour.*”(2)p.287

Through both modules, “*understanding the consequences of bullying from both a bully and victim perspective is emphasized”*. (p.287) The modules also conclude with students’ creating school-wide projects that underscore the negative impact of bullying and aggression.

**Outcome**

Attending the activities delivered by the Youth Matters programme allows students to gain comprehensive perspective of bullying which includes the points of view of the perpetrator and of the victim. Ultimately there is the expectation that this understanding will lead to a reduction in bullying. However, the theory of change for the Youth Matters programme does not provide an explanation as to how the understanding of bullying gained through the modules translates into a reduction in bullying and aggressive behaviour in schools as outcomes of the intervention.

**References**

1. Catalano RF, Hawkins J. The social development model: a theory of antisocial behavior. In: Hawkins J, editor. Delinquency and Crime. New York: Cambridge University Press; 1996. p. 149–97.

2. Jenson JM, Dieterich WA. Effects of a skills-based prevention program on bullying and bully victimization among elementary school children. Prevention Science. 2007;8(4):285-96.
